# Enhanced spin-to-charge conversion in epitaxial La_0.67_Sr_0.33_MnO_3_/NdNiO_3_ bilayers at the nickelate metal-insulator phase transition

**DOI:** 10.1038/s41467-026-75812-0

**Published:** 2026-08-06

**Authors:** Biswajit Sahoo, Sarmistha Das, Akilan K, Katherine Matthews, Melissa Yactayo, Robin Glefke, Xiaoke Li, Hao Zheng, Sebastien Petit-Watelot, Juan-Carlos Rojas-Sánchez, Alexandre Pofelski, Yimei Zhu, Alex Frano, Eric E. Fullerton

**Affiliations:** 1 Center for Memory and Recording Research, University of California San Diego, La Jolla, CA USA; 2 Department of Physics, University of California San Diego, La Jolla, CA USA; 3 Université de Lorraine - CNRS (UMR 7198), Institut Jean Lamour, Nancy Cedex, France; 4 Argonne National Laboratory, Lemont, IL USA; 5 Brookhaven National Laboratory, Condensed Matter Physics and Materials Science Department, Upton, NY USA; 6 Program in Materials Science and Engineering, University of California San Diego, La Jolla, CA USA

**Keywords:** Spintronics, Magnetic properties and materials

## Abstract

Phase transition materials such as NdNiO_3_ (NNO) when coupled with low damping ferromagnets such as La_0.67_Sr_0.33_MnO_3_ (LSMO) hold the promise for developing new multi-functional material systems harnessing the interplay of charge, spin, and orbital degrees of freedom. In this study, we probe the evolution of spin-to-charge conversion in epitaxial all-oxide LSMO (12 nm)/NNO (4, 8, and 16 nm) bilayers. Using spin pumping ferromagnetic resonance, we track the spin-to-charge conversion within the NNO layer through the paramagnetic metal to antiferromagnetic insulator transition and observe a pronounced enhancement of the inverse spin Hall effect signal at the onset of this phase transition. We attribute this enhancement to the electronic and magnetic disorder in NNO at the first-order phase transition, thereby providing insights into the mechanism of spin transport through the phase transition. The tunability of spin-to-charge conversion in this low damping bilayer system offers a pathway for developing reconfigurable, energy-efficient spintronic devices.

## Abstract (web summary)

The authors observe disorder-driven enhancement of charge to spin conversion in LSMO/NdNiO_3_ bilayer, which is at the onset of the first-order metal-insulator phase transition of NdNiO_3_ due to enhancement in both intrinsic and extrinsic spin Hall effects.

## Introduction

The correlated interactions of spin, charge, orbital, and lattice degrees of freedom in perovskite transition metal oxide (Pv-TMO)-based heterostructures offer a fertile platform to explore new physics and realize novel device functionalities beyond what conventional metallic systems can offer^1–3^. Pv-TMOs, especially rare earth nickelates like NdNiO_3_ (NNO), have garnered special attention due to their first-order metal- insulator transitions (MIT), which can be accompanied by intriguing phenomena such as simultaneous magnetic^4–6^ and/or structural (bond-order)^5,7,8^ phase transitions and mixed phase coexistence with domain formation^1–5^. The onset of these MITs involves sparse insulating domains that first nucleate within a metallic matrix, and such transitions can be tuned by electron doping^6,9,10^, strain engineering^7,8,11–18^, thickness variation^19–21^, and application of a gate voltage^22–24^. The rich physics behind these first-order phase transitions, coupled orders, and tunability via strong correlations makes Pv-TMOs appealing candidates for multifunctional, reconfigurable spintronic device structures.

The richness of these Pv-TMO systems is further enhanced by layering multiple such materials in thin film heterostructures, adding an additional level of complexity due to the structure and interactions at the interface between layers. For example, interfacing NNO with a manganite ferromagnet has been shown to induce charge transfer effects^25^ and proximity-induced novel ferromagnetism in NNO^26–29^. The origin of these interfacial properties is primarily governed by the structure and dimensionality of the individual layers, which are directly linked through oxygen octahedral connectivity at the interface^30,31^. The degree of octahedral bending and rotation critically influences the interfacial electronic and magnetic exchange interactions, which in turn depend strongly on the thickness, strain state, and the nature of the transition metal cations involved^32^. The sensitivity of these interfacial interactions to perturbations in each individual layer allows for the selective modification of heterostructure properties through multiple tuning knobs. With atomically sharp and structurally coherent interfaces, Pv-TMO heterostructures exhibit structure-controlled interfacial phenomena that remain intriguing and largely unexplored for device applications.

While their first-order transitions and interlayer coupling have been well characterized, Pv-TMO heterostructures provide numerous opportunities for studying the influence of these phenomena on spin transport and magnetization dynamics. Materials like NNO that have coupled first-order electronic and magnetic transitions offer an especially rich platform for such study, as the interaction between spin current and heterogeneous domains may provide new insights into spin-to-charge conversion mechanisms that do not operate in homogeneous phases. By layering NNO with an adjacent ferromagnetic manganite that can inject spin currents into the nickelate layer, we can investigate how domain nucleation and real space inhomogeneities influence spin and charge transport. Because spin pumping involves the entire structure, we can see how changes in the NNO layer – like its resistance, magnetic order, structure, or thickness – affect the system’s overall spin transport and dynamics. Understanding how spin currents respond to first order electronic and magnetic transitions opens new routes to controlling coupled interfaces and developing phase transition-based devices from a wide class of heterogeneous correlated materials.

Here we report on spin dynamics in epitaxial La_0.67_Sr_0.33_MnO_3_ (LSMO)/NNO bilayers, where we track the rather unexplored spin-to-charge conversion in NNO as it undergoes its MIT (*T*_*MIT*_ = 200 K in bulk)^33^. We observe thickness- and temperature-dependent modulation of spin-charge conversion within the NNO layer. More importantly, we observe an enhanced inverse spin Hall effect (ISHE) at the onset of NNO’s MIT, which has not been observed in other materials with first-order electronic phase transitions. Our findings reveal how the NNO phase transition modulates spin transport across the LSMO/NNO bilayer and identify mesoscopic domain heterogeneity in NNO as an underlying driver of this enhancement. These tunable interactions can drive advancements in magnetism-based devices, such as magnetic tunnel junctions and spin-based nano-oscillators. Controlling spin-charge conversion in such oxide heterostructures enables reconfigurable spintronic devices that leverage correlated electron physics absent in metallic systems^34–39^.

## Results

**Fig. 1:**
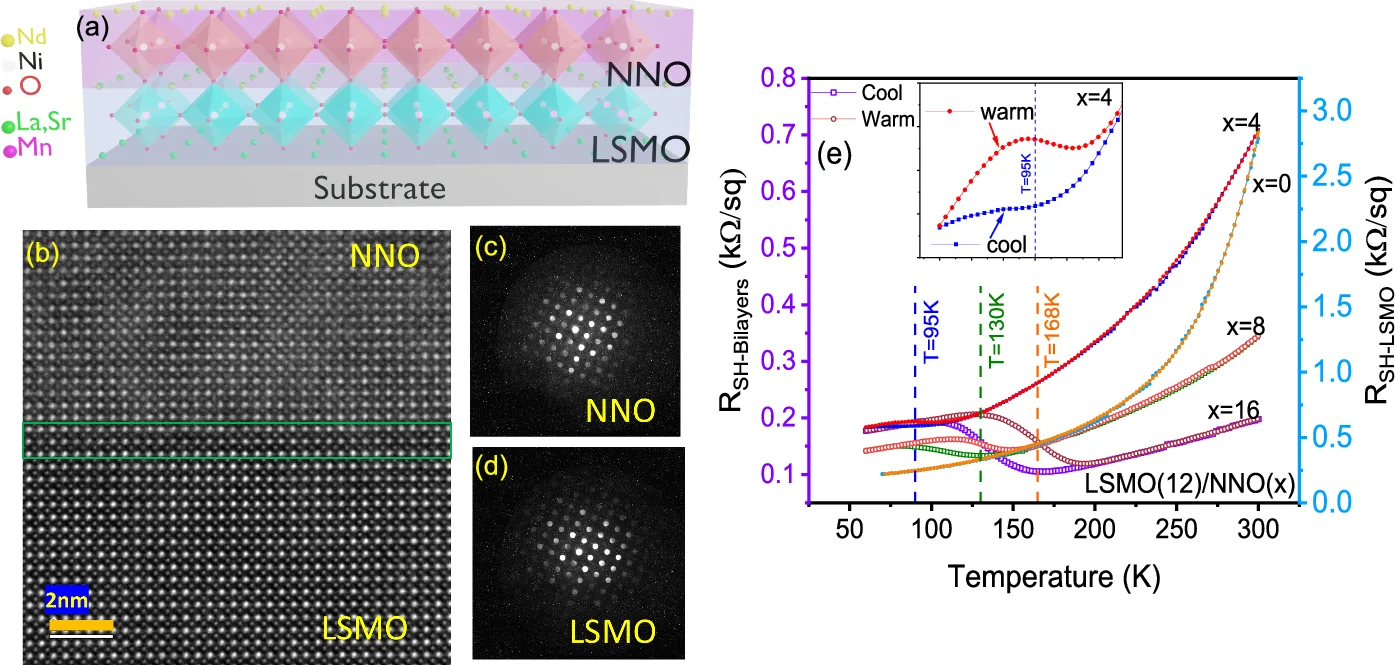
Structural and electronic characterization. **a** Shows a schematic of the epitaxial LSMO/NNO bilayer. **b** High-resolution cross-sectional TEM image of an LSMO/NNO bilayer along the NGO < 001> direction showing an atomically sharp interface with coherent growth of NNO on LSMO demarcated by the green box. (**c**, **d**) are the respective electron diffraction patterns. **e** Resistance vs temperature variation of samples LSMO(12)/NNO(0-16), with the T_MIT_ in the cooling phase shown by dashed reference lines. The lower curves in each loop are from the cooling cycle. Inset shows a zoomed-in view of the hysteretic RT loop in the cooling and warming cycle for LSMO(12)/NNO(4).

We study LSMO(12 nm)/NNO(4, 8, and 16 nm) bilayers (Fig. 1a) that were grown in-situ using pulsed laser deposition (for more details see the **Methods** section) onto single-crystalline NdGaO_3_ (NGO) substrates with orientation (*110*)_Pbnm_. NGO substrates were used because NGO has a low lattice mismatch with both LSMO (compressive) and NNO (tensile) and allows for growth of low-damping LSMO layers^40,41^ with a thickness-dependent tunability of *T*_*MIT*_ in NNO^17^. The samples’ epitaxial orientation and film quality were evaluated by X-ray diffraction (XRD) (Figure S1), reciprocal space map (RSM) (Fig. S14), and transmission electron microscopy (TEM) (Fig. 1b–d). The XRD spectra shows clear peaks of LSMO(*002*)_pc_ (pc subscript indicates pseudocubic notation, which will be used in the rest of the paper) and NNO(*002*)_pc_, with Laue fringes indicating smooth films with excellent crystalline quality (Fig. S1a). Reciprocal space maps reveal high-quality growth of the bilayers and that the LSMO and NNO layers are both epitaxially strained by the NGO substrate (Fig. S14). TEM imaging of a similarly grown LSMO(17 nm)/NNO(24 nm) bilayer (Fig.1b) shows coherent growth of the LSMO/NNO bilayers, with a chemically sharp interface confirmed by electron energy loss spectroscopy (EELS) (Fig. S2b–h). The electron diffraction pattern of NNO and LSMO films (Fig. 1c, d) further demonstrates the high crystalline quality of the films. We further find that the chemical composition of single-layer LSMO is equivalent to the LSMO in LSMO/NNO bilayers (see Fig. S2b–h)^41^.

Figure 1e shows the resistance (*R*) vs temperature (*T*) variation of the single-layer LSMO and bilayer LSMO/NNO films. The single-layer LSMO film shows the expected semi-metallic behavior for the ferromagnetic (FM) phase of LSMO (Fig. S3f), whereas the bilayers show hysteretic behavior from the first-order MIT of NNO. The bilayer resistance, including the hysteretic behavior, closely follows a simple parallel resistor model of *R*_*LSMO*_ and *R*_*NNO*_ as shown in Fig. S3b. *T*_*MIT*_ of NNO is 168 K, 130 K, and 95 K in the cooling cycle for the LSMO(12)/NNO(16, 8, 4) samples, respectively. Here *T*_*MIT*_ is defined as the temperature marking the onset of the insulating behavior; i.e., where d*R*/d*T* = 0 as outlined in ref.^42^ (Figure S3c–e). We find that *T*_*MIT*_ decreases with decreasing thickness of NNO, consistent with previous reports^12,17,18^. We note that the MIT in our films is less sharp, with less pronounced hysteresis (Fig. S3a), compared to reports on optimally grown single-layer NdNiO_3_^1,42^. This is because our deposition parameters were chosen to preserve simultaneously the MIT in NNO and the low-damping FM state of LSMO in the bilayer. Hence, the NNO layer is not optimized by single-layer standards. Nonetheless, the first-order character of the transition is independently and unambiguously confirmed by the resolvable hysteresis in the (¼ ¼ ¼)_pc_ AFM Bragg peak via resonant soft x-ray scattering, discussed below.

In bulk NNO, the MIT is concurrent with a magnetic transition from paramagnetic metal to an AFM insulating phase. However, in thin films the magnetic phase transition is observed to occur at a Néel temperature (*T*_*N*_) somewhat lower than *T*_*MIT*_^42,43^ where there is long-range AFM order in the film. Because of this known separation of *T*_*MIT*_ and *T*_*N*_ in NNO thin films, we determine the two temperatures using distinct methods: *T*_*MIT*_ is identified from resistance measurements as the temperature at which d*R*/d*T* = 0^44^, as described above. *T*_*N*_ can be determined from transport and magnetization measurements in these heterostructures in two ways. First, in an LSMO(12)/NNO(16) bilayer, we extract the coercivity (H_C_) from magnetization versus field loops performed over a wide temperature range. As a result of interlayer magnetic coupling between the LSMO and NNO layers, the bilayer coercivity is larger than the coercivity of LSMO alone below NNO’s transition temperature. *T*_*N*_ can then be determined as the temperature at which dH_C_/dT changes (*T*_*N*_ = 130 K for LSMO(12)/NNO(16), see Fig. S6d). We also estimate *T*_*N*_ from the peak of d(log(R))/d(1/T) vs T plots as outlined in ref.^42^, where *T*_*N*_ corresponds to the critical threshold for the loss of connectivity in the metallic percolation network^11^, and find a similar value (Fig. S3c–e) with *T*_*N*_ < *T*_*MIT*_.

All the above-mentioned observations for the bilayers derived from the electronic and magnetic properties – like the resistance and coercivity temperature dependences – are distinct from the intrinsic behaviors of the individual constituents of pristine LSMO and NNO. As shown in the TEM imaging in Fig. 1b, the LSMO/NNO interface in these bilayers is sharp and well-defined, which may contribute to the observed interfacial coupling as well as the interlayer spin transport that we will probe in this work. Moreover, these heterostructure-specific phenomena are sensitively modulated by the thickness of the NNO layer, further reinforcing the significance of this bilayer system for our study as one in which the overall heterostructure properties are sensitive to changes in each individual layer^26–29^. Having confirmed the interfacial magnetic coupling, the electronic response, and the paramagnetic-AFM transition + MIT in the bilayer films, we investigate the influence of the phase transition on the spin transport in LSMO/NNO. In particular, we are interested in the spin-to-charge current conversion and its modulation thereof as NNO goes through its MIT.

**Fig. 2:**
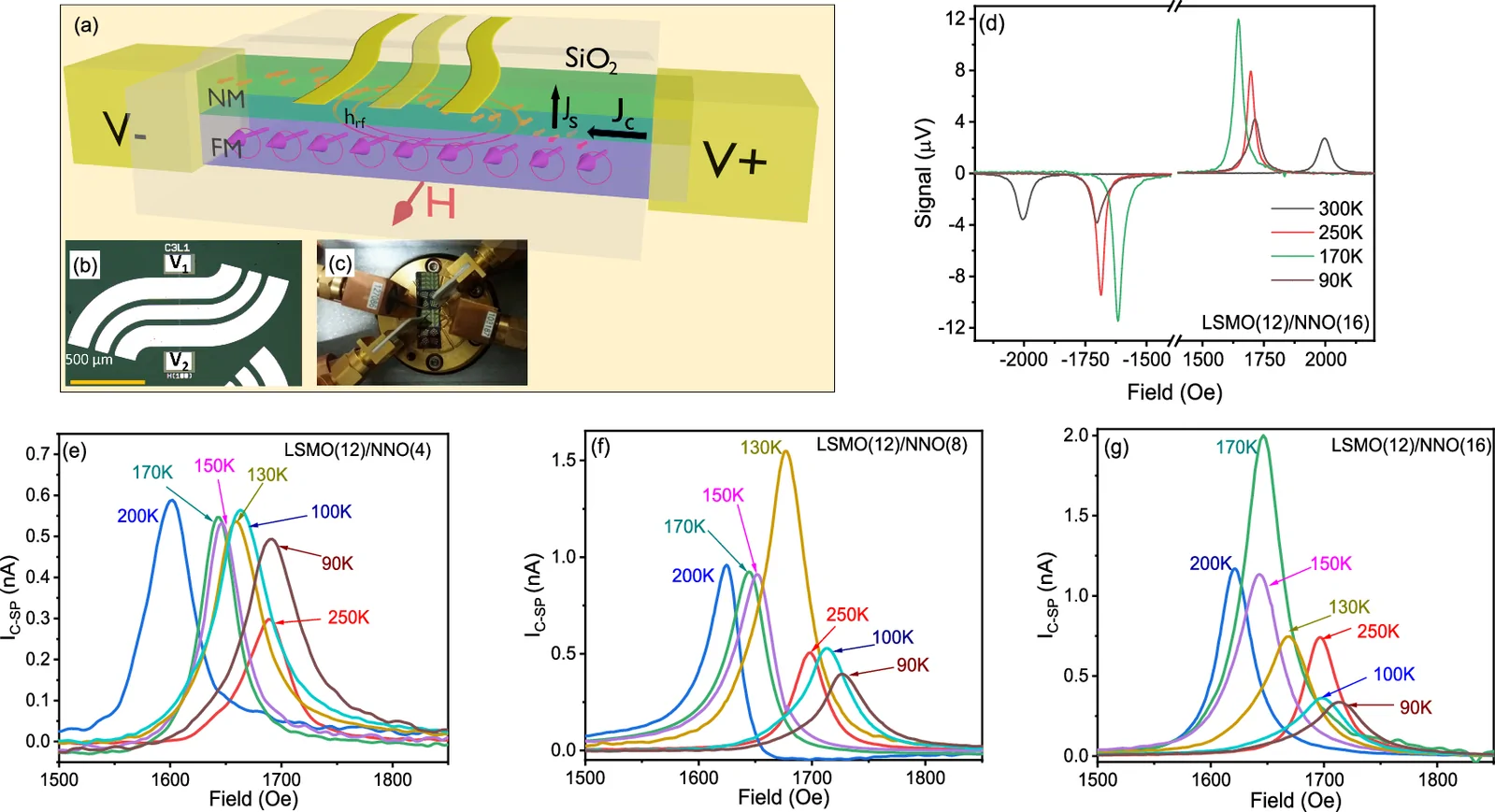
Transport and spin-pumping in LSMO/NNO. **a**–**c** Show the SP-FMR schematic with an optical micrograph of the device (**b**) and the probe placement (**c**). The top left and bottom right are the RF probes, the other two are the voltage probes. **d** Shows the full-field sweep SP-FMR signal for LSMO(12)/NNO(16) at 8 GHz and 15 dBm power, demonstrating a dominant spin-pumping contribution. (**e**–**g**) show the spin-pumped charge current (I_C-SP_) for LSMO(12)/NNO(4-16) at 8 GHz at different temperatures in the cooling cycle demonstrating a distinct increase in the current at the T_MIT_ for LSMO(12)/NNO(8-16). Measurements are performed with H// < 1-10> direction of NGO(110) substrate. The H_res_ initially reduces with temperature before increasing again for T < 200 K, which is due to magneto crystalline anisotropy of LSMO (see Supp. Info.).

We probe the spin-to-charge conversion in NNO via spin-pumping ferromagnetic resonance (SP-FMR) and fabricate micro-devices of 500 µm x 10 µm via a three-step UV lithography (further details available in the **Methods** section). The device and measurement schematic are shown in Fig. 2a–c. In SP-FMR, an external field (*H*) is simultaneously swept perpendicular to the radio frequency (RF) currents in the coplanar waveguides. The RF fields generated by the waveguide excite spin precession of the FM moments, which then pumps a pure spin current into the adjacent layer. This spin current is then converted into charge current within the NNO layer by the inverse spin Hall effect (ISHE) and is probed by the voltage leads. The resulting lineshape is composed of a symmetric and anti-symmetric Lorentzian, with a voltage minimum or maximum at the resonant field (*H*_*Res*_) of the FM, and is fit to the following equation:

1 $${V}_{{total}}={V}_{s}\frac{{\left(\frac{\Delta H}{2}\right)}^{2}}{{\left(\frac{\Delta H}{2}\right)}^{2}+{\left(H-{H}_{{res}}\right)}^{2}}+{V}_{A}\frac{\left(\frac{\Delta H}{2}\right)\left(H-{H}_{{res}}\right)}{{\left(\frac{\Delta H}{2}\right)}^{2}+{\left(H-{H}_{{res}}\right)}^{2}}$$

Here Δ*H* is the linewidth and *V*_*S*_ is the symmetric contribution of the voltage, which has major contributions from spin-pumping and minor contributions, if any, from the planar Hall effect (PHE) and anisotropic magnetoresistance (AMR) within the LSMO layer. *V*_*A*_ is the anti-symmetric part, which consists of a rectification effect dominated by the anomalous Hall effect (AHE). When spin-pumping is the dominant contribution, *V*_*S*_ flips sign for positive and negative field sweeps, whereas the sign does not change for the AMR dominated contribution^45^. Single-layer NGO(*110*)_Pbnm_/LSMO samples do not show spin-pumping dominated behavior^41^.

Figure 2d shows the temperature-dependent SP-FMR voltage signal of LSMO(12)/NNO(16) at 8 GHz in the cooling cycle for select temperatures (90 K < T < 300 K), demonstrating a strong spin-pumping dominated signal exemplified by the symmetric lineshape that flips sign upon reversing the magnetic field (full scans for all temperatures are plotted in the supplementary section Fig. S10b). This is the case for LSMO(12)/NNO(4) and LSMO(12)/NNO(8) as well (full field sweeps are not shown for brevity). In conclusion, the observation of this strong spin-pumping dominated signal in all bilayers, including the one with the lowest NNO thickness of 4 nm, and its absence in single-layer LSMO grown on NGO(*110*)_Pbnm_^41^ clearly indicates that the NNO layer plays a crucial role for the spin-to-charge conversion. The sign of spin-to-charge conversion for NNO is the same as Pt^41^, which is corroborated by the SP-FMR of a Py/NNO bilayer at room temperature as shown in Fig. S10a.

Unlike metallic systems, the resistance of LSMO/NNO bilayers has a significant temperature variation (Fig. 1e) and thus, we evaluate the spin-pumped charge current (*I*_*C-SP*_ = *V*_*SP*_/*R*_*dev*_) for the cooling cycle. Figure 2(d)-(f) show the positive field sweep of the *I*_*C-SP*_ for LSMO(12)/NNO(16, 8, 4). LSMO(12)/NNO(4) with a T_MIT_ = 95 K is expected to be metallic for 95 K < T < 300 K. In this bilayer the *I*_*C-SP*_ increases from 300 K down to 200 K, which can be attributed to the increased spin-current pumped by LSMO due to increased *M*_*s*_ and exchange interaction strength of LSMO with decreasing temperature. The *I*_*C-SP*_ shows a small reduction for T < 200 K, remains mostly constant until 130 K and then decreases slightly again for T < 130 K. Such behavior has been observed in LSMO and LSMO/Pt systems, which has been attributed to increased dissipation of spin currents in the bulk and interface of LSMO for *T* ~ < 110 K^41^.

**Fig. 3:**
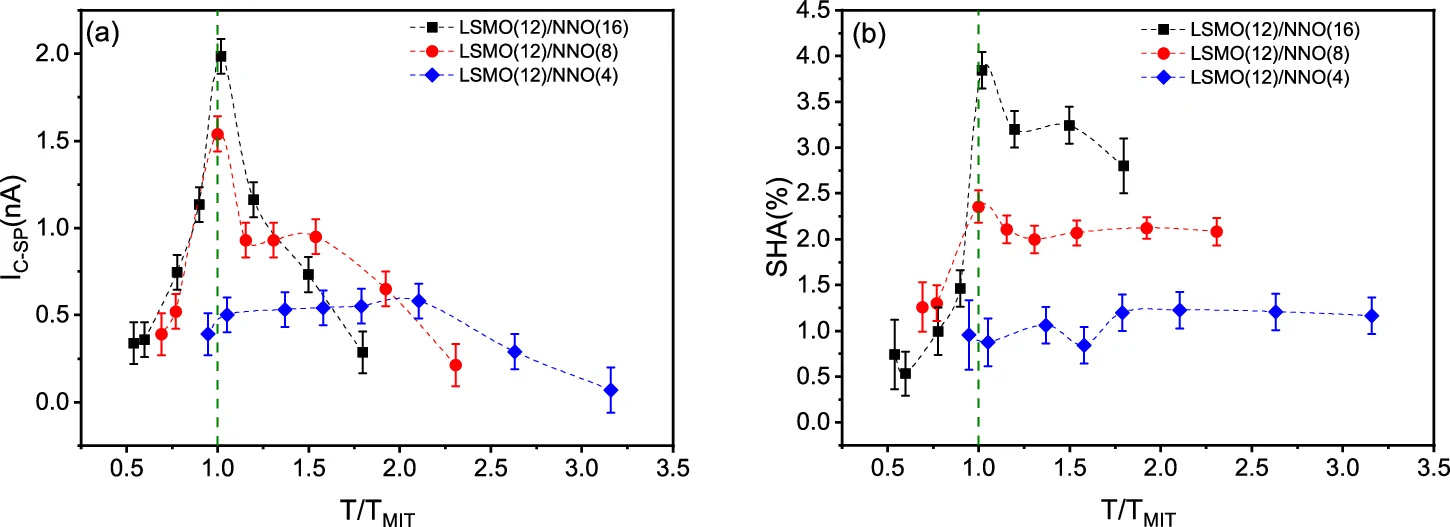
Signatures of enhanced spin pumping at the MIT. **a** Charge current produced by NNO layer pumped from LSMO at 8 GHz as function of normalized temperature T/T_MIT_ for different NNO thickness. **b** Spin Hall angle variation with respect to T/T_MIT_. We see a distinct peak when the temperature reaches T_MIT_ (vertical green dotted reference line). Error bars denote standard error (SE).

The more interesting *I*_*C-SP*_ variation happens for LSMO(12)/NNO(16) and LSMO(12)/NNO(8) where *T*_*MIT*_ is greater than 130 K. For LSMO(12)/NNO(16), *I*_*C-SP*_ increases from 300 K to 200 K with a significant (2-fold) enhancement at 170 K (as seen in Fig. 2g), which corresponds to *T*_*MIT*_ of this bilayer, before dropping to much smaller values for lower temperatures. We observe a similar trend in the *I*_*C-SP*_ for LSMO(12)/NNO(8): *I*_*C-SP*_ increases until 200 K and reduces slightly and remains mostly constant for 170 K < *T* < 150 K, (in accordance with what was observed for LSMO(12)/NNO(4)) and then shows a significant increase at 130 K (*T*_*MIT*_ of LSMO(12)/NNO(8)), before reducing drastically for 90 K < T < 130 K. The variation of *I*_*C-SP*_ for LSMO(12)/NNO(16, 8, 4) with respect to the reduced temperature *T*/*T*_*MIT*_ is plotted in Fig.3a (the results are plotted with respect to temperature in Suppl. Fig. S11a). As can be seen, there is a sharp peak in *I*_*C-SP*_ at *T*_*MIT*_ for the 8 and 16 nm NNO layers followed by a steep fall off for *T* < *T*_*MIT*_. This is our key experimental result: a direct linking of the spin pumping characteristics to the MIT in NNO.

We can further estimate the efficiency of the conversion of spin current to charge current, the spin Hall angle (*θ*_*SHA*_), via the following relation^46–48^:

2 $${I}_{C-{SP}}={\theta }_{{SHA}} \, {J}_{s}w{\lambda }_{{sf}}\tanh \left(\frac{{t}_{{NNO}}}{2{\lambda }_{{sf}}}\right)$$

where *t*_*NNO*_ is the thickness of NNO layer, *w* is the width of the device (10 µm) and *λ*_*SF*_ is the spin diffusion length of NNO. *λ*_*SF*_ was determined by the exponential fitting of the Gilbert damping parameter with NNO thickness (Fig. S9a, b)^49^. We evaluated the spin diffusion length in the metallic regime (*λ*_*SF-M*_, 170 K < *T* ≤ 300 K) to be ≈ 4.1 nm and in the insulating regime (90 ≤ T ≤ 100) to be *λ*_*SF-I*_ ≈ 2.2 nm (see Supp. Info for details). *J*_*s*_ is the spin current density injected at the LSMO/NNO interface and it is determined by the following relation^46–48,50^:

3 $${J}_{s}\approx \left(\frac{\,{g}_{eff}^{\uparrow \downarrow }\hslash }{8\pi }\right){\left(\frac{{h}_{RF}\gamma }{\alpha }\right)}^{2}\left[\frac{4\pi {M}_{eff}\gamma+\sqrt{{(4\pi {M}_{eff}\gamma )}^{2}+{(4\pi f)}^{2}}}{{(4\pi {M}_{eff}\gamma )}^{2}+{(4\pi f)}^{2}}\right]\left(\frac{2e}{\hslash }\right)$$

Here *e* is the electronic charge and *h*_*RF*_ is the RF field experienced by the sample (which was calibrated beforehand for all RF frequencies^51^), *α* is the Gilbert damping (obtained via FMR), and *g*_*eff*_ is the effective spin mixing conductance given as ${g}_{{eff}}^{\uparrow \downarrow }=\frac{4\pi {M}_{s}{{{{\rm{d}}}}}_{{{{\rm{FM}}}}}\Delta {{{\rm{\alpha }}}}}{g{\mu }_{B}}$^44^, where *g* is the electronic g-factor, *µ*_*B*_ is the Bohr magneton, Δ*α* is the difference between the damping of bilayer samples and the LSMO film given by ${{{{\rm{\alpha }}}}}_{{bilayer}}-{\alpha }_{{LSMO}}$ obtained by FMR, ${\alpha }_{{LSMO}}=0.003$, and *d*_*FM*_ is the LSMO thickness. We note that the LSMO in bilayers are grown at 950 °C and is effectively annealed at 710 °C after deposition of NNO, which leads to reduced magnetization of LSMO as opposed to the single layer LSMO, which is annealed at 950 °C (Fig. S5). This reduction in *M*_*S*_ does not affect the damping of the LSMO in the bilayers (Fig. S6c) and the observed damping enhancement is primarily due to spin pumping effect of NNO.

We observe that above *T*_*MIT*_, the overall spin-to-charge conversion efficiency increases with NNO thickness following the reduction of the film’s sheet resistance (Fig. 1e). This happens in systems when Dyakonov-Perels scattering is the dominant mechanism for spin relaxation^52,53^. The values of *θ*_*SHA*_ range from 1 to 3% for T > 200 K, reaching a maximum close to *T*_*MIT*_ and decreasing drastically below this characteristic temperature due to NNO’s insulating nature.

The abrupt reduction of *I*_*C-SP*_ (188% and 76%) and the *θ*_*SHA*_ (81% and 162%) for LSMO(12)/NNO(8) and LSMO(12)/NNO(16) respectively, from their values at *T*_*MIT*_ to their values 20 K below *T*_*MIT*_ can be attributed to the reduction of itinerant electrons in the insulating phase of NNO, which no longer efficiently convert the spin current into charge current. This is a well-known phenomenon to occur in the electron hopping/dirty metal regime^54,55^. Additionally, the metallic NNO exhibits a larger *θ*_*SHA*_ for increased layer thicknesses, indicating a significant bulk contribution. Based on these observations, we infer that the spin scattering mechanism in NNO operates in the dirty metal regime and is dominated by extrinsic bulk contributions. This is consistent with the increased slope for thicker NNO in the apparent spin Hall conductivity (σ_SH_*) vs the longitudinal conductivity for NNO (σ_xx_) plot in the metallic phase in Fig. S11c, where σ_SH_* is calculated by^56^:

4 $${\sigma }_{{SH}}^{*}={\sigma }_{{SH}}\,{T}_{{{\mathrm{int}}}}$$

*σ*_*SH*_ is the spin Hall conductivity given by ${\sigma }_{{SH}}=\,{\theta }_{{SHA}}\,{\sigma }_{{xx}}$^46,56–58^, and *T*_*int*_ is the interfacial spin transparency which is calculated by the following equation^41,59^:

5 $${T}_{{{\mathrm{int}}}}=\frac{{g}_{r}\tanh \left(\frac{{t}_{{NNO}}}{{\lambda }_{{NNO}}}\right)}{{g}_{r}\coth \left(\frac{{t}_{{NNO}}}{{\lambda }_{{NNO}}}\right)+\frac{h}{{2{e}^{2}\rho }_{{NNO}}{\lambda }_{{NNO}}}}$$

*g*_*r*_ is the real part of *g*_*eff*_ which is assumed to be equivalent to *g*_*eff*_^41,59^.

**Fig. 4:**
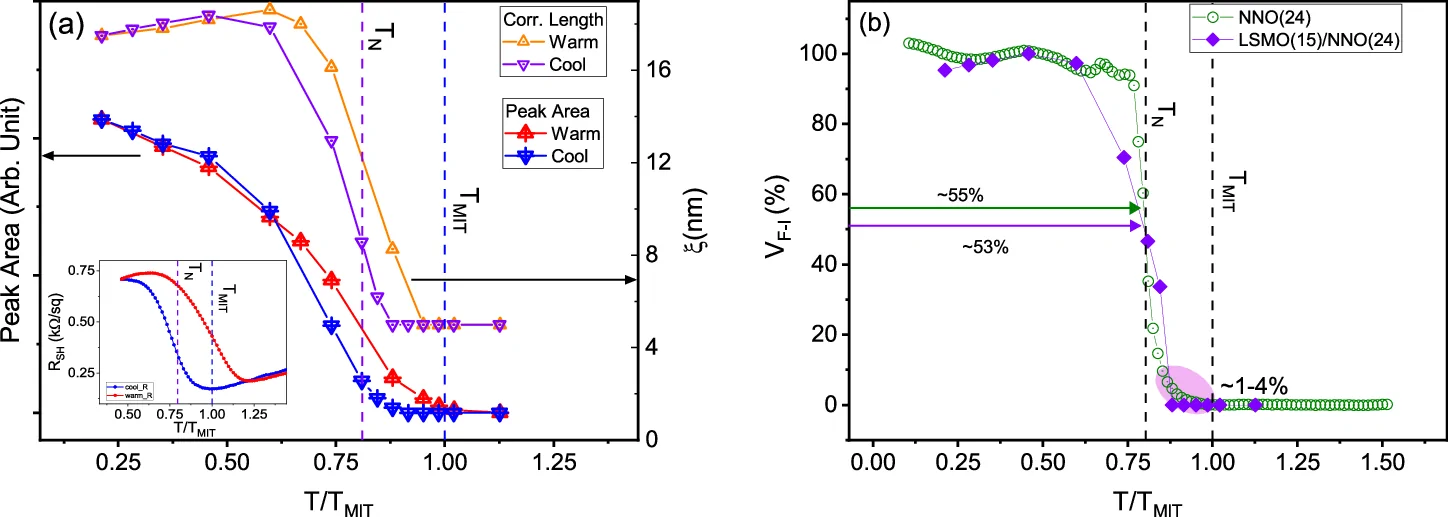
Demonstrating a first-order domain-mediated phase transition in LSMO/NNO. **a** Shows the peak area and correlation length for the NNO’s (¼ ¼ ¼)_pc_ AFM Bragg peak. The inset shows the resistance vs T measurement of the same LSMO(15)/NNO(24) sample. **b** Shows the estimated volume fraction ( ~ 1–4% in the sparse regime) determined from resistance and correlation length measurements. The values for correlation length ratio has been offset to zero above the T_MIT_.

The drastic enhancement of *I*_*C-SP*_ (and the *θ*_*SHA*_) occurs at the onset of the MIT (T~*T*_*MIT*_) in NNO, followed by a subsequent reduction for T < *T*_*MIT*_. At *T*_*MIT*_, it has been observed that there is nucleation of insulating AFM domains of the order of ~20–100 nm^5,11,60^. To directly probe and quantify the insulating AFM domain formation in LSMO/NNO, we turn to resonant X-ray diffraction. We performed resonant soft X-ray scattering (RSXS) measurements while tuned to the Ni *L*_*3*_ edge and studied the temperature evolution of NNO’s (¼ ¼ ¼)_pc_ AFM Bragg peak in an LSMO(15 nm)/NNO(24 nm) bilayer. The NNO layer is thicker here to achieve a better scattering signal. *T*_*N*_ and *T*_*MIT*_ for this sample were determined using the same methods outlined previously. RSXS measurements reveal the appearance of a distinct magnetic peak associated with the insulating AFM phase of NNO (Fig. S13a), which develops near the Néel temperature (confirming that our calculated *T*_*N*_ values are accurate) and exhibits clear hysteresis (Fig. 4a). In agreement with the hysteretic resistance versus temperature curve, the hysteresis in the AFM peak’s temperature dependence is direct evidence of a first order phase transition involving domain nucleation.

To determine the prevalence of insulating AFM domains at *T*_*MIT*_, where we observed the spin pumping and spin Hall angle enhancement, we calculate the volume fraction of such domains (V_F-I_) in the film. From the RSXS data, we use the full width at half maximum (FWHM) of the magnetic peak to extract the correlation length ξ, where $\xi=2/{FWHM}$, of the insulating AFM domains. The correlation length of these domains is found to be ~9 nm at *T*_*N*_ and saturates around 18 nm for T < *T*_*N*_ (Fig. 4a). Note that above *T*_*N*_, the correlation length is an arbitrary value set by the peak fitting constraints. The volume fraction V_F-I_ is estimated by the ratio ξ (T)/ ${\xi }_{{Max}}$. For temperatures where the peaks had poor fit confidence (e.g., *T* > *T*_*N*_), ξ was offset to zero to account for this uncertainty (Fig. S13b). At *T*_*N*_, the volume fraction for insulating antiferromagnetic domains was estimated to be 53% (Fig. 4b), similar to other reports^1,2,11^. However, due to NNO’s weak AFM Bragg peak at *T*_*MIT*_, we cannot estimate V_F-I_ for *T*_*MIT*_ from the RSXS data. Instead, we estimate V_F-I_ using resistance measurements of a single layer NNO(24 nm) film, allowing us to further corroborate the V_F-I_ obtained from RSXS measurements and accurately estimate V_F-I_ at *T*_*MIT*_. Following the method outlined by Catalan et al.^11^ (more details in supp. info. and Fig. S12a, b), we estimate V_F-I_ at *T*_*N*_ to be ~55%. More importantly, we are able to estimate the V_F-I_ at *T*_*MIT*_ to be around ~1–4%. Therefore, the AFM insulating domains are sparse at *T*_*MIT*_ where we see enhanced spin pumping behavior.

## Discussion

NdNiO_3_ has both intrinsic and extrinsic contributions to the spin-to-charge conversion. The extrinsic contribution decreases as the NNO thickness is reduced and moves towards the clean metal limit (Figure S11c). This thickness-dependent scaling reveals that the interfacial contribution to spin-to-charge conversion may be predominantly intrinsic in nature. With this knowledge, we can decouple the intrinsic (interface-driven) and extrinsic (bulk-driven) effects and provide insights into the observed enhancement of *θ*_*SHA*_.

At *T*_*MIT*_, AFM insulating domains ( ~ 1–4% by volume fraction) have begun nucleating within a largely metallic matrix but are still sparse in the NNO. To determine the effect of these sparse domains on the intrinsic SHE component we use the Drude model approximation (${\sigma }_{{xx}}=n{e}^{2}\tau /{m}^{*}$) to calculate the relative variation of spin scattering lifetime (or carrier lifetime) τ for different NNO thicknesses with respect to τ at 300 K i.e., $\frac{\tau }{{\tau }_{300K}}\approx \frac{{\sigma }_{{xx}}}{{\sigma }_{{xx},300K}}$. We obtain the ${\sigma }_{{xx}}$ of NNO by subtracting the measured single layer LSMO conductivity (grown in similar conditions as bilayer LSMO) from the measured conductivity of the LSMO/NNO bilayer. This implicitly incorporates the interfacial effects into NNO’s conductivity.

**Fig. 5:**
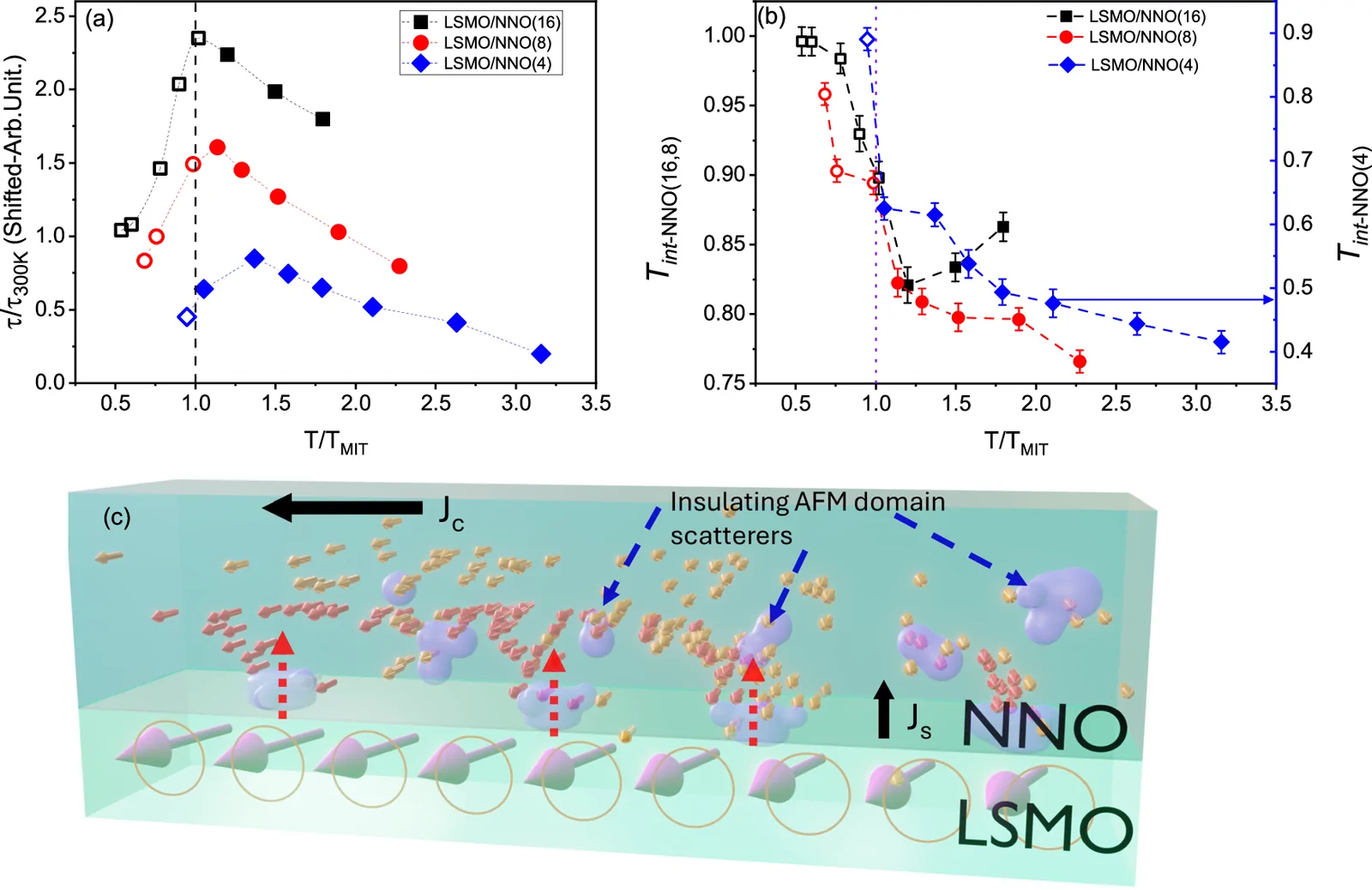
Effect of phase transition on spin transmission. **a** The variation of the relative carrier lifetime for different NNO thicknesses, the data is shifted for clarity. **b** The variation of the interfacial spin transparency at the LSMO/NNO interface, which shows a distinct jump especially for LSMO/NNO(16), as the NNO enters the MIT. The metallic phase data are shown as solid points for both figures, error bars denote SE. **c** A schematic of the enhanced spin transmission due to the cumulative effect of increased scattering (yellow arrows) and spin transparency effects (red dashed arrows).

From Fig. 5a, we observe that the relative τ increases in the metallic phase as we get close to the *T*_*MIT*_ and is reduced in the far insulating phase. For NNO(16) where the bulk extrinsic effect dominates, we do not see any apparent reduction of τ at *T*_*MIT*_. However, for LSMO/NNO(8-4), where the intrinsic contribution is more apparent, we observe a clear reduction of τ at *T*_*MIT*_. This enhancement in *θ*_*SHA*_ due to reduction of carrier lifetime (within a certain limit) is in line with the reported observations for Pt with doped insulating impurities like TiO_2_ or MgO^56,61,62^. The corollary for NNO is that at *T*_*MIT*_, these sparse insulating AFM domains act as “doped” impurities, similar to the doped Pt systems. The combination of this reduced scattering lifetime τ and a high DOS of conduction electrons at *T*_*MIT*_, thanks to the majority metallic matrix at the transition onset, can convert the injected spin current into a large charge current, resulting in a much larger spin-charge conversion. Just below *T*_*MIT*_, the insulating AFM domains quickly take over a significant volume fraction of the NNO films, further decreasing τ and reducing the available itinerant electrons, subsequently causing the large reduction in the *I*_*C-SP*_ and *θ*_*SHA*_. We do not see the enhancement in the NNO(4) sample near its phase transition (*T*_*MIT*_ ~ 95 K) due to reduced extrinsic SHE along with significant dissipation of spin currents within LSMO’s bulk and interface at lower temperatures (*T* < 110 K)^41^.

Apart from the intrinsic contribution, we also observe a clear enhancement in the interfacial spin transparency (*T*_*int*_) (Fig. 5b) at the LSMO/NNO interface (e.g., *T*_*int*_ increases from 82% to 90% for LSMO/NNO(16)) across *T*_*MIT*_. *T*_*int*_ can affect the overall spin current density being pumped into the NM and depends on the electronic structure matching between the FM and NM, along with the degree of spin memory loss at the interface^41,46,48,59^. Previous studies have demonstrated highly transparent interfaces by inserting an AFM oxide layer like NiO between the FM and NM^63–67^, and thus this increase of *T*_*int*_ is likely due to the AFM nature of the domains. An increase in *T*_*int*_ combined with high NNO conductivity at the *T*_*MIT*_, which is most pronounced for thicker NNO films with a dominant bulk contribution (i.e., NNO(16)), enhances the apparent spin Hall conductivity (Fig. S11d). This behavior provides a possible explanation for the observed increase in *θ*_*SHA*_ in the extrinsic regime (θ_SHA_$\,\propto$ ${\sigma }_{{SH}}^{*}$).

Overall, these findings show that intrinsic heterogeneity due to phase coexistence during a first-order phase transition can be used to enhance spin pumping and spin-charge conversion efficiencies. The intrinsic and extrinsic contributions work in tandem to increase the spin-charge conversion. These results clearly highlight the potential advantages offered by complex oxides in a device context; their rich physics and phase transitions enable a continuum of material states and device properties not achievable in metals without external doping or other permanent modifications.

To contextualize our observation of spin pumping enhancement at NNO’s phase transition, we turn to other recent works in this active area of research. Probing the spin dynamics variation to explore spin pumping enhancement during the magnetic/electronic phase transition criticality is a significant area of research from both materials and devices perspective. Materials with second-order paramagnetic to AFM transitions have exhibited spin pumping-related enhancements at *T*_*N*_ when used as spin sink (IrMn^68^) or as spin transport (CoO and NiO^69^) layers. Notably, the mechanism behind these enhancements is distinctly different than that in LSMO/NNO due to the differences between first and second-order phase transitions. Since these second-order transitions do not involve the same domain formation as first-order transitions, the spin pumping increase at *T*_*N*_ seen in these second-order transition systems is instead due to the temperature dependences of the interfacial spin mixing conductance^59^ and the dynamical magnetic susceptibility^60^, which are also maximized at *T*_*N*_. These previous studies showed that this reliable transition-induced enhancement can be used to track the dependence of *T*_*N*_ on film thickness^59,60^, demonstrating that this phenomenon can be useful for materials characterization.

Spin pumping variation around phase transitions has also been studied in materials with first order phase transitions, such as VO_2_^70,71^ and FeRh^72^. However, an enhancement of SHA at the onset of a phase transition has not been previously reported in these first order phase transition systems. For example, in VO_2_, the spin-charge conversion decreases monotonically, with no enhancement at the *T*_*MIT*_; in stark contrast to the present work where the *I*_*C-SP*_ and *θ*_*SHA*_ are enhanced due to formation of sparse insulating domains. In VO_2_-based structures (VO_2_/YIG or VO_2_/Py^70,71^), where VO_2_ undergoes its MIT through filament formation^54,70^, it was observed that the insulating phase of VO_2_ exhibits a sudden lowering of damping^54^ due to reduced density of states (DOS) of conduction electrons, leading to less spin dissipation. VO_2_ also generates larger spin torques in the metallic phase owing to the higher DOS of conduction electrons and thus exhibits a larger *θ*_*SHA*_ as compared to its insulating phase. Unlike the case of VO_2_, where the insulating phase exhibits reduced damping, we find that the AFM insulating phase of NNO demonstrates a higher damping value, demonstrating FM-AFM coupling in this bilayer. Further, the damping of LSMO/NNO does not follow the hysteretic change of the resistivity with temperature (Fig. S6e), suggesting that spin dissipation due to AFM-FM interaction between NNO and LSMO dominates over interband (or “resistivity-like”) electron scattering in NNO. This also distinguishes LSMO/NNO from the FeRh case, in which the damping enhancement has been attributed to lateral spin pumping between macroscopic (on the order of a few microns) AFM and FM domains within the FeRh itself and is dominated by interband electron scattering^72^. The SHA enhancement we observe in LSMO/NNO is instead tied to the increased spin scattering from nucleating insulating AFM domains in the NNO layer along with enhanced spin Hall conductivity driven by increased interfacial spin transparency across the phase transition. Therefore, where VO_2_-based structures exhibit a phase-dependent SHA and FeRh-based structures see an enhancement in damping at *T*_*N*_ due to spin pumping between FM and AFM domains within the FeRh layer, LSMO/NNO remains distinct as an FM/NM structure with SHA enhancement due to phase transition-induced heterogeneity.

The differences between the enhancement in *θ*_*SHA*_ we observe in LSMO/NNO and that seen in other material systems emphasize that the fundamental physics of this perovskite system is essential to the tunability it offers. Although the absolute value of *θ*_*SHA*_ is modest compared to other oxide/oxide or metal/oxide bilayers^41,73–79^, coherently grown LSMO/NNO heterostructures offer a uniquely rich platform for exploring spin transport, spin-charge conversion, and interfacial spin phenomena. Coherently grown bilayers accommodate substrate-induced strain, maintaining an atomically ordered and structurally coherent interface that minimizes interfacial disorder and helps in preserving strong electronic and magnetic coupling. The sharp, defect-free interface (Fig. 1a). promotes direct interaction between two distinct magnetic layers, namely the ferromagnetic LSMO and the antiferromagnetic NNO, exemplified by FM-AFM coupling between these layers. Such connectivity allows external parameters like temperature and magnetic field to modulate the exchange interactions in a fundamentally different way from conventional metallic heterostructures, where lattice-mediated interactions are largely absent.

The demonstrated tunability of spin pumping and spin-charge conversion in oxide heterostructures like LSMO/NNO is a step toward reconfigurable, energy-efficient spintronic and neuromorphic devices that go beyond the capabilities of conventional material systems. Because of the strong interactions in these Pv-TMO systems, external perturbations can selectively modify heterostructures’ properties in desirable ways. For example, methods like Sm doping^79^ have been used to raise NNO’s transition temperature, which can enable room-temperature nickelate-based spintronic devices. Further phase transition engineering can achieve desired spin dynamics and transport properties for device applications. Since spin-to-charge conversion is governed by the intrinsic coupling of spin, orbital, and lattice degrees of freedom in Pv-TMOs and is tunable through epitaxial strain, spin-charge conversion can be efficiently and locally manipulated using optical excitation^80^, voltage stimuli^22–24^, laser-induced thermal effects, or voltage-induced strain (e.g., employing PMN–PT substrates^15,81,82^). The tunable spin conduction in NNO, driven primarily by its metal–insulator transition, makes it a promising candidate for applications like weighted spin-valve elements in multilayer spintronic device architectures. The local tunability of phase-dependent manipulation of spin currents into the FM can offer pathways for dynamic learning in neuromorphic computing. Further, NNO displays enhanced spin-sinking effects with reduced spin-charge dissipation in its insulating AFM phase. With a correlation length of 18 nm or more, AFM-NNO may also enable the realization of magnon-based spin transport in tri-layer high-SOC-metal /NNO/LSMO systems^64,66^ owing to the low damping and robust AFM coupling. With such applications in mind, the tunable spin dynamics observed in LSMO/NNO is clear evidence of the novel multifunctionality offered by Pv-TMOs and motivates further study of strongly correlated phase transition materials in a spin transport and device context.

These findings establish coherently grown perovskite oxide heterostructures as a compelling platform where correlated electron interactions, and first-order phase transitions converge to produce new spin–charge conversion phenomena. This work provides the first experimental demonstration of tunable spin–charge conversion in bilayer manganate-nickelate perovskites. Notably, enhancement in the spin-charge conversion efficiency at the onset of the first-order coupled electronic/magnetic phase transition driven by cumulative effects of intrinsic disorder and increased spin Hall conductivity provides new insights towards exploration of spin transport and dynamics in correlated oxide heterostructures, which is distinct from other such phase transition material systems. The behavior observed in the coherently grown epitaxial LSMO/NNO bilayers, driven by robust interfacial magnetic coupling and mesoscopic domain formation, unveils potential tuning knobs for controllable spin conversion in complex oxides and paving the way for multifunctional, reconfigurable spintronic devices.

## Methods

### Sample growth

A total of 12 nm-thick LSMO films were grown on NdGaO_3_(*110*) (NGO) substrates by pulsed laser deposition. KrF laser with energy of 290 mJ and repetition rate of 4 Hz was used to deposit both LSMO and NNO from their respective 99.99% pure targets. The substrate temperature during deposition of LSMO was kept constant at 950 °C. The films were grown at an O_2_ pressure of 200 mTorr. The films were then cooled down in the deposition chamber at the same pressure till 710 °C after which NNO was deposited in-situ at the same deposition pressure. The films were then annealed at the same temperature for 15 min at 7.6 Torr O_2_ pressure and then cooled down to room temperature at the same pressure. For single-layer LSMO, the film was deposited in the same conditions described above, annealed at 950 °C in 7.6 Torr O_2_ pressure for 15 min and then cooled down to room temperature at the same pressure.

### Electrical, magnetic and FMR characterization

The RT measurement of the samples were performed via the 4 probe Van der Pauw method from 300 K to 50 K at 2 K intervals in the warming and cooling cycles to obtain the sheet resistance. The resistance was measured with 5 averages after each temperature point was stabilized. Temperature-dependent magnetometry was performed using a vibrating sample magnetometer (VSM) by Quantum Design to confirm the ferromagnetic phase of the samples. A field modulated flip chip coplanar waveguide FMR modified to adapt to the Quantum Design cryogenic chamber for temperature variation was used for FMR characterization.

### Device fabrication

For fabricating SP-FMR devices, the films were first cleaned by sonication using 50:50 (V/V) Acetone + IPA solution (10 min) and then patterned into devices of 500 × 10 µm wires via UV lithography and Ar ion milling. Next, 200 nm of SiO_2_ was deposited on the entire substrate, leaving areas for gold contact deposition. Finally, 5 nm Ti and 150 nm Au layers were deposited by evaporation to fabricate ground-signal-ground waveguides and the contacts by lift-off. For measurements, the devices were placed in a cryo-chamber with 4 probes: two for sending RF currents and 50 Ohm termination; and two for measuring the resulting voltage. An external field perpendicular to the RF current was swept, and the resulting voltage (due to spin rectification effects) was recorded by a nano-voltmeter.

To Note: For transfer to different measurement chambers, care was taken so as to not allow any contamination on the surface of the bilayers and single-layer films. The samples were transferred to different measurement set-ups in double vacuum-sealed bags with desiccant material to minimize exposure to air. Except during lithography, all other measurements were taken in vacuum conditions.

### STEM characterization

Samples were prepared using the in-situ lift-out method in the Helios G5 Dual Beam instrument operating at 30 keV. A final clean-up at 5 keV was performed to remove part of the amorphous material on the lamella. Samples were observed in a double aberration-corrected JEOL ARM 200 F cold FEG microscope operating at 200 keV. The STEM High Angle Annular Dark-Field (HAADF) imaging was done with a semi-convergence angle of 21 mrad and a current of 60 pA resulting in a probe size of approximately 80 pm. The Electron Energy Loss Spectroscopy (EELS) analysis was performed using the GIF Continuum spectrometer and the K3 direct detection camera in Dual EELS counting mode using a dwell time of 50 ms (5 ms and 45 ms for the low loss and core loss part, respectively). The energy alignment was performed using the zero-loss peak. The acquired core loss EELS spectra were denoised using the Principal Component Analysis method (PCA), keeping the first 20 components.

### X-ray scattering measurements

Resonant soft X-ray scattering (RSXS) measurements were conducted at beamline 29-ID of the Advanced Photon Source using the RSXS end station. Scattering measurements used a Kappa diffractometer to investigate the (¼ ¼ ¼)_pc_ antiferromagnetic peak of the NNO layer while tuned to the Ni L_3_ edge at ~853 eV. θ-2θ scans were taken at temperature steps between 30 K and 200 K for warming and cooling legs to determine the temperature dependence of the AFM order. Absorption measurements were taken using fluorescence yield with the detector moved out of the Bragg condition.

## Supplementary information

Supplementary Information

Transparent Peer Review file

## Source data

Source Data

## Data Availability

The data that support the findings of this study are provided in the Supplementary Information/Source Data file. Source data are provided with this paper.
