# Supplementary material for: Enhanced spin-to-charge conversion in epitaxial La0.67Sr0.33MnO3/NdNiO3 bilayers at the nickelate metal-insulator phase transition
Source: Nat Commun. 2026 Aug 6;17:9461. doi: 10.1038/s41467-026-75812-0 (PMC13542201; doi:10.1038/s41467-026-75812-0)
Supplement: Supplementary file 1 [file 41467_2026_75812_MOESM1_ESM.pdf]

Supplementary information for:

Enhanced spin-to-charge conversion in epitaxial $La_{0.67}Sr_{0.33}MnO_3/NdNiO_3$ bilayers at the nickelate metal-insulator phase transition

Biswajit Sahoo[†], Sarmistha Das[†], Akilan K, Katherine Matthews, Melissa Yactayo, Robin Glefke, Xiaoke Li, Hao Zheng, Sebastien Petit-Watelot, Juan-Carlos Rojas-Sánchez, Alexandre Pofelski, Yimei Zhu, Alex Frano*, and Eric E Fullerton*.

Contents

Sample Fabrication: 1
X-Ray Diffraction: 1
STEM Characterization: 2
Electrical Characterization: 3
Magnetometry measurements: 4
Ferromagnetic Resonance: 6
Device fabrication: 11
Volume Fraction Determination: 12
Resonant X-Ray Scattering and Absorption Spectroscopy: 13
Reciprocal Space Mapping: 14

Sample Fabrication:

12-nm-thick LSMO films were grown on $NdGaO_3$(*110*) (NGO) substrates by pulsed laser deposition. The substrate temperature during deposition was kept constant at 950^0C. The films were grown at an O_2 pressure of 200 mTorr. The films were then cooled down at the same pressure till 710^0C after which NNO was deposited at the same deposition pressure. The films were then annealed at the same temperature for 15 minutes at 7.6 Torr O_2 pressure and then cooled down to room temperature at the same pressure.

X-Ray Diffraction:

The crystallinity of LSMO was determined via X-Ray Diffraction (XRD) using Rigaku Smartlab systems which employs Cu-Kα X-rays of wavelength 1.54 Å. S1(a) shows the full XRD scan for LSMO(12) and LSMO(12)/NNO(16) samples. S1(b) shows the *220* peaks for LSMO(12)/NNO(4-16) samples showing an increase in strain with thinner NNO.

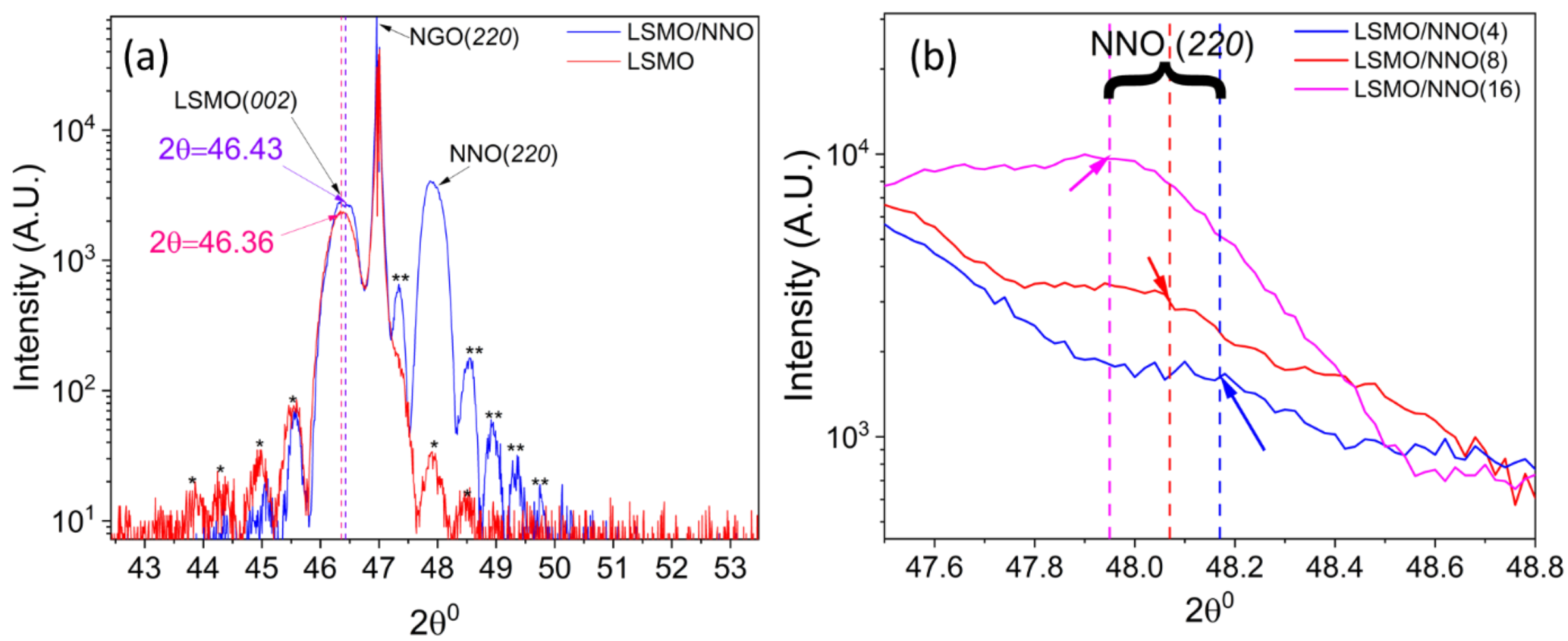

Figure S1(a) XRD of LSMO(12) and LSMO(12)/NNO(16) bilayers showing the reduction of compressive strain of LSMO due to addition of NNO. (b) the NNO(220) peak for LSMO(12)/NNO(4-16) showing an increase in the tensile strain for thinner NNO.

STEM Characterization:

Samples were prepared using the in-situ lift-out method in the Helios G5 Dual Beam instrument operating at 30 keV. A final clean-up at 5 keV was performed to remove part of the amorphous material on the lamella. Samples were observed in a double aberration corrected JEOL ARM 200F cold FEG microscope operating at 200 keV. The STEM High Angle Annular Dark-Field (HAADF) imaging was done with a semi-convergence angle of 21 mrad and a current of 60 pA resulting in a probe size of approximately 80 pm.

The Electron Energy Loss Spectroscopy (EELS) analysis was performed using the GIF Continuum spectrometer and the K3 direct detection camera in Dual EELS counting mode using a dwell time of 50 ms (5 ms and 45 ms for the low loss and core loss part, respectively). The energy alignment was performed using the zero-loss peak. The acquired core loss EELS spectra were denoised using the Principal Component Analysis method (PCA), keeping the first 20 components. Figures S2 (b)-(g) shows the EELS spectra of LSMO(17)/NNO(24) sample showing the chemically sharp LSMO/NNO interface. Figure S2(h) shows a representative example of the proper chemical composition of LSMO in the bilayer La (Mn) concentration of ~14% (20%) as opposed to calculated percentage of 15%(20%).

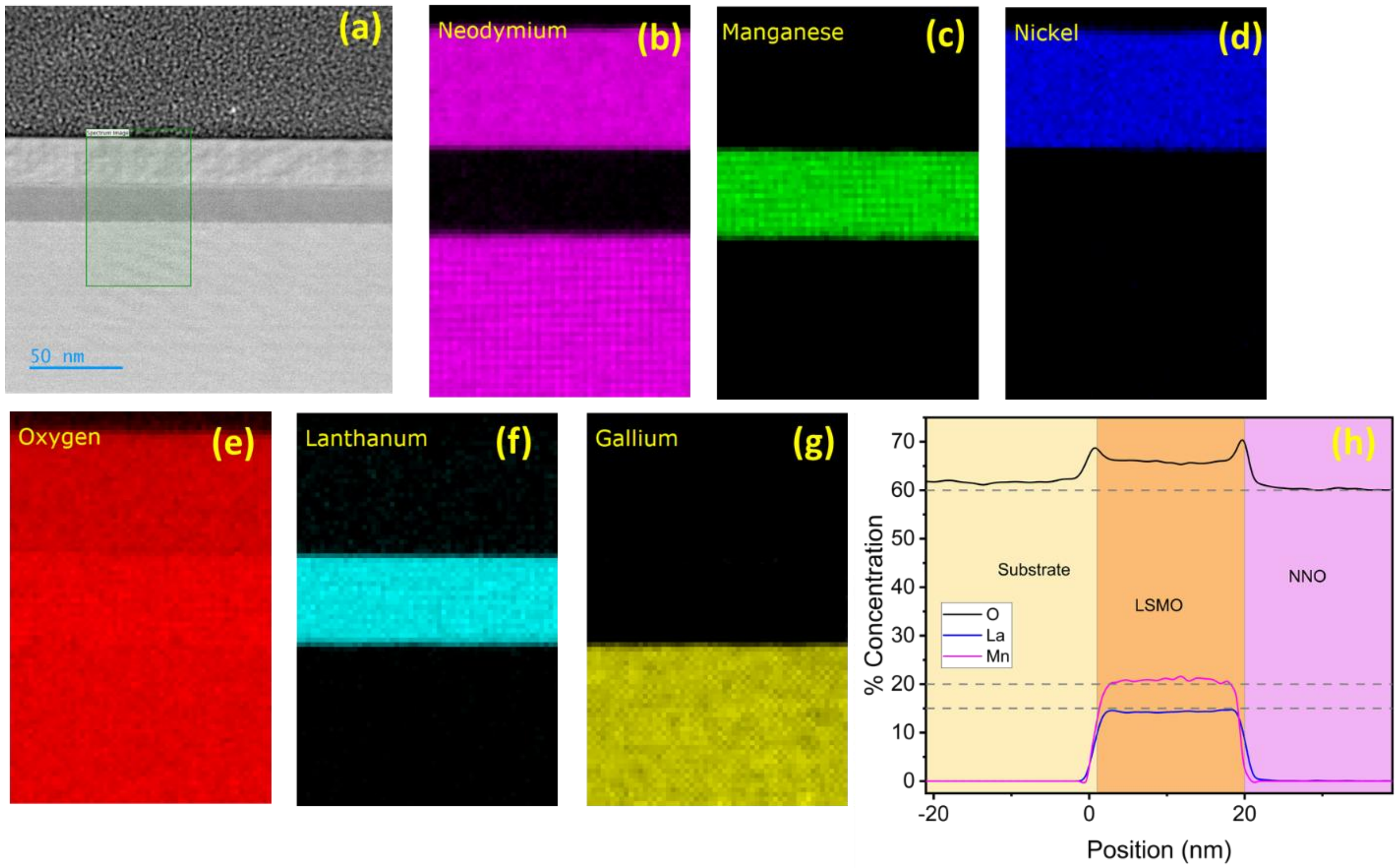

Figure S2 TEM EELS spectra showing distribution of various elements in LSMO/NNO bilayer(b)-(g). (h) shows the elemental concentration of La, O and Mn demonstrating proper chemical composition of LSMO in LSMO/NNO bilayer.

Electrical Characterization:

The RT measurement of the samples were performed via the 4 probe Van der Pauw method from 300K to 50K at 2K intervals in the warming and cooling cycles to obtain the sheet resistance. The resistance was measured with 5 averages after each temperature point was stabilized.

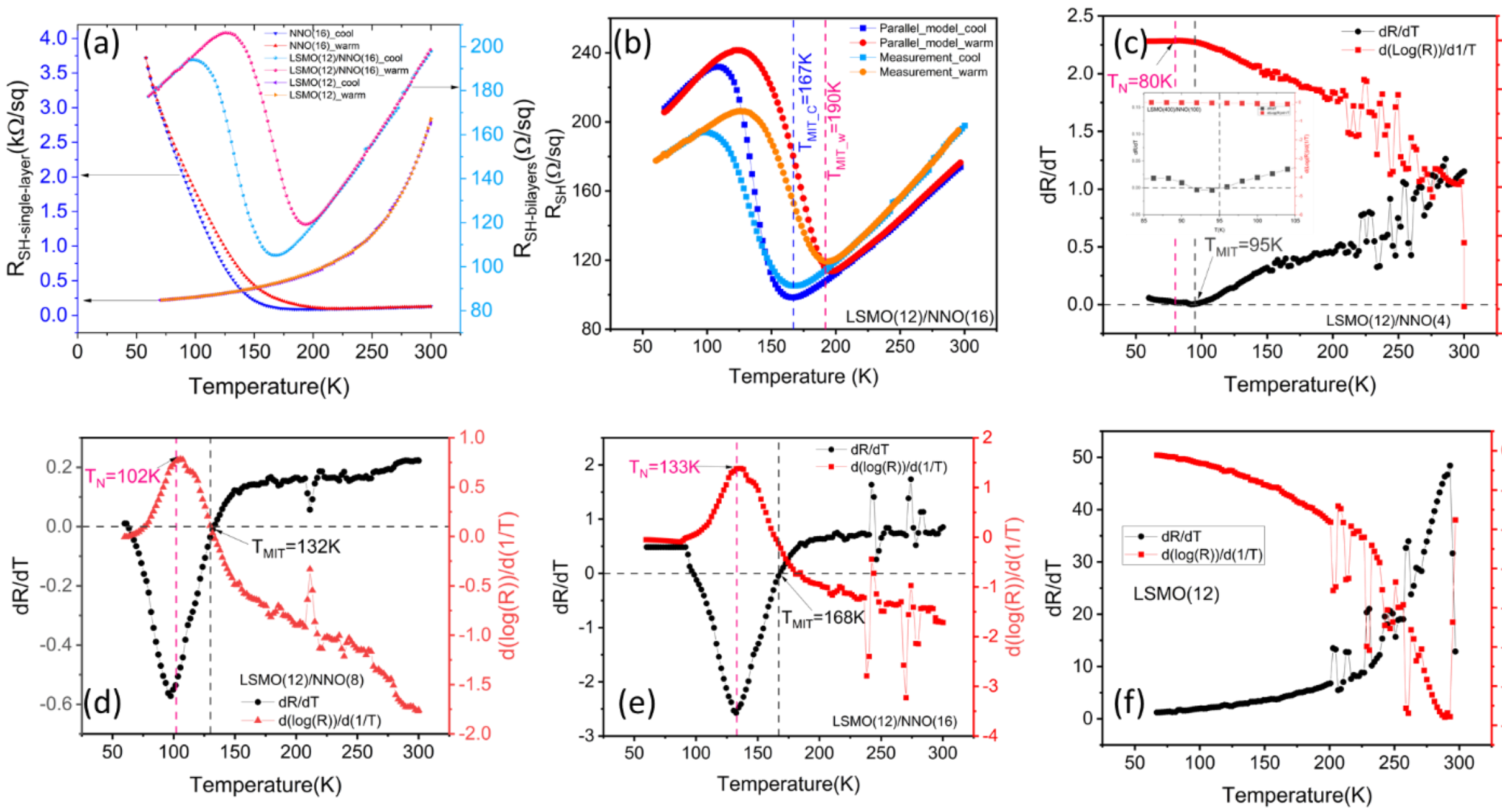

Figure S3 (a) shows the R vs T for LSMO (12), NNO (16) and LSMO (12)/NNO (16). (b) shows the comparison between vs T of the measured and parallel resistor model ($1/R_{LSMO/NNO}=1/R_{LSMO}+1/R_{NNO}$) of LSMO (12)/NNO (16). (c)-(f) show the dR/dT and the d(log(R))/d(1/T) vs T showing the Neel and MIT temperature for LSMO (12)/NNO (4-16). Inset of Fig (c) shows the zoomed in point of the zero crossing. LSMO (12) does not show any such transition.

Magnetometry measurements:

Temperature dependent magnetometry was performed using a vibrating sample magnetometer (VSM) by Quantum Design to confirm the ferromagnetic phase of the samples. FigureS4 shows some representative M vs H plots for NGO (*110*)/LSMO along the <001> and <1-10> orientations of the NGO substrate. Figure S5 shows the M_s obtained from VSM for the LSMO (12) and LSMO (12)/NNO (8-16) samples showing an increase in the T_C for the bilayer samples, possibly due to induction of strain on LSMO.

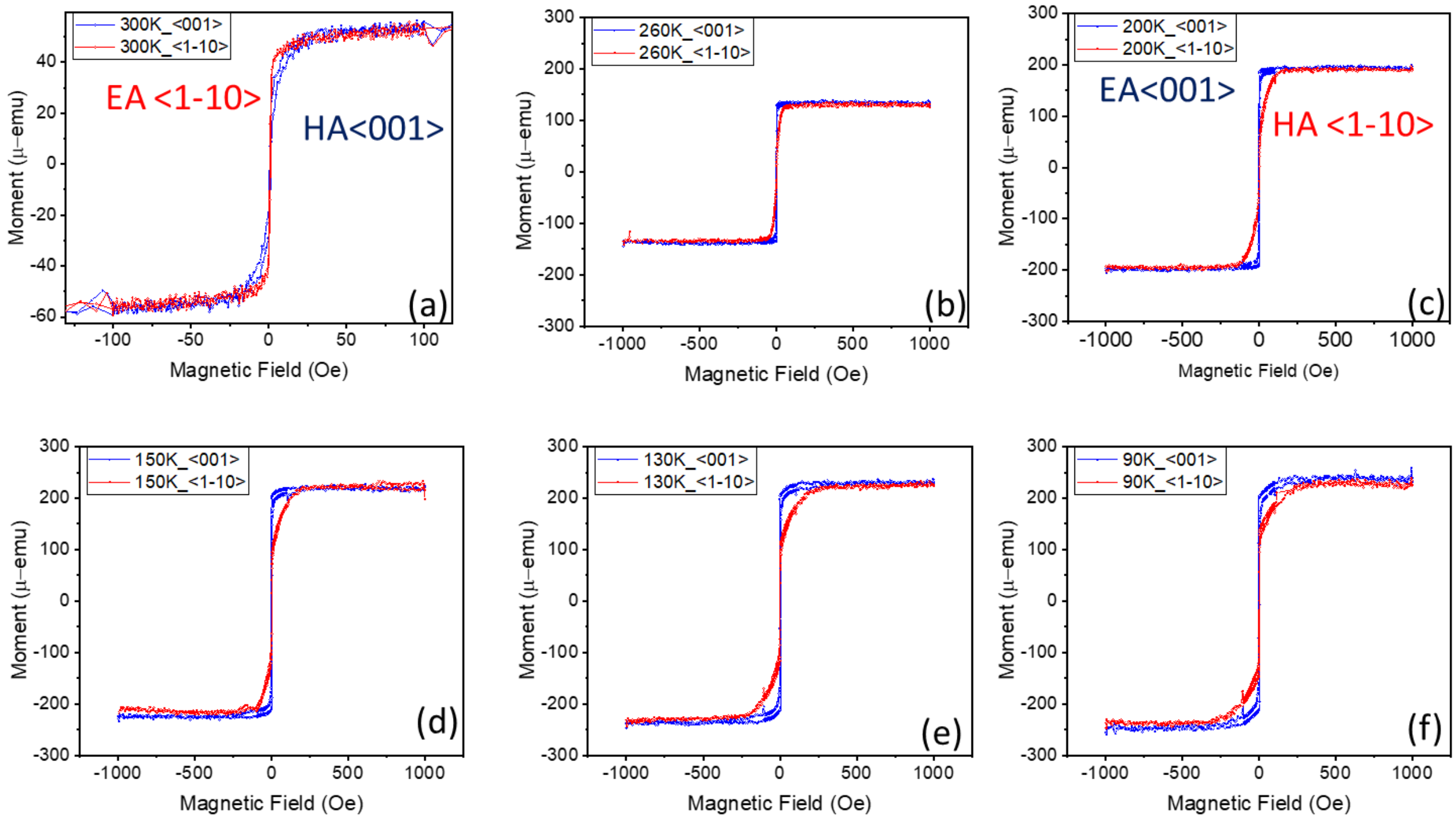

Figure S4 MH loops for NGO (110)/LSMO (12) at different temperatures in the <001> and <1-10> direction of NGO substrate.

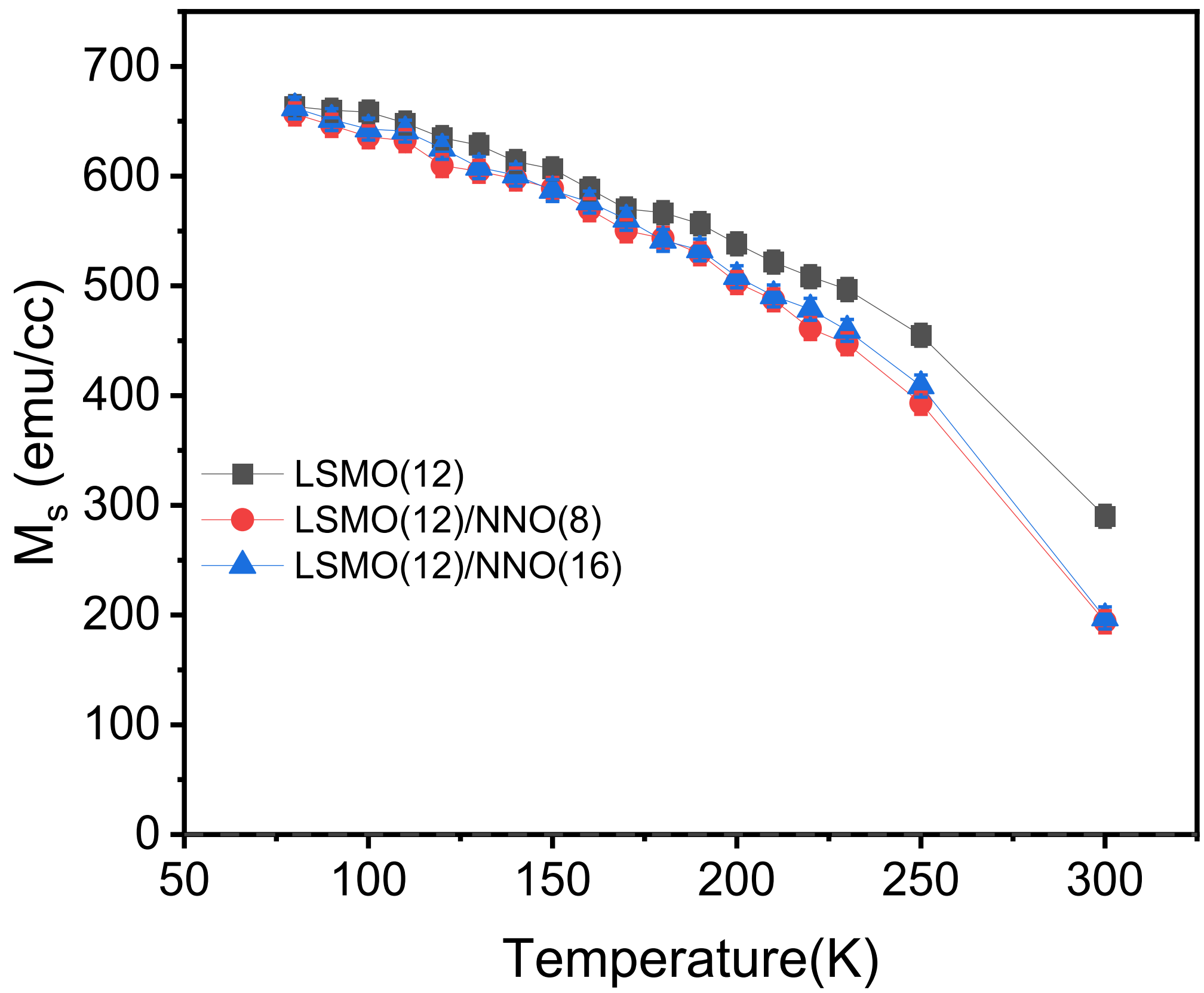

Figure S5 Ms vs T for LSMO (12) and LSMO (12)/NNO (8-16)

Ferromagnetic Resonance:

We employ a field modulated flip chip coplanar waveguide FMR modified to adapt to a cryogenic chamber for temperature variation. We measure the spectra at different temperatures and subsequently evaluate the various magnetic parameters, more importantly, the Gilbert damping parameter as it provides direct insight into the interaction of magnetic moments of LSMO with NNO via dissipation of angular momentum from LSMO. The representative FMR spectra at 8GHz taken at various temperatures for LSMO (12) and LSMO (12)/NNO (16) are shown in Figure S6(a)-(b). By just qualitatively observing the lineshape of the spectra, we immediately see multiple modes at 300K, which is attributed to the lower exchange interaction strength of LSMO, as we are close to the paramagnetic phase transition temperature (≈360K)[1]. At lower temperatures, the signal strength increases, and the modes coalesce into a single stronger mode due to the stronger exchange coupling of the LSMO spins[1]. Similar observation has been seen in other systems involving LSMO ([2,3]). These spectra are fit to the following form containing a symmetric (S_1) and anti-symmetric (A_1) Lorentzian to extract the resonant field (H_{res}) and the linewidth (ΔH):

$$\frac{dP}{dH} = off + mH + S_1 \frac{\Delta H^2 - 4(H - H_{res})^2}{(4(H - H_{res})^2 + \Delta H^2)^2} - 4A_1 \frac{\Delta H(H - H_{res})}{(4(H - H_{res})^2 + \Delta H^2)^2} \tag{1}$$

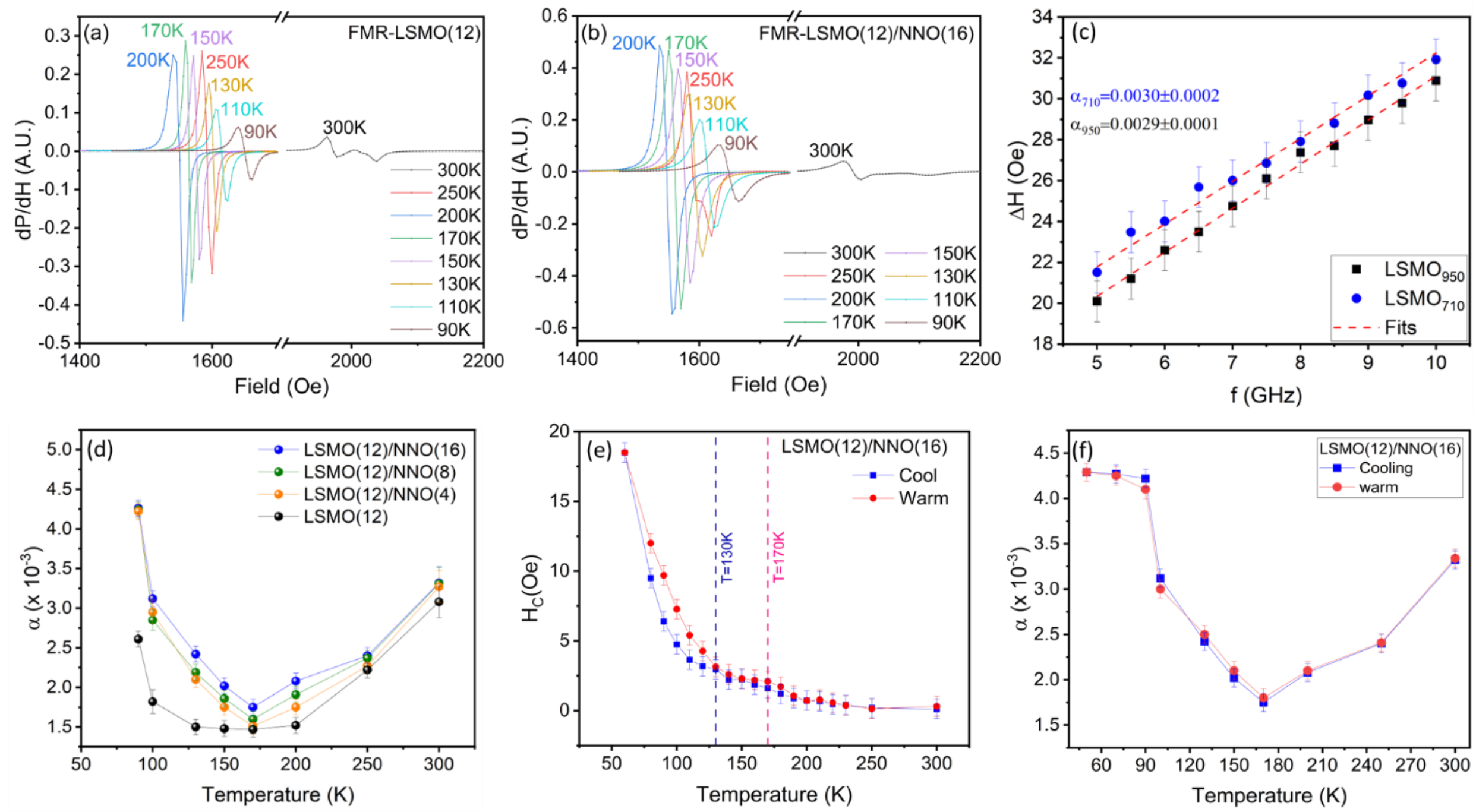

Figure S6 (a) and (b) show the FMR spectra for LSMO (12) and LSMO (12)/NNO (16) respectively. (c) shows the linewidth vs frequency (measured at 300K) of two single layer LSMO samples grown at 950 °C but annealed at 950 °C ($LSMO_{950}$) and 710 °C ($LSMO_{710}$) showing statistically similar damping value. The compilation of the Gilbert damping variation with temperature is shown in (d). (e) shows the variation of coercivity with temperature in LSMO (12)/NNO (16) sample showing the onset of FM ordering. (f) shows the variation of damping for LSMO (12)/NNO (16) in the warming and cooling cycle showing the absence of hysteresis in the Gilbert damping.

The variations of H_{res} vs frequency (f) are fit to the Kittel equation[4]:

$$f = \frac{\gamma}{2\pi}\sqrt{(H_k + H_{res})(H_k + H_{res} + 4\pi M_{eff})} \tag{2}$$

Here ϒ is the gyromagnetic ratio, H_k is the in-plane uniaxial anisotropy field and M_{eff} is the effective magnetization. Some representative fits for LSMO (12) in the <100> and <1-10> direction are shown in Fig S7(a)-(b). The H_k values obtained by fits from Eq (2) reveal the increasingly negative values when measured along the <1-10> direction (Fig. S7(d)), indicating that the observed trend of H_{Res} with temperature is driven by the anisotropy of LSMO (LSMO is slightly more strained in the <001> axis of NGO as opposed to <1-10>, subsequently inducing an in-plane anisotropy in LSMO creating a hard and an easy axis along the <001> and the <1-10> directions of NGO respectively, at room temperature [5]). To maintain consistency, measurements for all the samples have been performed along the <1-10> direction of NGO.

The variation of the linewidth (ΔH) with frequency provides us with the gilbert damping α of the system using the following equation:

$$\Delta H = \Delta H_0 + \frac{4\pi\alpha}{\gamma} f \tag{3}$$

Here ΔH_0 is the inhomogeneous linewidth broadening. The plots and fits to Eq 3 for LSMO (12)/NNO (4-16) are shown in Fig. S7. The fits follow a linear trend, indicating minimal contribution of effects such as two-magnon scattering and mosaicity broadening, which can introduce non-linearities in ΔH vs f

behavior. Lower value of α indicates lesser dissipation of the spin angular momentum (either into the lattice, or due to spin orbit effects), leading to better energy efficiency in spintronic devices.

At room temperature, the LSMO has a low damping of around 0.0030. The bilayer samples show slightly increased damping values of around 0.0033 (Fig. 3c). The damping decreases, with a minima at 170K for all the samples, albeit with an increased damping value for the bilayer samples, which may be attributed to the additional spin dissipation in the NNO layer due to spin pumping. For T<170K the damping increases for all the samples, with a more prominent increase for the bilayers, as opposed to LSMO. Similar behavior has also been observed in NGO/LSMO/Pt where the Pt was deposited *ex-situ*[3], which was attributed to spin dissipation due to increased interaction between different Mn valence states in the bulk and at the top and bottom interface of NGO/LSMO/Pt. In the present case since the deposition is *in-situ*, we speculate that LSMO may have induced additional moment in the adjacent Ni ions in NNO due to magnetic proximity effects (MPE). The increased interaction between the Mn and Ni spins at lower temperatures may have caused the damping enhancement resulting in the similar trend of damping increase for T<170K in the bilayers. We attempted to quantify this induced moment, via M_s vs T measurements for samples LSMO (12)/NNO (8-16). The bilayers exhibited a slightly lower Curie temperature (T_C) as opposed to LSMO (Fig S5), which may be attributed to the tensile strain [6] on LSMO caused by the overlying NNO layer, as exemplified by the LSMO (*002*) peaks of LSMO (12) and LSMO (12)/NNO (16) in Fig S1(a). However, we could not see any enhancement in M_s due to MPE. This may indicate that the MPE is too weak to be observed via simple magnetometry, and thus we rely on literature[7,8] to explain this trend in damping due to MPE. In the present case, it is extremely difficult to disentangle the effect of MPE and spin pumping induced damping enhancement, and thus we assume that these two effects work in synergy. The AFM related damping enhancement contribution in the LSMO/NNO bilayers, however, is probably being smeared by the possible MPE + spin pumping at LSMO/NNO interface.

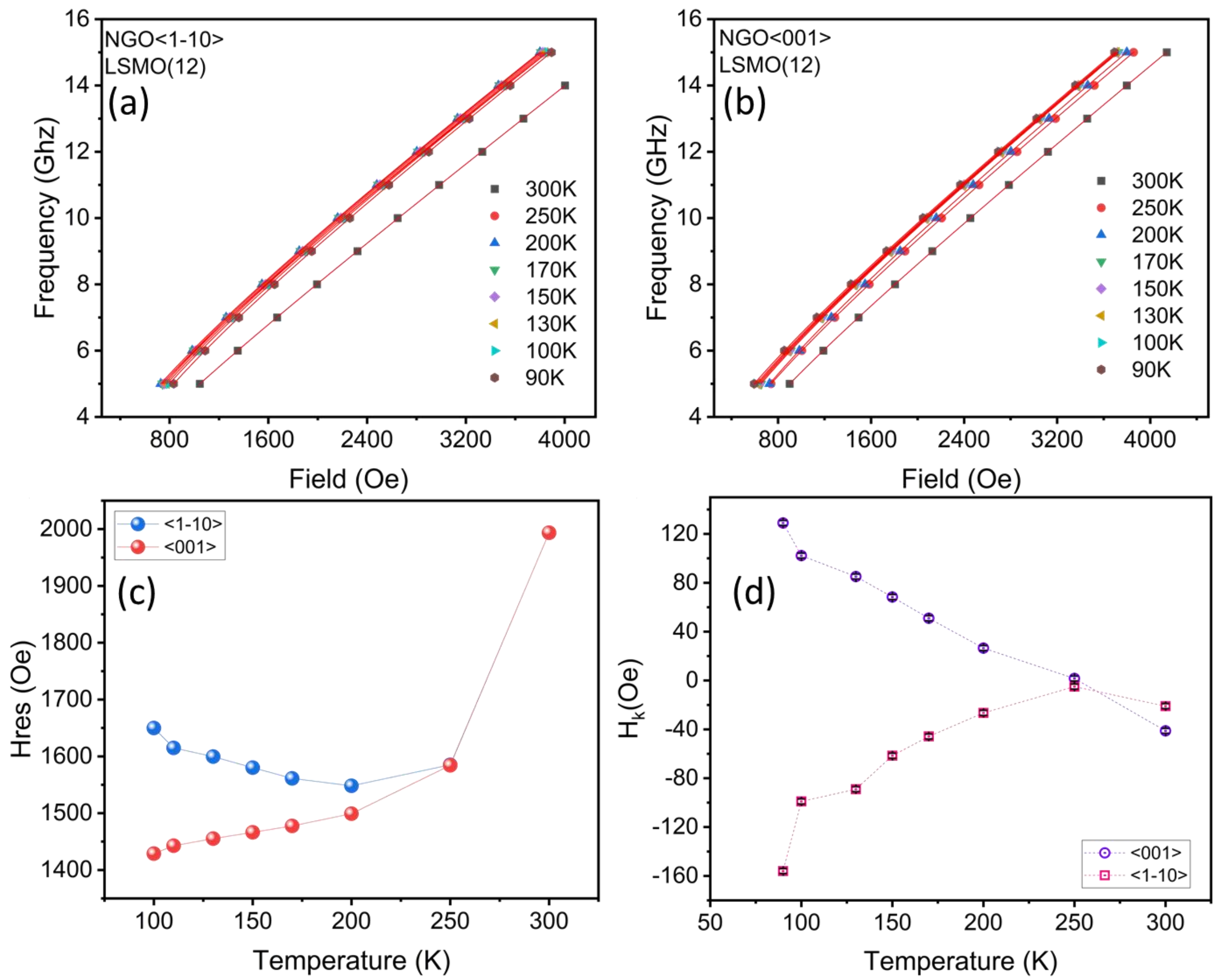

Figure S7 (a)-(b) Kittel fits for LSMO (12) in the <1-10> and <001> NGO directions. (c) shows the H_{res} vs T at 8GHz. (d) shows the variation of H_K obtained from Kittel fitting of (a) and (b).

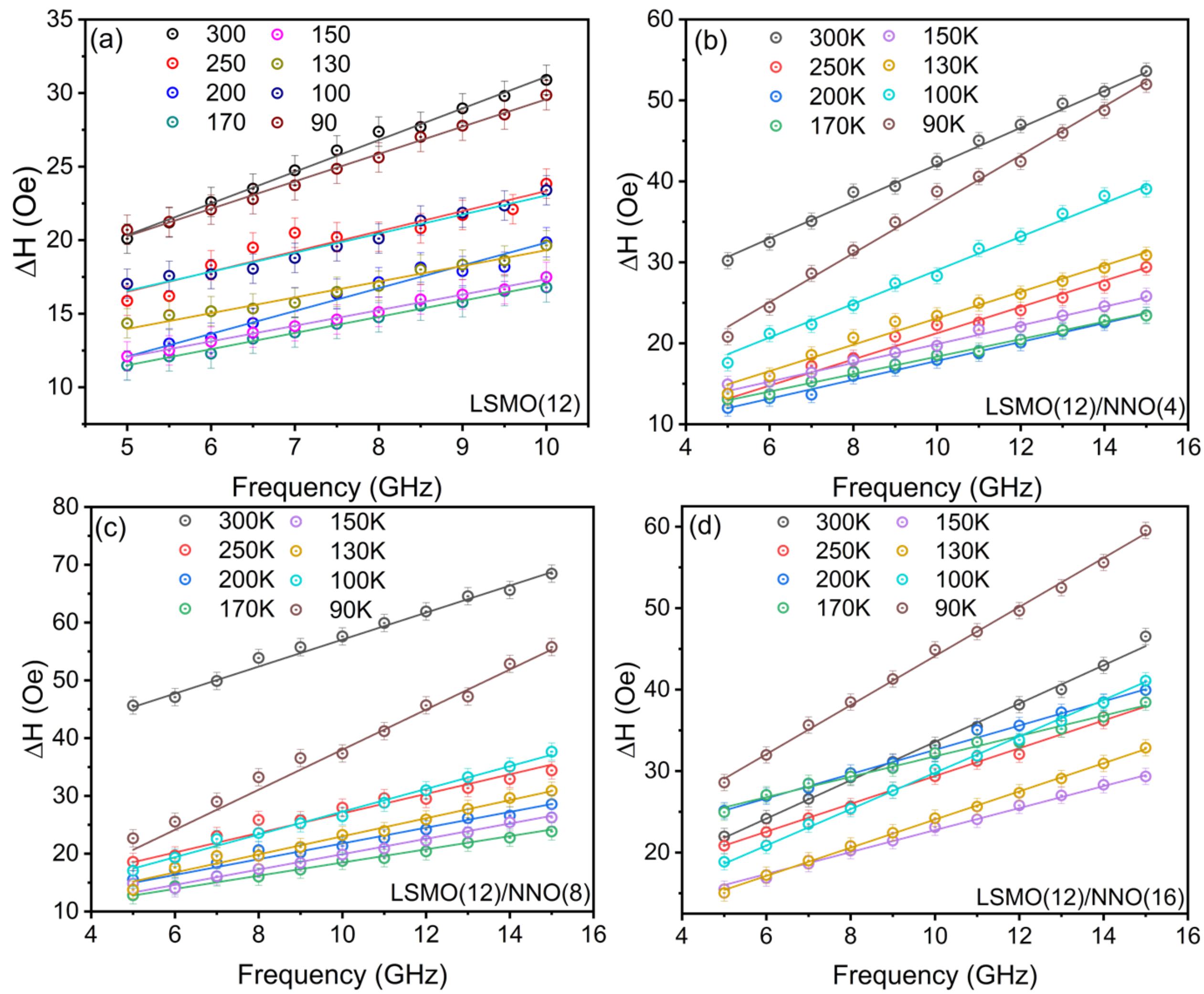

Figure S8 Damping fits for LSMO (12)/NNO (0-16)

To obtain the spin diffusion length ($\lambda_{SF\text{-}NNO}$), the variation in damping is fit to the following equation[9]:

$$\alpha = \alpha_{LSMO} + \frac{g\mu_B g_{Pd}^{\uparrow\downarrow}}{4\pi M_s t_{LSMO}}\left(1 - e^{-\frac{2t_{NNO}}{\lambda_{SF-NNO}}}\right) \tag{4}$$

Furthermore, to contrast with the spin diffusion length obtained by fitting of the $I_{C\text{-}SP}$ variation, the following function was used[10]:

$$I_{C-SP} = A * \lambda_{SF-NNO} \tanh\left(\frac{t_{NNO}}{2\lambda_{SF-NNO}}\right) \tag{5}$$

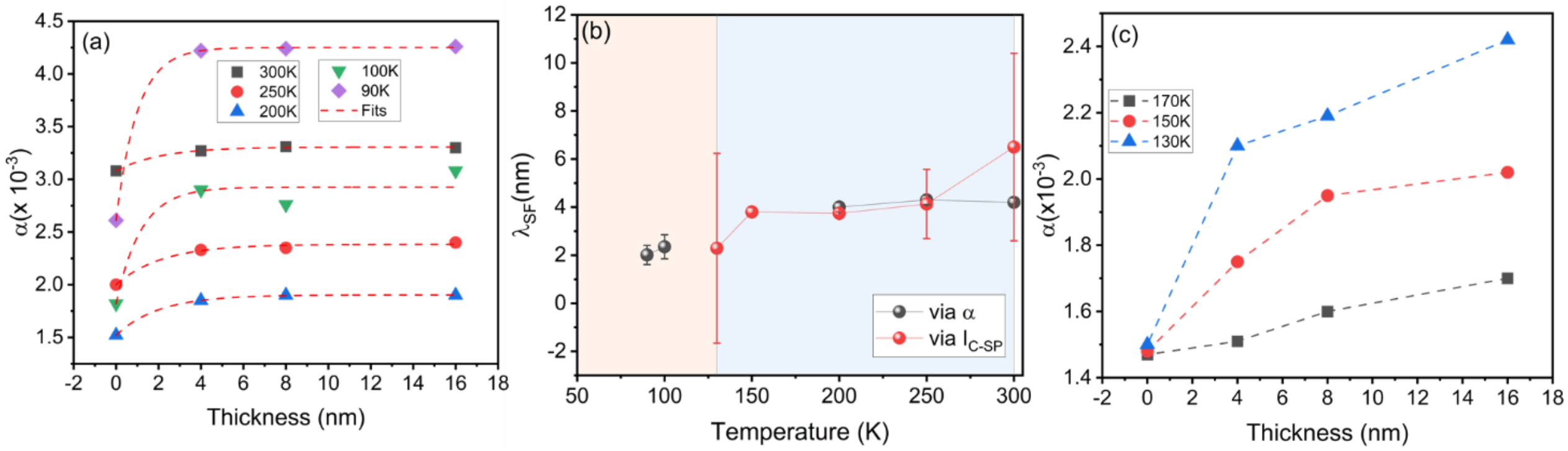

Figure S9 Estimation of λ_{SF} from damping variation (b) by the fits from Eq. 4 (a) (black spheres). Comparative values obtained by fitting the $I_{C\text{-}SP}$ to a tanh (Eq.5) function is shown in red spheres. Both cases show good agreement in values wherever fitting was possible. (c) shows the variation of damping for LSMO (12)/NNO (0-16). from 170-150K for which the fitting did not work, possibly due to enhanced interaction of LSMO with the AFM domains of NNO in LSMO (12)/NNO (16).

Device fabrication:

For Fabricating SP-FMR devices, the film was patterned into devices of 500 x 10 µm wires via UV lithography and Ar ion etching. Next, 200 nm of SiO_2 was deposited on the entire substrate, leaving areas for gold contact deposition. Finally, 5-nm Ti and 150-nm Au layers were deposited by evaporation to fabricate ground-signal-ground waveguides and the contacts by lift-off. For measurements, the devices were placed in a cryo-chamber with 4 probes: two for sending RF currents and two for measuring the resulting voltage. An external field perpendicular to the RF current was swept, and the resulting voltage (due to spin rectification effects) was recorded by a nano-voltmeter.

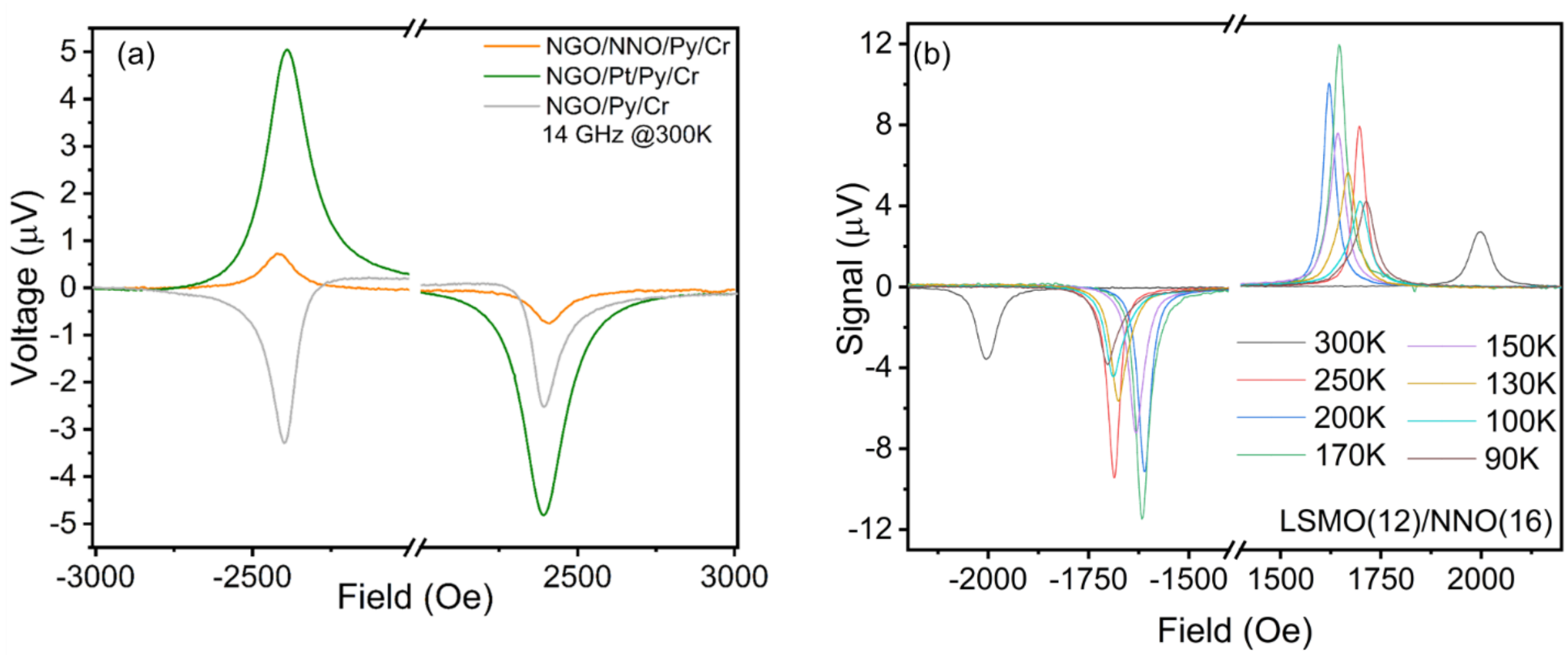

Figure S10 SP-FMR spectra of (a) NNO (23)/Py (7)/Cr (3), Pt (5)/Py (7)/Cr (3) and (b) LSMO (12)/NNO (16) for all the measured temperatures.

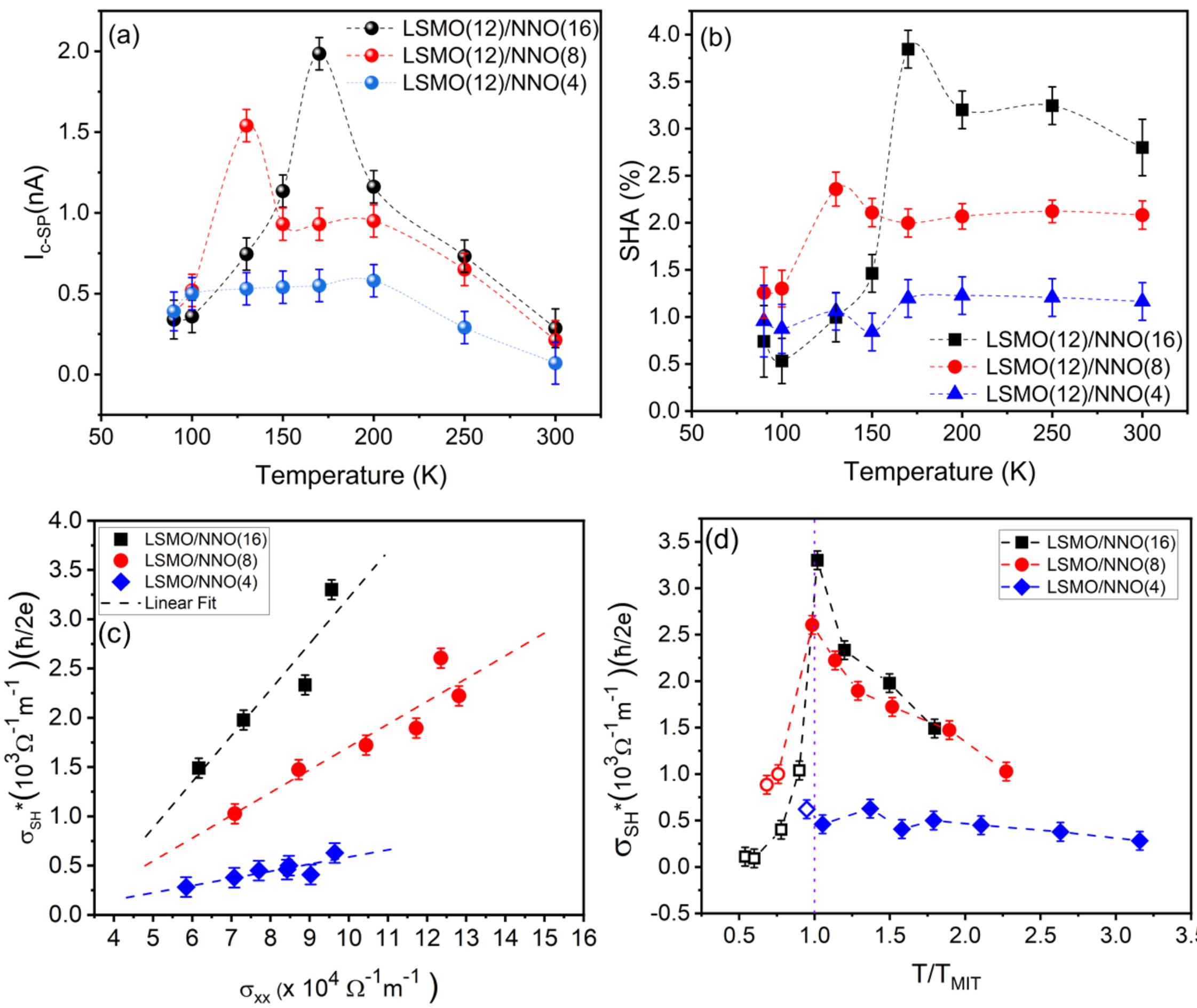

Figure S11 Variation of SHA (a), $I_{C\text{-}SP}$ (b) with temperature. Dotted lines for (a) and (b) are the guide to the eye, c) shows the variation of the effective spin hall conductivity with the longitudinal resistivity showing increased contribution of extrinsic SHE (the slope, dashed lines) with increasing thickness of NNO. (d) Variation of the apparent spin Hall conductivity with temperature for the three samples indicating the extrinsic SHE contribution to the enhancement.

Volume Fraction Determination:

To evaluate the volume fraction of the insulating domains at the phase transition, we approximate the random distribution of the metallic and insulating bonds by a homogeneous lattice where every bond has the same conductance G_e. In the particular case of spherical particles and in the framework of the symmetrical effective medium theory of Bruggeman[11], this conductance is given by[12,13]:

$$\frac{(1-V_s)(G_e-g_m)}{g_m+(D-1)G_e}+\frac{(V_s)(G_e-g_s)}{g_s+(D-1)G_e}=0 \quad (6)$$

Here g_s (g_m)is the conductance of the insulating (metallic) phase. V_s is the volume fraction of the insulating domains. D is the dimensionality constant, which is 3 for 24 nm NNO films[13]. G_e is the measured conductance (1/R) of the film. For evaluating the V_s, one requires evaluation of both g_s and g_m. Evaluation of g_m is straight-forward: we take a linear fit of the measured resistance well above the T_{MIT} (170K in this case). The evaluation of g_s is more involved and requires one to account for variable range hopping and thermally activated effects.

g_s is fit by the following function (for T<120K) as outlined in ref 12, where *A*, *B*, *C*, and *D* are fitting parameters and *T* is the temperature:

$$g_s = Ae^{-B/T^{\frac{1}{4}}} + Ce^{-\frac{D}{T}} \tag{7}$$

The result of the fit as well as the variation of V_s with temperature is shown in figures S12 (a) and (b).

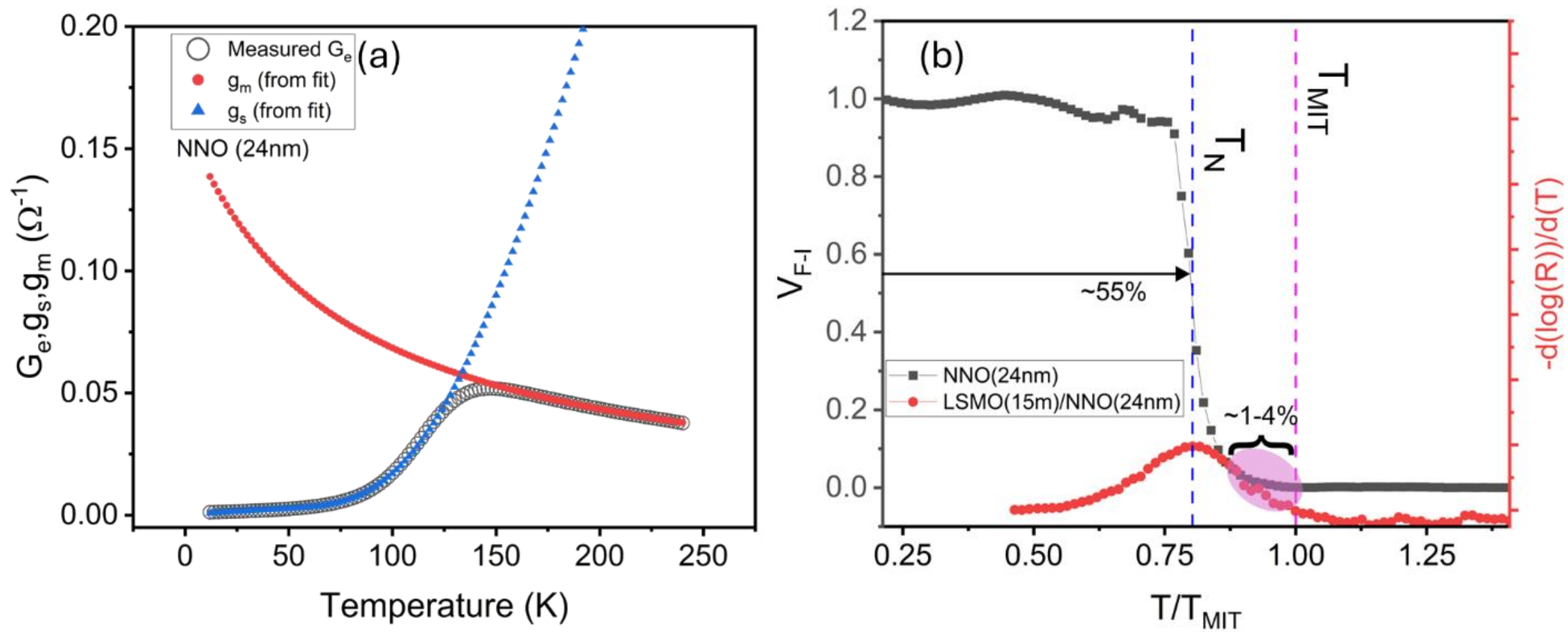

Figure S12 Determination of volume fraction at the onset of phase transition: (a) shows the comparison of the fit-values of conductance of the insulating (blue triangles) and metallic (red circles) superimposed on the measured conductance of $NdNiO_3$ (24nm). (b) shows the evaluation of insulating volume fraction (V_{F-I}) with temperature. At the criticality of the percolation limit (peak of the -d(log(R))/dT, the insulating domains average around 55%. Around T_{MIT} the fraction averages around 1-4% elucidating sparseness of the insulating domains.

Resonant X-Ray Scattering and Absorption Spectroscopy:

Resonant soft X-ray scattering (RSXS) and X-ray absorption spectroscopy (XAS) measurements were performed at the RSXS end-station of beamline 29-ID at the Advanced Photon Source. The antiferromagnetic order of NNO was studied in an NGO (110)/LSMO (15 nm)/NNO (24 nm) heterostructure by tuning to the Ni L_3 edge (determined from XAS scans) and measuring NNO's (¼ ¼ ¼) magnetic peak as a function of temperature for warming and cooling legs.

Scattering measurements were performed with the beamline's kappa diffractometer in a horizontal scattering geometry. Alignment to the AFM peak was performed at each temperature step, and peak measurements consisted of θ-2θ scans and rocking curve θ scans. After converting 2θ values to q using

$$q = \frac{4\pi \sin(\frac{2\theta}{2})}{\lambda} \tag{8}$$

where λ is the X-ray wavelength, the magnetic peak at each temperature step was then fit with a Lorentzian curve in Python, and the peak area and FWHM were extracted from the fitting parameters. Sample θ-2θ scans and corresponding Lorentzian fits for three different temperatures on the cooling leg

are shown in Fig S13 (b). The correlation length λ was then estimated from the FWHM using the following equation:

$$\lambda = \frac{2}{FWHM_q} \tag{9}$$

Absorption measurements were performed using fluorescence yield. XAS scans around the Ni $L_{2,3}$ edges at three different temperatures are shown in Fig S13 (a). The peak around 851 eV corresponds to the La M_4 edge for the LSMO layer, but the higher energy features are associated with the Ni L edges. Of particular interest are the features in the Ni L_3 edge. As indicated by the arrows in Fig S13 (a), the Ni L_3 edge shows peak splitting across its transition temperature, with two clear peaks emerging below T_{MIT}. This peak splitting has been studied previously and is an indicator of the insulating regime[14]. Additionally, the relative heights of the two L_3 peaks indicate the lack of oxygen vacancies in the system – in contrast to these spectra, a strong A (lower L_3 energy) peak with a diminished B peak (higher L_3 energy) is associated with oxygen non-stoichiometry and a combination of Ni^{3+} and Ni^{2+} valence states[14,15]

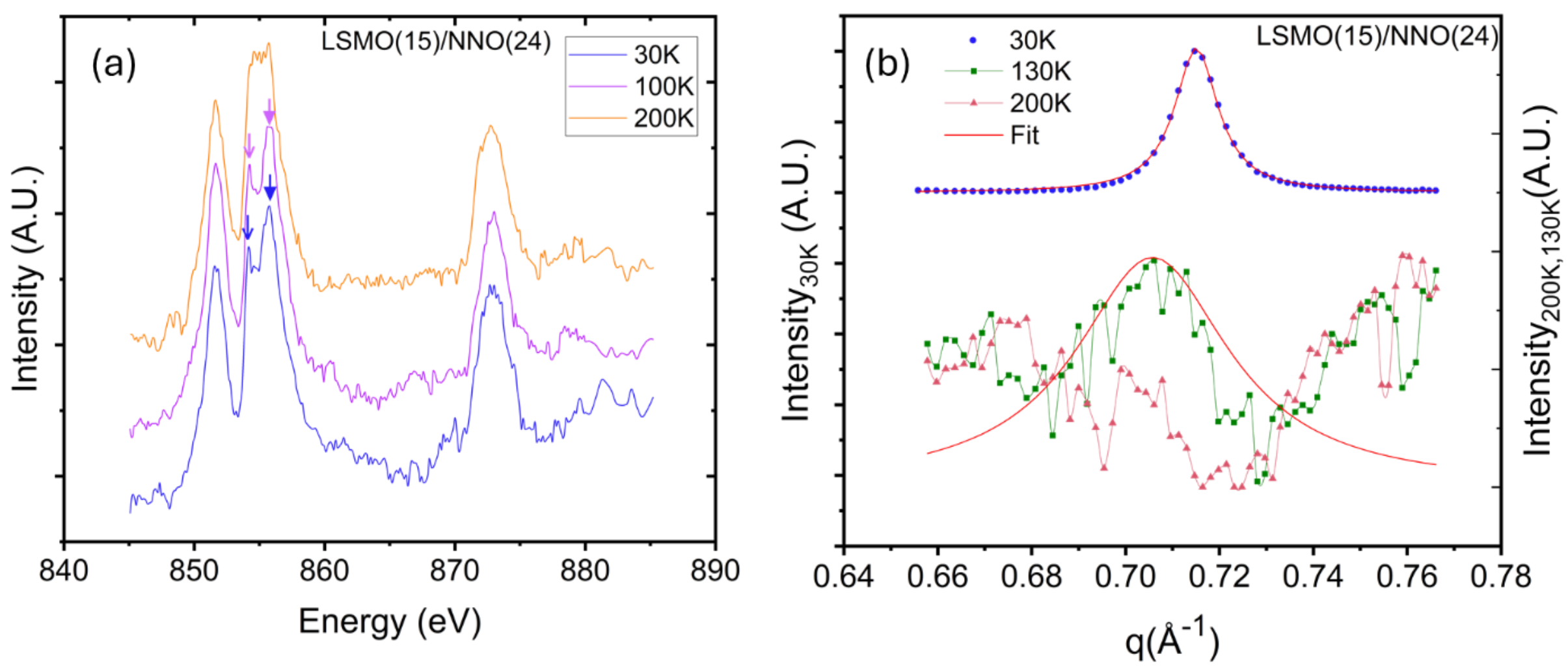

Figure S13 X-ray absorption spectroscopy (a) and resonant X-ray scattering (b) measurements over a range of temperatures for NGO (110)/LSMO (15 nm)/NNO (24 nm).

Reciprocal Space Mapping:

Reciprocal space mapping was performed at room temperature using a Huber four-circle diffractometer in fixed phi (three-circle) mode with a Cu K-alpha source. Specular $(220)_o$ and off-specular $(33\text{-}2)_o$ and $(420)_o$ peaks were measured in LSMO (15 nm)/NNO (24 nm) and LSMO (15 nm) samples on NGO $(110)_o$ substrates to check the epitaxial nature of the films. Reciprocal space maps (RSMs) for these two samples are shown in Fig S14, and the NGO substrate, LSMO, and NNO film peaks are indicated with a ring, a star, and an arrow, respectively. The dashed white lines mark the [110] growth direction in each RSM. The substrate and film peaks are clearly aligned along these lines in each RSM, indicating that these films are epitaxial.

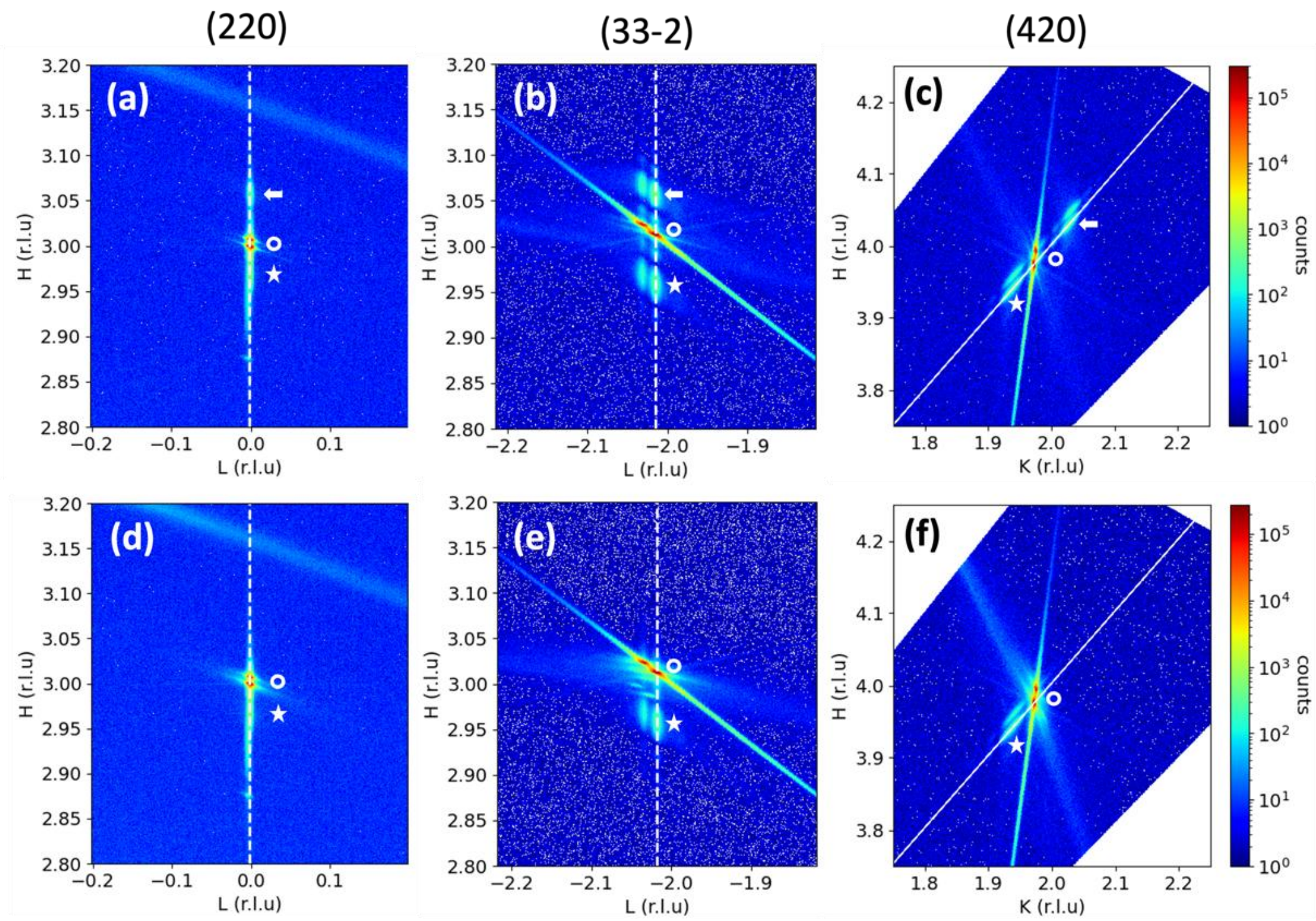

Figure S14 Reciprocal space maps of LSMO (15 nm)/NNO (24 nm) (top row; a-c) and LSMO (15 nm) (bottom row; d-f) samples grown on NGO (110). The specular $(220)_o$ peak is shown in the left column (a, d), and the off-specular $(33\text{-}2)_o$ and $(420)_o$ peaks are shown in the middle (b, e) and right (c, f) columns, respectively. NGO, LSMO, and NNO peaks are marked by a ring, a star, and an arrow, respectively.

References:

1. Demidov, V. V. & Ovsyannikov, G. A. Temperature dependence of interlayer exchange interaction in $La_{0.7}$ $Sr_{0.3}$ $MnO_3/SrRuO_3$ heterostructure. *J. Appl. Phys.* **122**, 013902 (2017).
2. Lee, H. K. *et al.* Magnetic anisotropy, damping, and interfacial spin transport in Pt/LSMO bilayers. *AIP Adv.* **6**, 055212 (2016).
3. Sahoo, B. *et al.* Temperature Dependent Spin Dynamics in $La_{0.67}Sr_{0.33}MnO_3$/Pt Bilayers. *Adv. Mater. Interfaces* **12**, 2401038 (2025).
4. Kittel, C. On the Theory of Ferromagnetic Resonance Absorption. *Phys. Rev.* **73**, 155–161 (1948).
5. Boschker, H. *et al.* Strong uniaxial in-plane magnetic anisotropy of (001)- and (011)-oriented $La_{0.67}Sr_{0.33}MnO_3$ thin films on $NdGaO_3$ substrates. *Phys. Rev. B* **79**, 214425 (2009).

6. Tsui, F., Smoak, M. C., Nath, T. K. & Eom, C. B. Strain-dependent magnetic phase diagram of epitaxial $La_{0.67}Sr_{0.33}MnO_3$ thin films. *Appl. Phys. Lett.* **76**, 2421–2423 (2000).

7. Mattoni, G. *et al.* Striped nanoscale phase separation at the metal–insulator transition of heteroepitaxial nickelates. *Nat. Commun.* **7**, 13141 (2016).

8. Preziosi, D. *et al.* Direct Mapping of Phase Separation across the Metal–Insulator Transition of NdNiO3. *Nano Lett.* **18**, 2226–2232 (2018).

9. Shaw, J. M., Nembach, H. T. & Silva, T. J. Determination of spin pumping as a source of linewidth in sputtered $Co_{90}Fe_{10}$/Pd multilayers by use of broadband ferromagnetic resonance spectroscopy. *Phys. Rev. B* **85**, 054412 (2012).

10. Fache, T., Rojas-Sanchez, J. C., Badie, L., Mangin, S. & Petit-Watelot, S. Determination of spin Hall angle, spin mixing conductance, and spin diffusion length in CoFeB/Ir for spin-orbitronic devices. *Phys. Rev. B* **102**, 064425 (2020).

11. Rossiter, P. L. *The Electrical Resistivity of Metals and Alloys*. (Cambridge University Press, Cambridge, 1987). doi:10.1017/CBO9780511600289.

12. Gupta, A. K. Nonlinear dc response in composites: A percolative study. *Phys. Rev. B* **57**, 3375–3388 (1998).

13. Catalan, G., Bowman, R. M. & Gregg, J. M. Metal-insulator transitions in $NdNiO_3$ thin films. *Phys. Rev. B* **62**, 7892–7900 (2000).

14. Freeland, J. W., van Veenendaal, M. & Chakhalian, J. Evolution of electronic structure across the rare-earth RNiO3 series. *J. Electron Spectrosc. Relat. Phenom.* **208**, 56–62 (2016).

15. Kim, T. H. Strain-driven disproportionation at a correlated oxide metal-insulator transition. *Phys. Rev. B* **101**, (2020).
