# Supplementary material for: Enhanced spin-to-charge conversion in epitaxial La0.67Sr0.33MnO3/NdNiO3 bilayers at the nickelate metal-insulator phase transition
Source: Nat Commun. 2026 Aug 6;17:9461. doi: 10.1038/s41467-026-75812-0 (PMC13542201; doi:10.1038/s41467-026-75812-0)
Supplement: Supplementary file 2 [file 41467_2026_75812_MOESM2_ESM.pdf]

nature portfolio

Peer Review File

Enhanced spin-to-charge conversion in epitaxial $La_{0.67}Sr_{0.33}MnO_3/NdNiO_3$ bilayers at the nickelate metal-insulator phase transition

Corresponding Author: Dr Biswajit Sahoo

Parts of this Peer Review File have been redacted as indicated to remove third-party material.

This file contains all reviewer reports in order by version, followed by all author rebuttals in order by version.

Attachments originally included by the reviewers as part of their assessment can be found at the end of this file.

Version 0:

Reviewer comments:

Reviewer #1

(Remarks to the Author)
The manuscript by Dr. B. Sahoo and co-authors reports about temperature-dependent spin-to-charge conversion (S2CC) phenomena occurring in the rare-earth nickelate NdNiO3. S2CC is probed by spin-pumping ferromagnetic resonance (SP-FMR) by taking the advantage of a pure spin current generated in the neighbouring La0.67Sr0.33MnO3 (LSMO) low-damping ferromagnetic layer. The spin-charge converter element NdNiO3 displays a metal-insulator transition below room temperature, and the main finding of the study is the observation of clear changes in the S2CC performances across the phase transition.

I found this research paper very well written. The applied methodology based on SP-FMR, which is used to track the S2CC across the phase transition of NdNiO3 is rigorously applied. The complementary information provided in the "Supplementary information" file are helpful to fully understand the methodology and the results, and to potentially reproduce them. Overall, the reported results are sound, very clearly presented and discussed. The conclusions are supported by the data presented.

On the other hand, I see two major obstacles for this manuscript to be considered for Nature Communications:

1. The significance of the results in the field of spintronics, and in particular to open "...pathway for developing multifunctional, energy-efficient spintronic devices" as stated by the authors, is limited by the fact that the metal-insulator transition temperature of NdNiO3 is well below room temperature.

2. To monitor spin-charge based phenomena across phase transitions is brilliant, and of high interest for both novel potentially multifunctional applications, and to reveal more fundamental aspects in material science. However, the idea of monitoring S2CC across the phase transition of the "converter" element is not new, with several reports available:

- In [Safi, T.S., Zhang, P., Fan, Y. et al. "Variable spin-charge conversion across metal-insulator transition", Nat Commun 11, 476 (2020). https://doi.org/10.1038/s41467-020-143889], the group presents the successful modulation of charge-spin conversion efficiency, as measured by SP-FMR across the metal-insulator transition occurring in VO2. This paper is cited by the authors (ref. 59), but only to compare with a YIG/Pt system, while there is not any discussion/comparison with the relevant results connected to the S2CC change across the VO2 phase transition.

- In [Xia Yang et al "Spin-to-charge conversion in tantalum with structural phase transition", Phys. Scr. 98, 095115 (2023), DOI: 10.1088/1402-4896/acf0fc], the S2CC efficiency across the alfa-beta phases of Ta (occurring above room T) is reported by SP-FMR.

- In [Morota, M. et al, "Spin-to-charge conversion in tantalum with structural phase transition", Phys. Status Solidi RRL 15, 2100247 (2021), https://doi.org/10.1002/pssr.202100247], the different spin pumping performances occurring in crystalline

and amorphous Sb_2Te_3 has been reported.

While I fully recognize that this is the first report of S2CC in $NdNiO_3$ across its phase transition, due to the above considerations I do not see this manuscript significant enough for Nature Communications.

Other minor comments follow.

A) in this kind of experiments, to estimante the S2CC it is very important to be sure about the same chemical-structural-magnetic properties of the magnetic layer (here LSMO) when "alone" (reference sample) and when coupled with the converter (here $NdNiO_3$). At lines 177-179 of the manuscript the authors define the difference of the obtained damping parameters for the bare LSMO and the bilayered structure. It is not clear to me if the reference LSMO layer is grown simultaneously with the other LSMO that are subsequently capped with the different $NdNiO_3$. In either case (yes or no), the authors should convincingly report about the possible variation of the chemical-structural-magnetic properties for the LSMO in their set of sample, since that could heavily impact the extracted S2CC efficiency.

B) The impact of the study may be enhanced by contextualizing the presented results about S2CC efficiency with those obtained in other systems of recent high interest, such as topological insulators, transition metal dichalcogenides, etc… , and of course all the relevant S2CC studies conducted on systems undergoing phase transitions.

Reviewer #2

(Remarks to the Author)
In this manuscrip, the authors examine the spin-to-charge conversion in $NdNiO_3$ (NNO). In particular, the authors use spin pumping ferromagnetic resonance to observe temperature and thickness evolution in $La_{0.67}Sr_{0.33}MnO_3$ (LSMO)-NNO bilayers.
The authors first demonstrate the role that NNO plays in the emergent properties of the heterostructure by showing a reduction in the Neel transition temperature T_N relative to the metal-to-insulator transition temperature T_{MIT}; this separation scales with NNO layer thickness. The key observation comes from the ferromagnetic resonance measurements; the charge current produced by NNO is roughly flat with temperature for LSMO(12)/NNO(4) but increases well above T_N for LSMO(12)/NNO(8) and LSMO(12)/NNO(16).

These reported results are interesting, systematic and scientifically sound. There are several places, however, where the manuscript could be improved:

1. The behavior between T_{MIT} and T_N is attributed in part to the first-order phase transition in NNO, allowing for enhanced spin-scattering via formation of “AFM insulating domains within a much larger metallic matrix.” Probing the domain formation in these devices would greatly enhance the understanding especially towards the microscopic picture. Comments including previous results on the domain formation and estimates on the critical domain size for spin-charge enhancement would be needed.

2. The overall impact of this work on the field is not highlighted enough. The concluding paragraph mentions applications of these devices, but the discussion should be enhanced by stating how this methodology can be used to develop other devices.

3. Related to the previous point, the discussion (for example, lines 236-244) struggles to pinpoint the ways this system is uniquely suited to enhance spin-to-charge conversion. Many intrinsic properties of transitional metal oxide systems are listed, but the discussion would strongly benefit from more specificity and focus on the types of systems that could host a similar effect, or how the effect could be even further enhanced in other possible manganite/nickelate structures.

4. The authors mention epitaxial strain in the discussion e.g. line 241. The supplementary information includes x-ray diffraction characterization of the strain but given the importance of strain in this central claim of the paper, it would be important to present reciprocal space map measurements which more definitely assess the epitaxial relation of the bilayer.

Overall, this work is repeatable and scientifically interesting, and it can be quite relevant for the larger research community searching for emergent phenomena in transition metal oxide heterostructures. However, as presently written, the manuscript lacks clarity in the microscopic picture of the spin-charge conversion phenomenon, which at the present form hinders it from making a broader impact. If these revisions cannot be made, I would recommend this publication be sent out to a more specialized journal.

Reviewer #3

(Remarks to the Author)
See attached file.

Reviewer #4

(Remarks to the Author)

I co-reviewed this manuscript with one of the reviewers who provided the listed reports. This is part of the Nature Communications initiative to facilitate training in peer review and to provide appropriate recognition for Early Career Researchers who co-review manuscripts.

Version 1:

Reviewer comments:

Reviewer #1

(Remarks to the Author)
This is a resubmitted version of the manuscript by Dr. B. Sahoo and co-authors, where they report about temperature-dependent spin-to-charge conversion (S2CC) phenomena occurring in the rare-earth nickelate NdNiO3. S2CC is probed by spin-pumping ferromagnetic resonance (SP-FMR) by taking the advantage of a pure spin current generated in the neighbouring La0.67Sr0.33MnO3 (LSMO) low-damping ferromagnetic layer. The spin-charge converter element NdNiO3 displays a metal-insulator transition (MIT) below room temperature, and the main finding of the study is the observation of clear changes in the S2CC performances across the phase transition.

I found already the first submitted version (along with the “Supplementary information” file) very well written, with the applied methodology based on SP-FMR rigorously applied and described. Following the first submission I saw 2 major obstacles before considering that paper for Nat. Comm, plus 2 minor comments. At this second submission, the authors reply to all my comments, and my new thoughts follow.

I start with my minor comments:

A) Here I wasn’t clear maybe, and I try to reformulate my concern. What I asked is to clarify potential chemical-structural-magnetic properties differences between LSMO/NNO and the reference “LSMO” alone. This is necessary because the S2CC functionality and magnitude, are connected to the difference between the damping of bilayer samples (LSMO/NNO) and the LSMO alone (reference): $\alpha bilayer - \alpha LSMO$. In their revised files, the authors provide additional, good-quality and helpful, information about chemical composition of LSMO in LSMO/NNO bilayer. Hower, still, i do not see corresponding information for the references LSMO sample alone. I want to be sure that the reference LSMO shows the same chemical-structural-magnetic properties that those reported for it in the LSMN/NNO bilayer. This is necessarry in order to consider meaningful the „$\alpha bilayer - \alpha LSMO$ ” quantity, which enters the estimation of the effective spin mixing conductance. For instance, what worries me are the VSM data in Fig.S5, from where it is clear tha the magnetic properties of the LSMO alone are not overalpping those in the coupled LSMO/NNO. I think that a comment on this matter will be helpful, i.e. by eplaining if and how these differences can influence the observed change in their damping parameters... and therefore the final estimation of S2CC. Maybe the newly added Fig.S14 could be helpful if contextualized properly.
B) The authors properly addressed my comment.

Regarding my mayor concerns:

1. I criticized the argument made by the authors that the presented results could open “...pathway for developing multifunctional, energy-efficient spintronic devices”. I mainly claimed that since the MIT temperature of NdNiO3 is well below room temperature and therefore prevents real applications.
The authors provide now several arguments. I am particularly happy to notice that strategies to improve the working temperature are available, at least in principle. In particular, the authors mention the possible doping Nd with Sm for increasing the MIT temperature of NNO; while its hydrogen doping can open prospective to manipulate reconfigurable resistance states. I think that in the revised manuscript, the authors now better explain how their results can impact spintronics with additional functionalities.

2. I pointed out that to monitor S2CC across the phase transition of the „converter” element was not new, listing some previous reports. In their revised manuscript, the authors made a big improvement by including relevant cases in the discussion, highlighting the novel aspects of their LSMO/NNO system. I am fine with that.

Reviewer #2

(Remarks to the Author)
After reviewing the revised manuscript and the ref. response, I believe my questions have been adequately addressed, and the manuscript is suitable for publication.

Reviewer #3

(Remarks to the Author)
The revised manuscript is well written and has clearly improved in response to the previous review. The authors have adequately addressed the comments, and the work appears technically sound and ready for publication.

Nevertheless, the study remains largely descriptive and does not provide sufficient microscopic insight or conceptual novelty to justify publication in Nature Communications. The results do not constitute a broadly impactful or unexpected advance for

the journal’s wide readership.

In summary, while the manuscript is suitable for publication in its current form, it would be more appropriate for a specialised journal in spintronics or condensed-matter physics rather than Nature Communications.

Reviewer #4

(Remarks to the Author)
I co-reviewed this manuscript with one of the reviewers who provided the listed reports. This is part of the Nature Communications initiative to facilitate training in peer review and to provide appropriate recognition for Early Career Researchers who co-review manuscripts.

Version 2:

Reviewer comments:

Reviewer #1

(Remarks to the Author)
I thank the authors for their additional and very important data comparing the damping of LSMO grown at 950 °C and + annealed at 950 °C with those of the LSMO grown at 950 °C + annealed at 710 °C (“simulating” the condition of the functional bi-layer sample). The newly added Fig. S6(c) in the Supp. Info, fully answers my latest concern, and I am more that happy to suggest the final acceptance of the manuscript at Nature Communications.

Reviewer #5

(Remarks to the Author)
I was invited as a fourth referee to evaluate the second revised version of the manuscript by Dr. B. Sahoo and co-authors, with access to the previous referee reports and the authors’ responses.
The manuscript is clearly written and addresses a relevant topic in the field of correlated oxides. The central observation, the change in spin-to-charge conversion (S2CC) in $NdNiO_3/La_{0.67}Sr_{0.33}MnO_3$ heterostructures across the metal–insulator transition (MIT) in $NdNiO_3$, is interesting and potentially of significance.
However, I share the concern raised by Referee #3 that the study remains largely descriptive. While I do not consider this alone as a reason for rejection, the manuscript currently falls short of the level of explanation, typically supported by some form of calculation, expected for publication at this level.
Moreover, several claims appear overstated. I will focus on one representative example: the statement that the work “provides new insights into the interplay of the NNO phase transition with the LSMO magnetization dynamics” (line 86) is not fully supported by the presented data. Similar statements also appear in the conclusions/summary. Establishing such an interplay would require a more systematic investigation of the magnetic properties of the LSMO layer (e.g., via thickness or doping dependence) to directly probe its magnetic and transport response.
Another aspect that should be more carefully addressed is the quality of the $NdNiO_3$ layer. The R(T) data (Supplementary Fig. S3) show a broad transition with no clear hysteresis, suggesting that the film may not be of "optimal" quality (i.e., lacking a sharp MIT transition and well-defined hysteresis). While this does not invalidate the results, it should be more explicitly discussed, particularly in relation to phase coexistence and disorder effects. The authors partially address this point through resonant soft X-ray scattering and additional transport data indicating a relationship between insulating and metallic domains and their formation; however, the underlying mechanisms and implications are not fully developed.
Overall, the manuscript presents an interesting experimental observation; however, the level of interpretation should be further refined. I therefore recommend publication after revision, provided that the authors moderate their claims and more carefully align their discussion and conclusions with the presented experimental evidence.

Version 3:

Reviewer comments:

Reviewer #5

(Remarks to the Author)
The authors have made substantial efforts to revise and improve the manuscript and have addressed the majority of my concerns in a satisfactory manner. While a few minor questions remain, I believe that the manuscript presents valuable results that will be of interest to the community.
Therefore, I support its publication in the present form.

Open Access This Peer Review File is licensed under a Creative Commons Attribution 4.0 International License, which permits use, sharing, adaptation, distribution and reproduction in any medium or format, as long as you give appropriate credit to the original author(s) and the source, provide a link to the Creative Commons license, and indicate if changes were made.
In cases where reviewers are anonymous, credit should be given to 'Anonymous Referee' and the source.
The images or other third party material in this Peer Review File are included in the article's Creative Commons license, unless indicated otherwise in a credit line to the material. If material is not included in the article's Creative Commons license and your intended use is not permitted by statutory regulation or exceeds the permitted use, you will need to obtain permission directly from the copyright holder.
To view a copy of this license, visit https://creativecommons.org/licenses/by/4.0/

Author comments have been mentioned in blue.

All the changes have been marked in red in the revised manuscript.

Reviewer #1 (Remarks to the Author):

The manuscript by Dr. B. Sahoo and co-authors reports about temperature-dependent spin-to-charge conversion (S2CC) phenomena occurring in the rare-earth nickelate NdNiO3. S2CC is probed by spin-pumping ferromagnetic resonance (SP-FMR) by taking the advantage of a pure spin current generated in the neighbouring La0.67Sr0.33MnO3 (LSMO) low-damping ferromagnetic layer. The spin-charge converter element NdNiO3 displays a metal-insulator transition below room temperature, and the main finding of the study is the observation of clear changes in the S2CC performances across the phase transition.

I found this research paper very well written. The applied methodology based on SP-FMR, which is used to track the S2CC across the phase transition of NdNiO3 is rigorously applied. The complementary information provided in the "Supplementary information" file are helpful to fully understand the methodology and the results, and to potentially reproduce them. Overall, the reported results are sound, very clearly presented and discussed. The conclusions are supported by the data presented.

On the other hand, I see two major obstacles for this manuscript to be considered for Nature Communications:

1. The significance of the results in the field of spintronics, and in particular to open "...pathway for developing multifunctional, energy-efficient spintronic devices" as stated by the authors, is limited by the fact that the metal-insulator transition temperature of NdNiO3 is well below room temperature.

We agree with the reviewer's observation that the low temperature MIT of NNO hinders the direct use of NNO for room temperature applications of spintronic devices. To this end, we would want to point out that the primary focus of the paper is exploring the subtle physics in NNO in relation to the spin dynamics in this material through its metal-insulator phase transition, which is achieved in part, due to the coherent and epitaxial growth of NNO on LSMO.

We emphasize that **Nature Communications publishes mechanism-driven studies** that open new research directions and potential approaches for new applications. Our work highlights two major points:

1.) **The paramagnetic metallic regime has a higher spin-charge conversion than the anti-ferromagnetic insulating regime.**
2.) **The intrinsic domain-based disorder that emerges at the MIT leads to enhancement of the spin-charge conversion.**

We believe these results are enabled by the coherent interfaces as reflected in the continuous octahedral network observed by TEM. This ensures true atomic and electronic coupling between the layers. Such a well-connected interface allows external perturbations to modulate the exchange interactions in a fundamentally different manner compared to metallic heterostructures. When subjected to variations in temperature and magnetic field, the bilayer system thus provides a highly correlated and complex platform for the emergence of self-organized interfacial phenomena: an effect that is unique and represents a new direction in spin–charge interconversion studies. To reflect this important point, we have modified the title: Enhanced spin-to-charge conversion in **epitaxial** La0.67Sr0.33MnO3/NdNiO3 bilayers at the nickelate metal-insulator phase transition.

We have demonstrated that one can grow ultra-thin (<16nm) low damping LSMO/NNO bilayers, with damping values less than 10 times that of conventional ferromagnets, whilst preserving the phase transition property of NNO. For near-room temperature applications, one can dope Nd with Sm to increase the MIT temperature of the non-magnetic layer[1]. NNO by itself also offers a rich playground to explore. Hydrogen doping of NNO has been shown to change the resistance leading to reconfigurable resistance states, which can be used in nickelate based artificial intelligence.[2] Further, application of voltage can further tune the resistances of NNO.[3] In all these studies, only the charge property of NNO has been explored. We are now shedding light on the “spin” property of this highly correlated electronic system, which we show is intricately entangled with the “charge” property of the system (i.e. the MIT). This opens utilizing the spin property of NNO and by extension potentially other nickelates for spintronic devices.

In the revised manuscript, we have further probed into the domain formation in NNO through its phase transition by mapping the magnetic scattering peak of the NNO layer in LSMO/NNO bilayer through resonant soft x ray scattering. We determine the average domain size through correlation length of the magnetic order (~18 nm) and further determine the sparseness of the insulating volume fraction at the onset of the phase transition (~1-4%), providing insight into the role of disorder-based scattering for the enhancement of the spin-charge conversion.

2. To monitor spin-charge based phenomena across phase transitions is brilliant, and of high interest for both novel potentially multifunctional applications, and to reveal more fundamental aspects in material science. However, the idea of monitoring S2CC across the phase transition of the "converter" element is not new, with several reports

available:

- In [Safi, T.S., Zhang, P., Fan, Y. et al. "Variable spin-charge conversion across metal-insulator transition", Nat Commun 11, 476 (2020). https://doi.org/10.1038/s41467-020-143889], the group presents the successful modulation of charge-spin conversion efficiency, as measured by SP-FMR across the metal-insulator transition occurring in VO2. This paper is cited by the authors (ref. 59), but only to compare with a YIG/Pt system, while there is not any discussion/comparison with the relevant results connected to the S2CC change across the VO2 phase transition.

- In [Xia Yang et al "Spin-to-charge conversion in tantalum with structural phase transition", Phys. Scr. 98, 095115 (2023), DOI: 10.1088/1402-4896/acf0fc], the S2CC efficiency across the alfa-beta phases of Ta (occurring above room T) is reported by SP-FMR.

- In [Morota, M. et al, "Spin-to-charge conversion in tantalum with structural phase transition", Phys. Status Solidi RRL 15, 2100247 (2021), https://doi.org/10.1002/pssr.202100247], the different spin pumping performances occurring in crystalline and amorphous Sb2Te3 has been reported.

While I fully recognize that this is the first report of S2CC in NdNiO3 across its phase transition, due to the above considerations I do not see this manuscript significant enough for Nature Communications.

We agree with the referee that our paper does not represent the first report of the MIT on spin-to-charge conversion. However, we believe there are several unique aspects our work that warrants publication in Nature Communications. In our manuscript the LSMO/NNO are **very thin** (t_{LSMO}=12nm, t_{NNO}<=16nm), with **coherent epitaxial growth**, which can allow one to shed light into the complex **interplay of the role of magnetic metal to insulator phase transition on spin to charge conversion**. Even the thinnest NNO case (LSMO(12)/NNO(4)), we see clear indication of MIT, demonstrating the high quality of our films. Secondly, we **demonstrate enhancement of the spin-to charge conversion at T_{MIT}** which is not observed in other systems.

A thorough reading and comparison of our manuscript to the papers cited by the reviewer highlights the following differences:

1. Safi Et al does discuss the role of the phase transition in VO2 on the spin-to-charge conversion. The referee is correct that we should have compared the results of this paper to our result. Similar results have also been reported by Kim, J. *et al*. *Advanced Functional Materials* **32**, 2111555 (2022) for VO2/FeNi. We

have addressed this in the revised manuscript highlighting the unique features of our work. In particular, the nature of the phase transition in VO2 is such that no enhancement of the spin-to-charge conversion is observed at the phase transition.

2. Yang *et al* discusses different samples with varying crystalline phases that were prepared and measured at room temperature. While interesting, this probes the spin-to-charge conversion for different Ta phases and thus it is not possible to probe the evolution through the phase transitions.
3. Morota *et al*: As discussed with Yang et al. about the two phases studied (amorphous and crystalline of Sb_2Te_3) were controlled by tuning the fabrication process to obtain separate samples of each phase. They are not able to track the phase evolution of the response through the phase transition. We also point out that while this paper does mention the term "spin-pumping", the actual measurements reported in this paper evaluates the spin-pumping contribution via FMR only, and NOT by measuring the voltage generated by the corresponding inverse spin Hall effect. An enhancement of the Gilbert damping is taken as an indication of the spin-pumping contribution.

In the revised manuscript, we have included discussion on materials systems from where the phase transition can be monitored (VO_2/Py and FeRh)[4,5] for comparison in our revised manuscript. Additionally, we have now added insights into the domain formation in $NdNiO_3$ by performing Resonant soft X-ray scattering in LSMO/NNO films. We quantitatively present the insulating AFM correlation length (and thus the domain size) to be ~18 nm in the insulating phase. We verify that the volume fraction of the insulating domains at the T_N is around 53-55% similar to other reports[6,7], which is the critical temperature for loss of connectivity of the metallic percolation network, and further show that at the onset of transition (T_{MIT}), the insulating domains are sparse in nature with volume fraction of ~1-4%, which agrees to our hypothesis of enhancement of spin-charge conversion due to enhanced spin scattering by sparse extrinsic scatters[8–10].

We thus strongly believe that the rigor and comprehensiveness of the material system presented and the unique physics demonstrated warrants publication in Nature Communication and we hope that our revised manuscript will convince the reviewer to agree to our assertion.

Other minor comments follow.

A) in this kind of experiments, to estimante the S2CC it is very important to be sure about the same chemical-structural-magnetic properties of the magnetic layer (here

LSMO) when "alone" (reference sample) and when coupled with the converter (here NdNiO3). At lines 177-179 of the manuscript the authors define the difference of the obtained damping parameters for the bare LSMO and the bilayered structure. It is not clear to me if the reference LSMO layer is grown simultaneously with the other LSMO that are subsequently capped with the different NdNiO3. In either case (yes or no), the authors should convincingly report about the possible variation of the chemical-structural-magnetic properties for the LSMO in their set of sample, since that could heavily impact the extracted S2CC efficiency.

The LSMO/NNO bilayers have been fabricated in-situ with the fabrication details mentioned in the Methods section. It is evidenced from EDX plots (Fig. S2 (a)-(f)) that the chemical distribution of elements in the bilayers is sharp and well defined

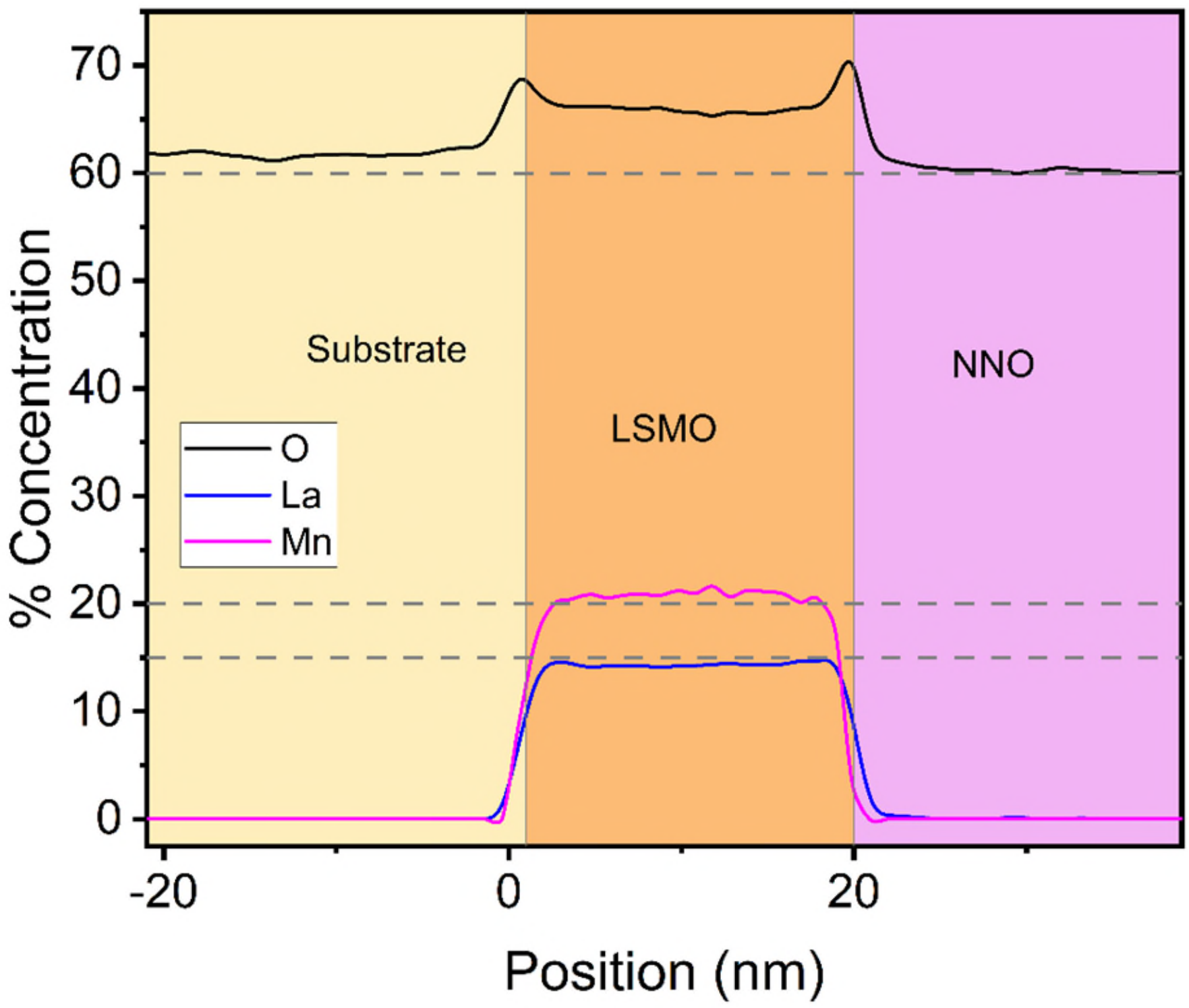

Figure R1 EELS concentration map of LSMO (17)/NNO (24) showing La, O and Mn concentration maps over the bilayer.

Elemental concentration plot obtained from EELS measurements (Fig. R1) shows approx. 14% La, 20% Mn, and 65% O in LSMO. (We could not obtain Sr signal as it is at higher energies) which is close to the predicted La (Mn) percentage: 15% (20%). The higher O_2 concentration is responsible for the sufficient magnetization of LSMO at these growth conditions which allows (and is necessary for) robust detection of the FMR/SP-FMR signal.

We have addressed this in the supplementary section Figure S2(h).

B) The impact of the study may be enhanced by contextualizing the presented results about S2CC efficiency with those obtained in other systems of recent high interest, such as topological insulators, transition metal dichalcogenides, etc... , and of course all the relevant S2CC studies conducted on systems undergoing phase transitions.

In the revised manuscript we have now included systems that undergo metal insulator phase transition (VO_2)[5,11], and magnetic phase transition (FeRh[4]) and compared the differences of the spin dynamics in these systems as opposed to that in $NdNiO_3$. Additionally, we have compared our results with that of other FM or NM oxide materials[12–19], highlighting the uniqueness of NNO. It is worth noting that investigations of similar phenomena in perovskite transition metal oxides are relatively scarce. Nevertheless, recent developments in this material family, especially the discovery of superconducting phases in $NdNiO_{3-\delta}$[20], highlight the growing importance of such studies. This will open another aspect of research which are fundamentally different than the conventional approaches to study S2CC in as topological insulators, transition metal dichalcogenides, etc, as it provides a unique structural control over complex spin, charge, lattice and orbital degrees of freedom all together.

Reviewer #2 (Remarks to the Author):

In this manuscrip, the authors examine the spin-to-charge conversion in NdNiO3 (NNO). In particular, the authors use spin pumping ferromagnetic resonance to observe temperature and thickness evolution in La0.67Sr0.33MnO3 (LSMO)-NNO bilayers. The authors first demonstrate the role that NNO plays in the emergent properties of the heterostructure by showing a reduction in the Neel transition temperature T_N relative to the metal-to-insulator transition temperature T_MIT; this separation scales with NNO layer thickness. The key observation comes from the ferromagnetic resonance measurements; the charge current produced by NNO is roughly flat with temperature for LSMO(12)/NNO(4) but increases well above T_N for LSMO(12)/NNO(8) and LSMO(12)/NNO(16).

These reported results are interesting, systematic and scientifically sound. There are several places, however, where the manuscript could be improved:

We thank the reviewer for their thorough reading of the work and pointing out important caveats and suggesting improvements to the manuscript. We address them point by point as follows:

1. The behavior between T_MIT and T_N is attributed in part to the first-order phase

transition in NNO, allowing for enhanced spin-scattering via formation of "AFM insulating domains within a much larger metallic matrix." Probing the domain formation in these devices would greatly enhance the understanding especially towards the microscopic picture. Comments including previous results on the domain formation and estimates on the critical domain size for spin-charge enhancement would be needed.

From Resonant soft x-ray measurements, we have quantified the correlation length to be around ~18 nm in the insulating phase and used it to obtain the relative volume fraction of the insulating phase at T_N to be 53%. Further, by juxtaposing the x-ray measurements with electrical data from single layer $NdNiO_3$ film of the same thickness, we have now evaluated the volume fraction to be 1-4% around T_{MIT}. These changes have been reflected in the revised manuscript and supplementary.

2. The overall impact of this work on the field is not highlighted enough. The concluding paragraph mentions applications of these devices, but the discussion should be enhanced by stating how this methodology can be used to develop other devices.

We answer the question together in point 3

3. Related to the previous point, the discussion (for example, lines 236-244) struggles to pinpoint the ways this system is uniquely suited to enhance spin-to-charge conversion. Many intrinsic properties of transitional metal oxide systems are listed, but the discussion would strongly benefit from more specificity and focus on the types of systems that could host a similar effect, or how the effect could be even further enhanced in other possible manganite/nickelate structures.

Nickelates offer multiple tuning knobs which make them attractive for use in multifunctional spintronics. The T_{MIT} can be elevated by doping $NdNiO_3$ with Sm[21] enabling the realization of room-temperature nickelate based spintronic devices. By engineering T_{MIT} at the desired temperature, one can engineer the phases in such nickelates that exhibit maximum θ_{SHA} and large tunability of spin-charge conversion efficiency. To date, majority of the studies have been focused on manipulating the charge degree of freedom either by hydrogen doping[22], optical excitation [23], voltage stimuli[3,24,25] etc. Our findings on the controllable enhancement of spin-charge conversion open new pathways to exploit the spin degree of freedom in NNO and, by extension, other nickelates. Additionally, the nickelate phases can be tuned locally via optical methods, laser induced thermal effects, or voltage induced strain. Such control mechanisms offer a versatile platform for creating reconfigurable arrays of nickelate-based spintronic devices. On a tangential note, the spin-sinking effect in the AFM NNO with a coherence length of 18 nm, along with no dissipation of spin-charge current in this phase may also allow realization of magnon based spin-transport in tri-layer

systems[26,27] of high-SOC-NM /NNO/LSMO owing to the low damping and robust AFM coupling.

We have now revised the discussion section in the new manuscript to reflect this.

4. The authors mention epitaxial strain in the discussion e.g. line 241. The supplementary information includes x-ray diffraction characterization of the strain but given the importance of strain in this central claim of the paper, it would be important to present reciprocal space map measurements which more definitely assess the epitaxial relation of the bilayer.

We have performed RSM scans on separately grown samples LSMO (15) and LSMO (15)/NNO (24) on 5 x 5 mm NGO (*110*) substrate. The substrate and the films peaks are clearly aligned in the different measured orientations: (*220*), (*33-2*), and (*420*) indicating preservation of the epitaxial nature in LSMO/NNO bilayer. We have included this in the supplementary section figure S14 of the manuscript.

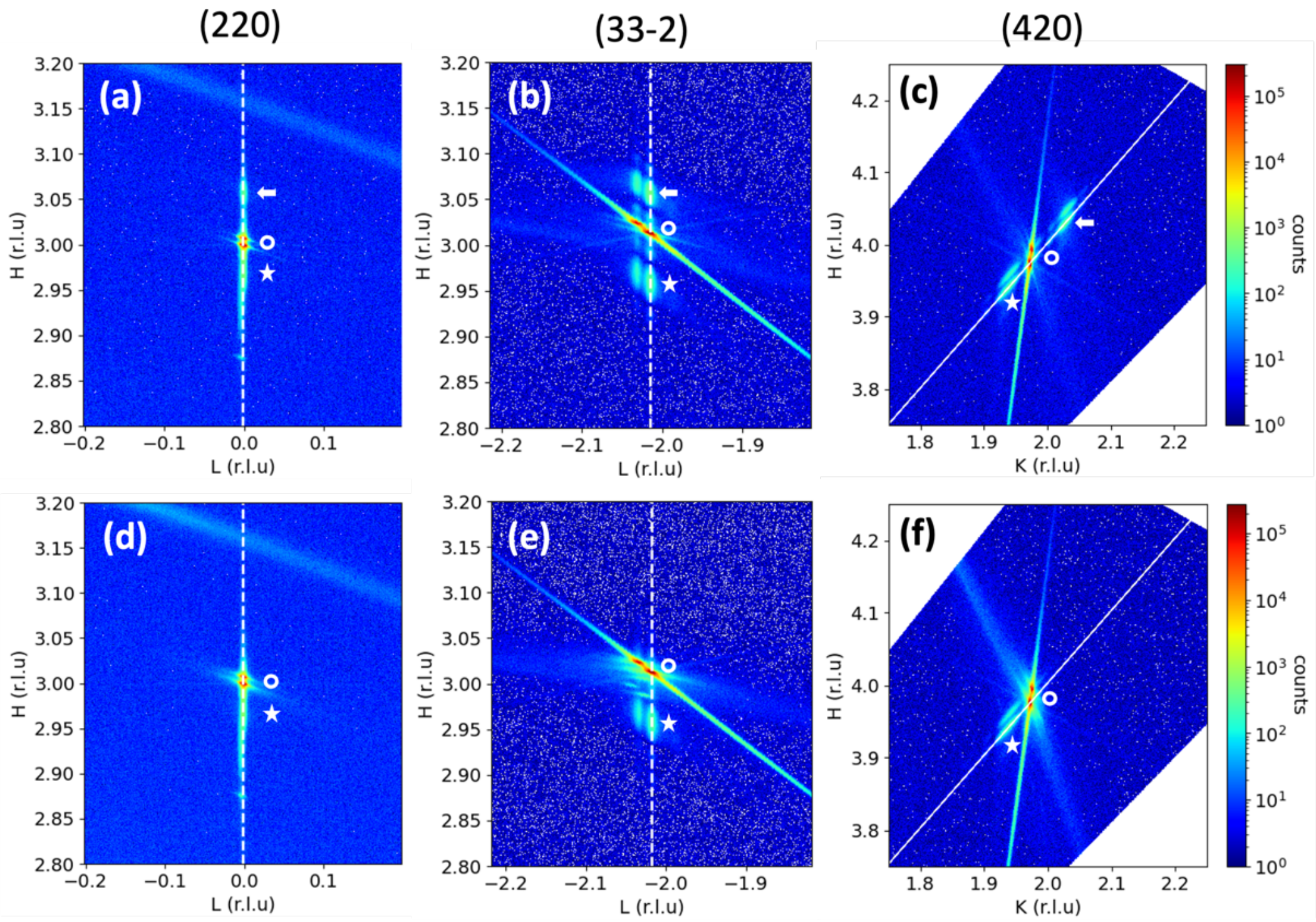

Figure R2 Reciprocal space maps of LSMO (15 nm)/NNO (24 nm) (top row; a-c) and LSMO (15 nm) (bottom row; d-f) samples grown on NGO (110). The specular (220) peak is shown in the left column (a, d), and the off-specular (33-2) and (420) peaks are shown in the middle (b, e) and right (c, f) columns, respectively. NGO, LSMO, and NNO peaks are marked by a ring, a star, and an arrow, respectively.

Overall, this work is reproducible and scientifically interesting, and it can be quite relevant for the larger research community searching for emergent phenomena in transition metal oxide heterostructures. However, as presently written, the manuscript

lacks clarity in the microscopic picture of the spin-charge conversion phenomenon, which at the present form hinders it from making a broader impact. If these revisions cannot be made, I would recommend this publication be sent out to a more specialized journal.

Reviewer #3 (Remarks to the Author):

See attached file.

Reviewer #4 (Remarks to the Author):

I co-reviewed this manuscript with one of the reviewers who provided the listed reports. This is part of the Nature Communications initiative to facilitate training in peer review and to provide appropriate recognition for Early Career Researchers who co-review manuscripts.

NNO is of interest due to a phase transition, from paramagnetic metal to antiferromagnetic insulator at a given transition temperature. This can be controlled in a number of ways, including electron doping, strain engineering or thickness variation. In this work, the authors investigate the effect of such phase transition into the spin dynamics of the bilayer LSMO/NNO.To do so, the authors investigate the spin-to-charge conversion in a range of LSMO/NdNiO3bilayers with three controlled NdNiO (NNO) different thicknesses. The quality of the samples is proved via HTEM imaging and the metal-insulator properties are checked with resistivity curves. Spin-to-charge conversion is measured for all bilayers at different temperatures using ferromagnetic resonance.

The main experimental result is a signature of an enhanced spin pumping as the temperature approaches the transition temperature. At this transition, the authors argue that insulating antiferromagnetic domains or clusters embedded into the still metallic bulk (as it is below the the transition temperature) act as extrinsic scatterers. As the temperature further increases, the spin pumping goes back to its original value. This point, which is the main result of this work, it is unclear to us. As the NNO transits from metallic to insulating phases, the spin to charge conversion should also change, from one side, the metallic phase can hosts ’spins’ whereas the insulating phases, one should have ’magnons’. In view of figure 3 (a), it seems that in both phases have the same spin-to-charge or magnon-to-charge efficiency. Thus, it is not evident to us that at

the transition, the presence of insulating phase domains would enhance this efficiency. The introduction of nickelates as a way of modulating the properties of a spin-charge conversion device via temperature changes is novel but the article lacks a neat result and is rather descriptive in nature. We believe that a deeper analysis of the experimental results is necessary in order to claim an important, novel result which is among the criteria for publication in Nature Communications. The manuscript is a bit confusing and needs of an improvement of the presentation.

The reviewers have made an insightful observation regarding the spin-charge conversion through the phase transition. Unlike systems such as VO_2 which does not have a magnetic order, or FeRh which has AFM or FM order, NNO is a paramagnetic metal above T_{MIT} and an anti-ferromagnetic insulator below this temperature. This transition takes place via formation of domains which has been reported in literature[6,28] and also demonstrated in the revised manuscript.

We have performed resonant soft x-ray scattering measurements by tuning to the Ni L_3 edge and have provided direct evidence of MIT through domain formation by tracking the $(¼\ ¼\ ¼)_{pc}$ (pc=pseudocubic) magnetic peak of NNO in LSMO (15 nm)/NNO (24 nm) bilayer through the phase transition, demonstrating the AFM nature and domain formation process of these insulating domains. Further, we observe that while the damping for LSMO (12)/NNO (16) increases in the AFM phase of NNO, no hysteretic template of damping variation is observed in the warming and cooling cycles for this sample. This means that the damping has a dominant contribution from AFM/FM interaction between LSMO and NNO (owing to the coherent epitaxial growth with defect free octahedra bonding (Fig 1(b)), rather than the interband scattering (or resistivity contribution) as in the case of FeRh[4]. This indicates that the spin current may be propagating into NNO via magnons in the AFM NNO phase resulting in enhanced spin sink behaviour.

In Figure 3(a) let us now consider LSMO (12)/NNO (16). The spin-pumped charge current (I_{C-SP}) has similar value both below (T=150K) and above (T=200K) the T_{MIT}. However, it is to be noted that the magnetization of LSMO increases with reducing temperature and thus **the spin current density (J_s) pumped into NNO is higher at lower temperatures. Thus, upon taking the increased value of J_s into consideration, the θ_{SHA} is less for $T<T_{MIT}$ (Fig 3(b))**. By comparing the I_{C-SP} at T=100K (where NNO is fully insulating) with that at T= 200K, we can clearly see the reduced value of I_{C-SP}, despite the increase in damping at 100K. This indicates that the spin-charge conversion is governed by the conduction electrons in the metallic phase. On a tangential note, the increased damping in the insulating phase indicates we have absorption of spins into AFM-NNO, which are not converted into charge current. Combine this with the evaluated coherence length of 18nm, one can argue that NNO can allow efficient propagation of spin current through it in the insulating phase.

In the revised manuscript, we have further estimated the relative volume fraction (both by electrical measurements and resonant soft x-ray measurements) of the insulating domains in the metallic matrix and have determined the fraction to be in the sparse limit of ~1-4% around the T_{MIT}. We attribute the enhancement of θ_{SHA} at this phase to be due to similar phenomenon as in the case of sparsely doped Pt with insulating MgO or TiO_2 (dopant conc. <3%).[8–10], The enhancement in θ_{SHA} was observed due to enhancement in the extrinsic spin scattering by the sparse insulating dopants.

Some minor comments are shown below:

Main text

• Figures 1a and 1b are not referenced in the main text.

We have made the necessary changes in the revised manuscript.

• Line 55 and Figure 1(e). "LSMO(12 nm)/NNO(0, 4, 8 and 16 nm)" The nomenclature for the LSMO without NNO on top is rather confusing. The readability would improve separating the single LSMO layer and the LSMO/NNO (4, 8 and 16 nm).

We have now made the appropriate changes in the revised manuscript.

• Figure 1. The existence of the hysteresis loop in the x=4 nm bilayer is not clear as it gets easily confused with the x=16 nm curve. The colors of this figure should be improved.

We have now included an inset figure showing the hysteretic RT nature of the LSMO (12)/NNO (4) sample

• Figure S3(c). The crossing point of the derivative with the y=0 axis is not clear at all, especially seeing the noise that is present around 150 K.

This is because the hysteresis loops in NNO 4 nm is not as pronounced as other NNO thickness. The fact that it shows a tiny hysteresis exemplifies the good quality of our samples. The crossing point has been zoomed in and highlighted inset.

• Line 135. "(full scans for all temperatures are plotted in the supplementary section) "This should point to the specific supplementary figure it is referring to.

We have made the appropriate corrections.

• Line 143 and line 250. In the manuscript the spin to charge conversion is measured using the cooling cycle but as the conclusions state, these devices would be useful for a spin valve device. Some measurements or discussion about the heating cycle would add value to the manuscript.

We have performed the FMR experiments on the samples in the heating and cooling regime as the setup allowed for very accurate <1K control on the overshoot of the temperature. However, unlike in the case of FeRh/Py[4] or YIG/VO_2 [11] we could not find a strong resistance dependence of the damping variation on the heating/cooling cycles which could be directly attributed to the insulting nature of NNO. However, we observe an enhancement in the damping in the AFM phase of NNO which suggests that the AFM-FM exchange interaction dominates the inter-band scattering arising from the resistivity contribution[4] which may be attributed to the robust octahedral coupling resulting from coherent growth of the bilayer. We do see hysteresis in the coercivity and resonant x ray measurements suggesting more AFM nature in the warming cycle, however, this variation may have been too minimal to affect the corresponding damping. We have included the variation of damping in the warming and cooling cycle for LSMO (12)/NNO (16) as an example in the supplementary section (Fig. S6(e)) and have explained its implication in our arguments and reflected the corresponding changes in the revised manuscript.

Additionally, the original experimental setup used to perform SP-FMR experiments had an open-cycle liquid nitrogen-based cooling, which allowed good control in the cooling cycle (<5K overshoot), but almost 20-25K overshoot in the warming cycle, preventing us from performing the warming cycle measurements reliably on this system.

• Line 242-244. “These emergent behaviours, driven by structural and magnetic reconstructions at the interface,...” This paragraph lacks explanation or citations.

In response to the referee’s comment, we have revised the Introduction, Motivation, and Discussion sections throughout the manuscript to clarify the role of complex interfaces in perovskite transition-metal oxides, including appropriate citations to support the discussion of the emergent interfacial mechanisms.

We would like to mention that extensive research over the past decades has shown that interfaces in complex oxide heterostructures host unique structural, electronic, and magnetic behaviors that are fundamentally different from the constituent parent layers, highlighting the central role of interfacial physics in these systems. These works established the framework for understanding interfacial magnetism, charge redistribution, and orbital coupling, highlighting phenomena that go beyond conventional effects observed in metallic heterostructures. In our LSMO/NNO bilayers, we observe analogous emergent interfacial behaviors, where atomic-scale coupling, octahedral connectivity, and exchange interactions give rise to spin-to-charge conversion phenomena that are unique to these oxide interfaces. Our observed interfacial spin-conversion effects in NNO/LSMO fall squarely within this framework.

Methods

• Sample growth. Samples are annealed at 710oC in O. In literature it is shown that even at 100oC nickelates develop oxygen vacancies [1]. While there is an oxygen atmosphere during annealing, XPS stochiometry measurements would be useful to check if the O pressure is sufficient.

[1] Wang, Le ; Dash, Sibashisa ; Chang, Lei et al. / Oxygen Vacancy Induced Room-Temperature Metal-Insulator Transition in Nickelate Films and Its Potential Applicationin Photovoltaics. In: ACS Applied Materials and Interfaces. 2016 ; Vol. 8, No. 15. pp.9769-9776.

From elemental concentration map in Fig R1, we see that the O_2 concentration is ~60% in the NNO layer which is stoichiometrically consistent with the ABO_3 structure of this compound. Via X-ray Absorption spectra (XAS) tuned to Ni L_3 edge, we observe peak splitting of the Ni L_3 edge which is an indicator of the insulating regime[28]. The relative heights of the two L_3 peaks indicate **the lack of oxygen vacancies** in the system. In contrast to the presented spectra, a strong lower L_3 energy (thin arrowhead) peak with a diminished higher L_3 energy-(thick arrow head) peak is associated with oxygen non-stoichiometry and a combination of Ni^{3+} and Ni^{2+} valence states[29,30]. We have reflected these changes in revised supplementary section Fig S13(a).

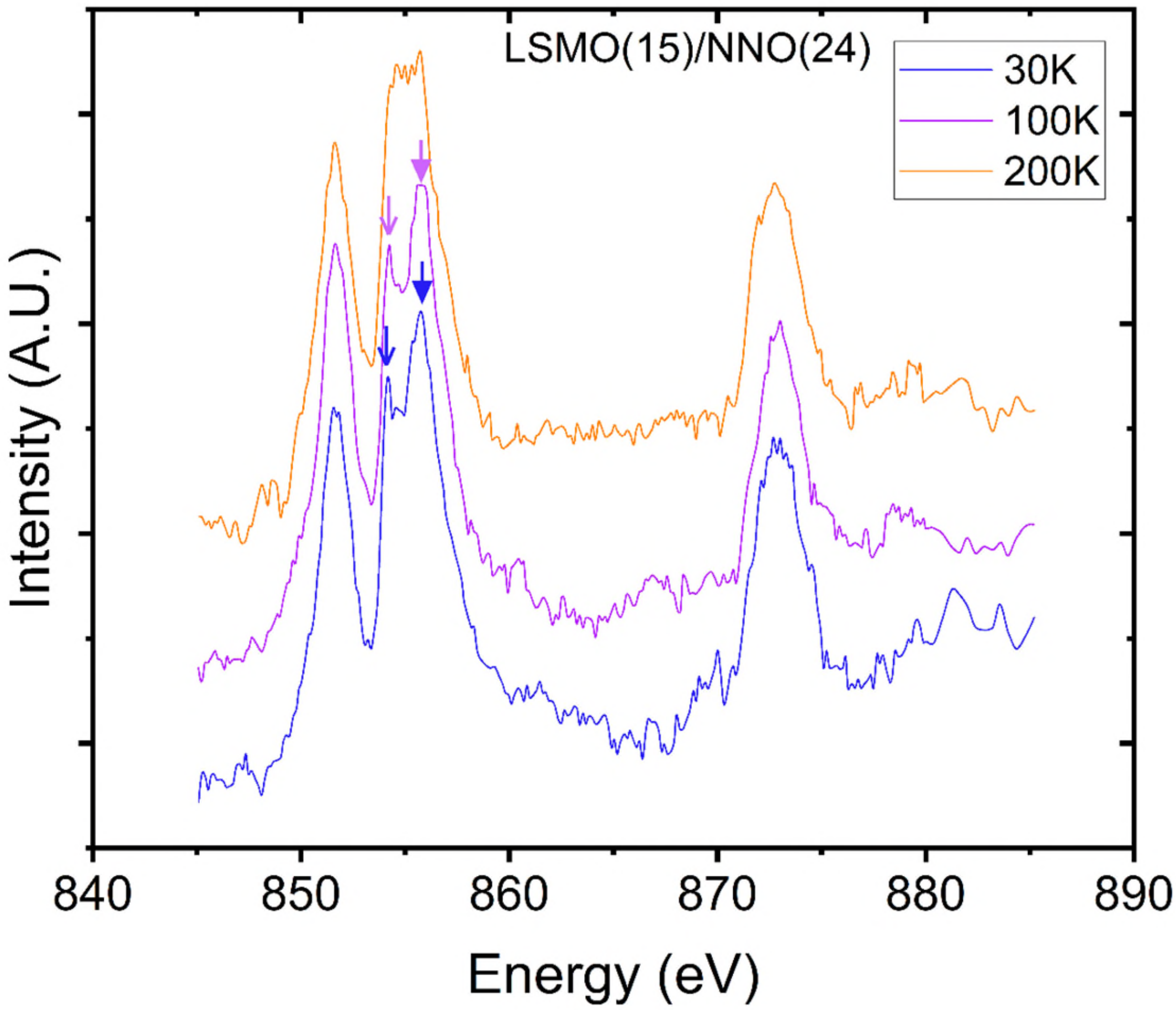

Figure R3 X-ray Absorption spectra (XAS) of Ni L3 edge showing the more intense high energy peak(thick arrowhead) with respect to the low energy peak (thin arrow head), demonstrating no/insignificant oxygen vacancy in NNO.

• Sample growth. A brief description of how the samples were transferred to the different fabrication and measurement chambers would be adequate, as long exposures to air can lead to carbon accumulation that add to the extrinsic spin scattering.

We have now included this in the methods section.

References

1. Catalano, S. *et al.* Rare-earth nickelates $RNiO_3$: thin films and heterostructures. *Rep. Prog. Phys.* **81**, 046501 (2018).
2. Zhang, H.-T. *et al.* Reconfigurable perovskite nickelate electronics for artificial intelligence. *Science* **375**, 533–539 (2022).

3. Scherwitzl, R. *et al.* Electric-Field Control of the Metal-Insulator Transition in Ultrathin $NdNiO_3$ Films. *Advanced Materials* **22**, 5517–5520 (2010).
4. Wang, Y. *et al.* Spin pumping during the antiferromagnetic–ferromagnetic phase transition of iron–rhodium. *Nat Commun* **11**, 275 (2020).
5. Kim, J. *et al.* Tuning Spin-Orbit Torques Across the Phase Transition in VO2/NiFe Heterostructure. *Advanced Functional Materials* **32**, 2111555 (2022).
6. Mattoni, G. *et al.* Striped nanoscale phase separation at the metal–insulator transition of heteroepitaxial nickelates. *Nat Commun* **7**, 13141 (2016).
7. Catalan, G., Bowman, R. M. & Gregg, J. M. Metal-insulator transitions in $NdNiO_3$ thin films. *Phys. Rev. B* **62**, 7892–7900 (2000).
8. Wang, Y. *et al.* Enhancement of spintronic terahertz emission enabled by increasing Hall angle and interfacial skew scattering. *Commun Phys* **6**, 1–10 (2023).
9. Zhu, L., Zhu, L., Sui, M., Ralph, D. C. & Buhrman, R. A. Variation of the giant intrinsic spin Hall conductivity of Pt with carrier lifetime. *Science Advances* **5**, eaav8025 (2019).
10. Xu, X. *et al.* Giant Extrinsic Spin Hall Effect in Platinum-Titanium Oxide Nanocomposite Films. *Advanced Science* **9**, 2105726 (2022).
11. Safi, T. S. *et al.* Variable spin-charge conversion across metal-insulator transition. *Nat Commun* **11**, 476 (2020).
12. Sahoo, B. *et al.* Temperature Dependent Spin Dynamics in $La_{0.67}Sr_{0.33}MnO_3$/Pt Bilayers. *Advanced Materials Interfaces* **12**, 2401038 (2025).
13. Huang, X. *et al.* Novel Spin–Orbit Torque Generation at Room Temperature in an All-Oxide Epitaxial La0.7Sr0.3MnO3/SrIrO3 System. *Advanced Materials* **33**, 2008269 (2021).

14. Benguettat-EL Mokhtari, I. *et al.* Spin Pumping and Magnetic Anisotropy in $La_{2/3}Sr_{1/3}MnO_3$/Pt Systems. *physica status solidi (b)* **257**, 2000265 (2020).

15. Luo, G. Y., Lin, J. G., Chiang, W.-C. & Chang, C.-R. Spin pump and probe in lanthanum strontium manganite/platinum bilayers. *Sci Rep* **7**, 6612 (2017).

16. Sahoo, B., Frano, A. & Fullerton, Eric. E. Efficient charge to spin conversion in iridium oxide thin films. *Applied Physics Letters* **123**, 032404 (2023).

17. Martin-Rio, S. *et al.* Spin-to-Charge Conversion in All-Oxide La2/3Sr1/3MnO3/SrIrO3 Heterostructures. *ACS Appl. Mater. Interfaces* **15**, 37038–37046 (2023).

18. Nan, T. *et al.* Anisotropic spin-orbit torque generation in epitaxial SrIrO3 by symmetry design. *Proceedings of the National Academy of Sciences* **116**, 16186–16191 (2019).

19. Li, P. Large Spin-Orbit-Torque Efficiency and Room-Temperature Magnetization Switching in $SrIrO_3$/CoFeB Heterostructures. *Phys. Rev. Appl.* **19**, (2023).

20. Li, D. *et al.* Superconductivity in an infinite-layer nickelate. *Nature* **572**, 624–627 (2019).

21. Catalano, S. *et al.* Rare-earth nickelates RNiO3: thin films and heterostructures. *Rep. Prog. Phys.* **81**, 046501 (2018).

22. Zhang, H.-T. *et al.* Reconfigurable perovskite nickelate electronics for artificial intelligence. *Science* **375**, 533–539 (2022).

23. Navarro, H. *et al.* Disentangling transport mechanisms in a correlated oxide by photoinduced charge injection. *Phys. Rev. Materials* **7**, L123201 (2023).

24. Dong, Y. *et al.* Effect of gate voltage polarity on the ionic liquid gating behavior of $NdNiO_3/NdGaO_3$ heterostructures. *APL Materials* **5**, 051101 (2017).

25. Asanuma, S. *et al.* Tuning of the metal-insulator transition in electrolyte-gated NdNiO3 thin films. *Applied Physics Letters* **97**, 142110 (2010).

26. Sahoo, B. *et al.* High-Efficiency Continuous Spin-Conduction through NiO/Cu Bilayer Structure. *Nano Lett.* **25**, 3851–3857 (2025).

27. Lee, K. *et al.* Superluminal-like magnon propagation in antiferromagnetic NiO at nanoscale distances. *Nat. Nanotechnol.* **16**, 1337–1341 (2021).

28. Preziosi, D. *et al.* Direct Mapping of Phase Separation across the Metal–Insulator Transition of NdNiO3. *Nano Lett.* **18**, 2226–2232 (2018).

29. Freeland, J. W., van Veenendaal, M. & Chakhalian, J. Evolution of electronic structure across the rare-earth RNiO3 series. *Journal of Electron Spectroscopy and Related Phenomena* **208**, 56–62 (2016).

30. Kim, T. H. Strain-driven disproportionation at a correlated oxide metal-insulator transition. *Phys. Rev. B* **101**, (2020).

Author comments have been mentioned in green.

All the changes have been marked in blue in the revised manuscript.

Reviewer #1 (Remarks to the Author):

This is a resubmitted version of the manuscript by Dr. B. Sahoo and co-authors, where they report about temperature-dependent spin-to-charge conversion (S2CC) phenomena occurring in the rare-earth nickelate NdNiO3. S2CC is probed by spin-pumping ferromagnetic resonance (SP-FMR) by taking the advantage of a pure spin current generated in the neighbouring La0.67Sr0.33MnO3 (LSMO) low-damping ferromagnetic layer. The spin-charge converter element NdNiO3 displays a metal-insulator transition (MIT) below room temperature, and the main finding of the study is the observation of clear changes in the S2CC performances across the phase transition.

I found already the first submitted version (along with the "Supplementary information" file) very well written, with the applied methodology based on SP-FMR rigorously applied and described. Following the first submission I saw 2 major obstacles before considering that paper for Nat. Comm, plus 2 minor comments. At this second submission, the authors reply to all my comments, and my new thoughts follow.

I start with my minor comments:

A) Here I wasn't clear maybe, and I try to reformulate my concern. What I asked is to clarify potential chemical-structural-magnetic properties differences between LSMO/NNO and the reference "LSMO" alone. This is necessary because the S2CC functionality and magnitude, are connected to the difference between the damping of bilayer samples (LSMO/NNO) and the LSMO alone (reference): $\alpha bilayer - \alpha LSMO$. In their revised files, the authors provide additional, good-quality and helpful, information about chemical composition of LSMO in LSMO/NNO bilayer. Hower, still, i do not see corresponding information for the references LSMO sample alone. I want to be sure that the reference LSMO shows the same chemical-structural-magnetic properties that those reported for it in the LSMN/NNO bilayer. This is necessary in order to consider meaningful the „$\alpha bilayer - \alpha LSMO$ " quantity, which enters the estimation of the effective spin mixing conductance. For instance, what worries me are the VSM data in Fig.S5, from where it is clear tha the magnetic properties of the LSMO alone are not overalpping those in the coupled LSMO/NNO. I think that a comment on this matter will

be helpful, i.e. by eplaining if and how these differences can influence the observed change in their damping parameters… and therefore the final estimation of S2CC. Maybe the newly added Fig.S14 could be helpful if contextualized properly.

B) The authors properly addressed my comment.

Regarding my mayor concerns:

1. I criticized the argument made by the authors that the presented results could open "…pathway for developing multifunctional, energy-efficient spintronic devices". I mainly claimed that since the MIT temperature of NdNiO3 is well below room temperature and therefore prevents real applications.

The authors provide now several arguments. I am particularly happy to notice that strategies to improve the working temperature are available, at least in principle. In particular, the authors mention the possible doping Nd with Sm for increasing the MIT temperature of NNO; while its hydrogen doping can open prospective to manipulate reconfigurable resistance states. I think that in the revised manuscript, the authors now better explain how their results can impact spintronics with additional functionalities.

2. I pointed out that to monitor S2CC across the phase transition of the „converter" element was not new, listing some previous reports. In their revised manuscript, the authors made a big improvement by including relevant cases in the discussion, highlighting the novel aspects of their LSMO/NNO system. I am fine with that.

We thank the reviewer for their favourable opinion of our revised manuscript to be published in Nature Communications and for providing us the opportunity to clarify the differences between LSMO in single layer and bilayer heterostructures.

To put the question in context, the LSMO single layer is grown and annealed at 950C to achieve maximum magnetization, whereas for the bilayer sample, the LSMO is grown at 950C, then cooled to 710C in oxygen, whereupon NNO is deposited at the same temperature and then the bilayer is annealed at 710C. This reduced deposition/anneal temperature is crucial for NNO to preserve its MIT, while maintaining sufficient magnetization of LSMO. We will break down the effect of this growth difference of LSMO into two parts: Structural-Chemical and Magnetic.

Structural-Chemical: Both the single layer and the bilayers are **structurally coherent and epitaxial in nature** which is shown by a comparison of the RSM of LSMO and

LSMO/NNO sample (Fig S14). We have previously verified the proper chemical composition of single layer NGO(110)/LSMO films in one of our previous papers[1] via EELS spectroscopy, the composition being similar to LSMO in bilayer films.

Magnetic: This is where a difference is observed in the single layer LSMO vs. LSMO/NNO. The LSMO single layer is grown and annealed at 950C to achieve maximum magnetization, whereas for the bilayer sample, the LSMO is grown at 950C, then cooled to 710C in oxygen, whereupon NNO is deposited at the same temperature and then the bilayer is annealed at 710C.

Due to this lowering of annealing temperature, the magnetization of LSMO reduces in the bilayer samples (and remains the same across different bilayer samples), which is reflected in the M_S vs T curve in Fig S5. **This reduction in M_S, however, does not significantly affect the damping of the single layer LSMO.** In Fig R1, we show the linewidth vs. frequency at 300K of two NGO (110)/LSMO single layer samples, both grown at 950C but annealed at 950C ($LSMO_{950}$) and 710C ($LSMO_{710}$) respectively (identified by the subscript), while keeping all other deposition conditions the same. We observe that the damping value $LSMO_{710}$ is statistically the same as $LSMO_{950}$.

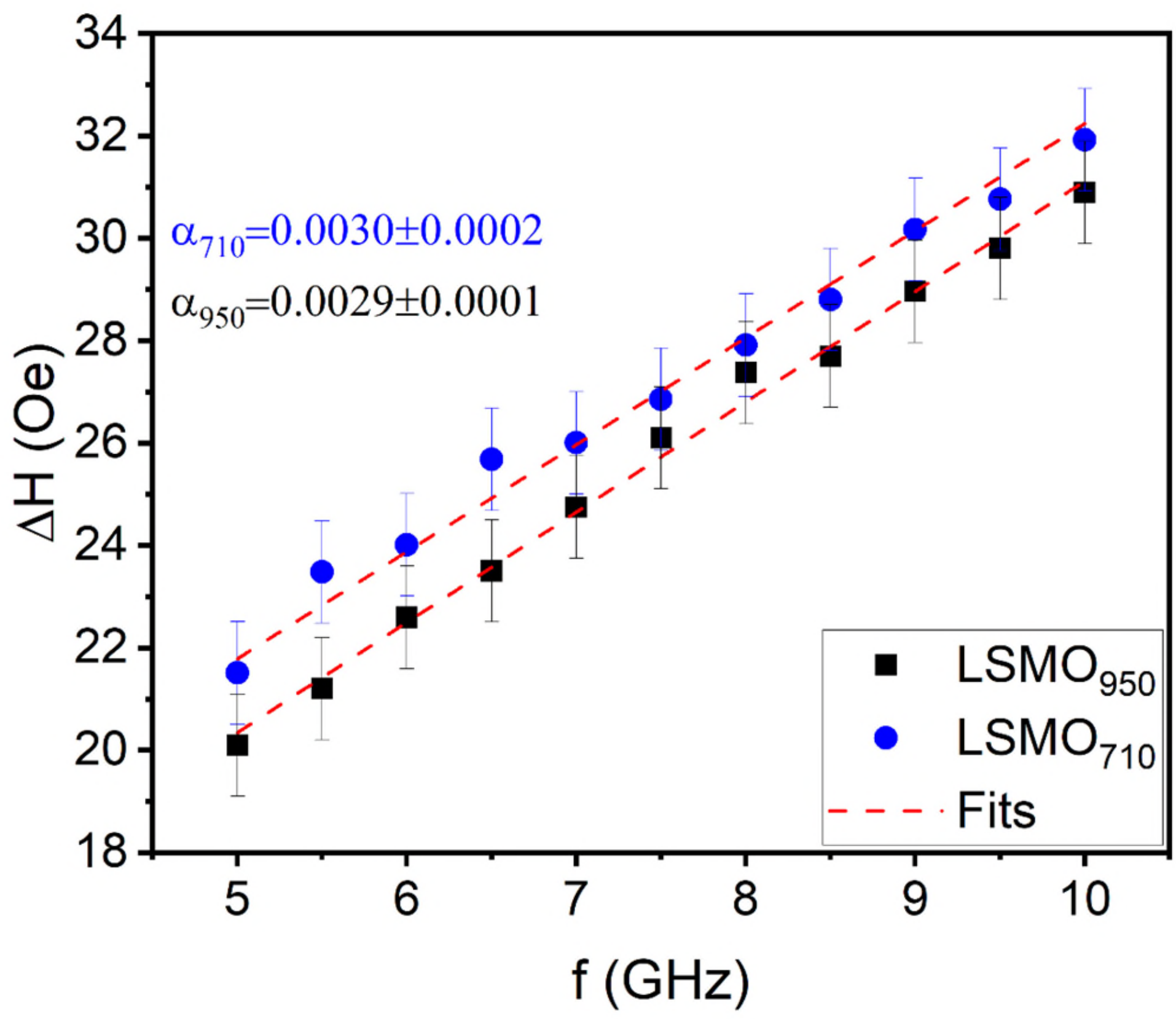

Figure R1 Comparison of damping of LSMO single layer films grown at 950 but annealed at 950C and 710C demonstrating similar damping values at 300K.

It is to be noted that while the $\alpha_{Bilayer}$- α_{LSMO} is an important parameter for SHA estimation, the symmetric SP-FMR voltage is the more important quantity as it is the

direct indication of the spin-charge conversion via inverse spin Hall effect. Single layer NGO (110)/LSMO sample does not show any such behaviour. We have reproduced such a plot from Ref 1 in Fig R2. It is very clear that unlike bilayer samples, there is a distinct absence of a strong symmetric component in single layer NGO (110)/LSMO film, demonstrating the dominant influence of NNO on the spin-charge conversion in the bilayer.

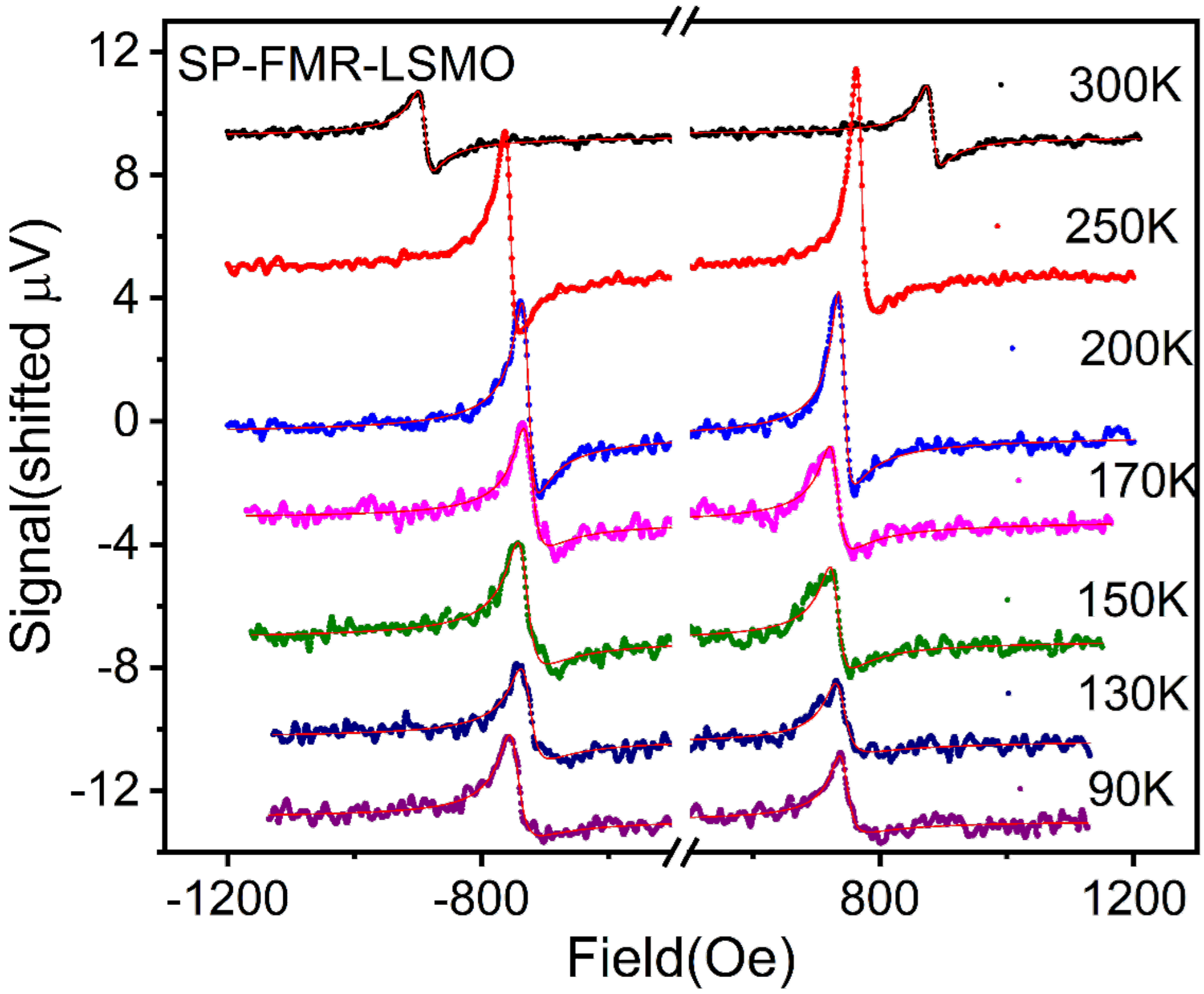

Figure R2 SP-FMR signal of single layer NGO(110)/LSMO film at different temperature showing absence of spin pumping signal. Reproduced from ref. 1.

In summary, while the different growth conditions affect the magnetization of the LSMO, the structural and chemical composition remains virtually unaffected. Despite the difference in M_S, the damping remains statistically the same and the observed damping enhancement is primarily due to spin pumping effect of NNO.

We have now made the necessary changes in the revised manuscript (Page 7 line 16). Figure R1 has been included in Figure S6(c).

Reviewer #2 and 4 (Remarks to the Author):

After reviewing the revised manuscript and the ref. response, I believe my questions have been adequately addressed, and the manuscript is suitable for publication.

We thank the reviewers for their favourable opinion of the revised manuscript and its suitability for publication in *Nature Communications*.

Reviewer #3 (Remarks to the Author):

The revised manuscript is well written and has clearly improved in response to the previous review. The authors have adequately addressed the comments, and the work appears technically sound and ready for publication.

Nevertheless, the study remains largely descriptive and does not provide sufficient microscopic insight or conceptual novelty to justify publication in Nature Communications. The results do not constitute a broadly impactful or unexpected advance for the journal's wide readership.

In summary, while the manuscript is suitable for publication in its current form, it would be more appropriate for a specialised journal in spintronics or condensed-matter physics rather than Nature Communications.

We thank the reviewer for their constructive feedback and for acknowledging that the revised manuscript is well-written, technically sound, and clearly improved. However, we respectfully disagree with the assessment that the study lacks the conceptual novelty or microscopic insight required for *Nature Communications* for several key reasons.

To put into context, enhancement of spin pumping and related dynamics is an important field of research as is evidenced in the case of IrMn[2] or CoO, NiO[3] systems. These systems have a second order magnetic phase transitions where enhancement in the spin pumping occurs near the Neel temperature due to increased spin susceptibility fluctuations.

In contrast NNO has a first order coupled magnetic/electronic phase transition. While metal-insulator transitions (MIT) have been studied in other systems like VO_2, those materials typically show a monotonic decrease in spin-to-charge conversion efficiency as they become insulating. In stark contrast, our work reveals a pronounced enhancement of the inverse spin Hall effect (ISHE) at the onset of the phase transition. This is a fundamentally new phenomenon that has not been observed in other first order MIT/ phase transition systems, owing to the highly correlated behavior of complex oxides which allows coupling between spin, charge and structural degrees of freedom.

In the revised manuscript, our study goes beyond mere description by providing a specific microscopic mechanism for this enhancement. We employ resonant soft x-ray scattering to demonstrate domain formation in NNO in the LSMO/NNO bilayer and quantify the domain size in this system. Through complementary electrical measurements and analysis of single layer NNO film, we further provide quantitative

insights into the insulating domain size (~9-18 nm) and the volume fraction of the insulating domains at the onset of phase transition, which we determine to be ~1-4%. We assert that the enhancement in SHA is due to disorder-driven enhanced spin scattering by these sparse domains. The underlying theory has been studied in Pt with extrinsically doped insulating impurities, which demonstrate such enhancement when the dopant percentage varies between 1-3%[4–6]. In our case, insulating domains are formed intrinsically and we can probe their effect seamlessly through the phase transition criticality in a single sample. Our work sheds light on the previously unexplored spin dynamics in NNO, which has conventionally been known for its MIT phase transition, for use in neuromorphic computing devices, and unveil a new tuning knob — the previously unexplored spin-based route, which is coupled to the electronic phase transition, something which surpasses the limitations of all-metallic systems and making it relevant to the broader research community, not just in spintronics.

We believe this will open new ways to employ such coupled oxide-based systems for uses in reconfigurable, energy efficient spintronic devices and neuromorphic computing, offering a conceptual advance that clearly extends beyond the scope of specialized journals to the broader readership of *Nature Communications*.

Based on reviewer's concerns, we have taken the opportunity to highlight the uniqueness of our work in the present revised manuscript in **lines 17-22; 36-40 in Page 10, Lines 1-16;23-43 in Page 11 and Lines 3-7 in Page 12.**

References:

1. Sahoo, B. *et al.* Temperature Dependent Spin Dynamics in $La_{0.67}Sr_{0.33}MnO_3$/Pt Bilayers. *Advanced Materials Interfaces* **12**, 2401038 (2025).
2. Frangou, L. *et al.* Enhanced Spin Pumping Efficiency in Antiferromagnetic IrMn Thin Films around the Magnetic Phase Transition. *Phys. Rev. Lett.* **116**, 077203 (2016).
3. Qiu, Z. *et al.* Spin-current probe for phase transition in an insulator. *Nat Commun* **7**, 12670 (2016).
4. Zhu, L., Zhu, L., Sui, M., Ralph, D. C. & Buhrman, R. A. Variation of the giant intrinsic spin Hall conductivity of Pt with carrier lifetime. *Science Advances* **5**, eaav8025 (2019).

5. Wang, Y. *et al.* Enhancement of spintronic terahertz emission enabled by increasing Hall angle and interfacial skew scattering. *Commun Phys* **6**, 1–10 (2023).

6. Xu, X. *et al.* Giant Extrinsic Spin Hall Effect in Platinum-Titanium Oxide Nanocomposite Films. *Advanced Science* **9**, 2105726 (2022).

Author comments/replies are given in blue and green.

All the changes have been marked in red in the revised manuscript.

Reviewer #1 (Remarks to the Author):

I thank the authors for their additional and very important data comparing the damping of LSMO grown at 950 °C and + annealed at 950 °C with those of the LSMO grown at 950 °C + annealed at 710 °C (“simulating” the condition of the functional bi-layer sample). The newly added Fig. S6(c) in the Supp. Info, fully answers my latest concern, and I am more than happy to suggest the final acceptance of the manuscript at Nature Communications.

We thank reviewer 1 for their important suggestion which helped us address this point and improve the manuscript.

Reviewer #5 (Remarks to the Author):

I was invited as a fourth referee to evaluate the second revised version of the manuscript by Dr. B. Sahoo and co-authors, with access to the previous referee reports and the authors’ responses.

The manuscript is clearly written and addresses a relevant topic in the field of correlated oxides. The central observation, the change in spin-to-charge conversion (S2CC) in $NdNiO_3/La_{0.67}Sr_{0.33}MnO_3$ heterostructures across the metal–insulator transition (MIT) in $NdNiO_3$, is interesting and potentially of significance.

However, I share the concern raised by Referee #3 that the study remains largely descriptive. While I do not consider this alone as a reason for rejection, the manuscript currently falls short of the level of explanation, typically supported by some form of calculation, expected for publication at this level.

Moreover, several claims appear overstated. I will focus on one representative example: the statement that the work “provides new insights into the interplay of the NNO phase transition with the LSMO magnetization dynamics” (line 86) is not fully supported by the presented data. Similar statements also appear in the conclusions/summary. Establishing such an interplay would require a more systematic investigation of the magnetic properties of the LSMO layer (e.g., via thickness or doping dependence) to directly probe its magnetic and transport response.

Another aspect that should be more carefully addressed is the quality of the $NdNiO_3$ layer. The R(T) data (Supplementary Fig. S3) show a broad transition with no clear

hysteresis, suggesting that the film may not be of "optimal" quality (i.e., lacking a sharp MIT transition and well-defined hysteresis). While this does not invalidate the results, it should be more explicitly discussed, particularly in relation to phase coexistence and disorder effects. The authors partially address this point through resonant soft X-ray scattering and additional transport data indicating a relationship between insulating and metallic domains and their formation; however, the underlying mechanisms and implications are not fully developed.

Overall, the manuscript presents an interesting experimental observation; however, the level of interpretation should be further refined. I therefore recommend publication after revision, provided that the authors moderate their claims and more carefully align their discussion and conclusions with the presented experimental evidence.

We thank the reviewer for allowing us the opportunity to address the issues in the revised manuscript. Based on their recommendation, we have now performed several calculations (Page 8 lines 14-21) and their interpretation (Page 10 Lines 1-19 and Page 11, Lines 1-22) namely:

1) Calculation of the relative carrier lifetime τ through the phase transition (Figure 5(a)).
2) Calculation of the interfacial spin transparency across the phase transition (Figure 5(b)).

Additionally, we calculate the apparent spin hall conductivity and its variation with longitudinal conductivity to show the influence of the extrinsic bulk dependent SHE (Figure S11(c-d)). Based on the variation of these quantities we show that the extrinsic SHE increases with the bulk NNO in accordance with the Dyakonov-Perels mechanism.[1,2] This extrinsic effect may mask the enhanced spin scattering at the phase transition, which occurs due to reduction in the carrier lifetime by insulating impurities in the intrinsic regime. However, upon reducing the NNO thickness we reduce this bulk contribution and observe the carrier lifetime reduction for NNO(8) and NNO(4) samples which reinforces our original assertion. For NNO(4) the enhancement is not clearly observed as the T_{MIT} is 95K—for temperatures below 110K there is significant dissipation of spins in the bulk and interface of LSMO[3]. This, in addition to the small extrinsic contribution of NNO(4), reduces the resultant voltage. Additionally, we observe an improvement in the spin transparency at the T_{MIT} which can allow for better transmission of spins into NNO, thereby increasing the I_{C-SP} at the phase transition. Previous studies involving second order phase transition from para magnetic to AFM ordering[4] and the insertion of AFM NiO layer between metallic FM/HM structures has been shown to allow increased spin transparency and fully transparent spin interfaces.[5–9] Thus, the intrinsic scattering and the increased spin transparency work in tandem to enhance the SHA at T_{MIT}.

----------------************-----------------****************------------------

We appreciate the referee's insightful observation with regards to the quality of the NNO film based on the R-T Hysteresis loop. We respectfully push back on the inference that the transition breadth indicates suboptimal film quality, while agreeing that the issue deserves more explicit treatment in the text:

1. Since we are growing bilayers in this experiment, we had to optimize deposition conditions for both LSMO and NNO so that we can have sufficient MIT nature of NNO while preserving the FM nature of LSMO. Thus, we had to tweak our deposition parameters from the ideal values of LSMO and NNO individually to obtain both MIT of NNO and FM of LSMO on the bilayers. This point was raised for LSMO single layer by reviewer #1, which we addressed in the previous revision, wherein single layer LSMO had a higher saturation magnetization (with similar R(T)) as opposed to the bilayer case. Despite this, the overall parameters such as damping were not significantly affected, allowing for a viable analysis. Structurally, the RSMs along with the TEM (with EELS) indicate a uniformly strained epitaxial bilayer with atomically clean interface with all the necessary elements present demonstrating high quality sample growth. Further, increase of damping and coercivity in the insulating phase provides us definite signatures of magnetic coupling between NNO and LSMO.

For a fair comparison, we had shown the behavior of single layer NNO deposited with these tweaked parameters in Fig S3 in supplementary info. For the referee's consideration, we present the R-T of the optimized deposition parameter for single layer NNO in Figure R1 here, which shows the sharp hysteresis similar to that available in literature[10,11].

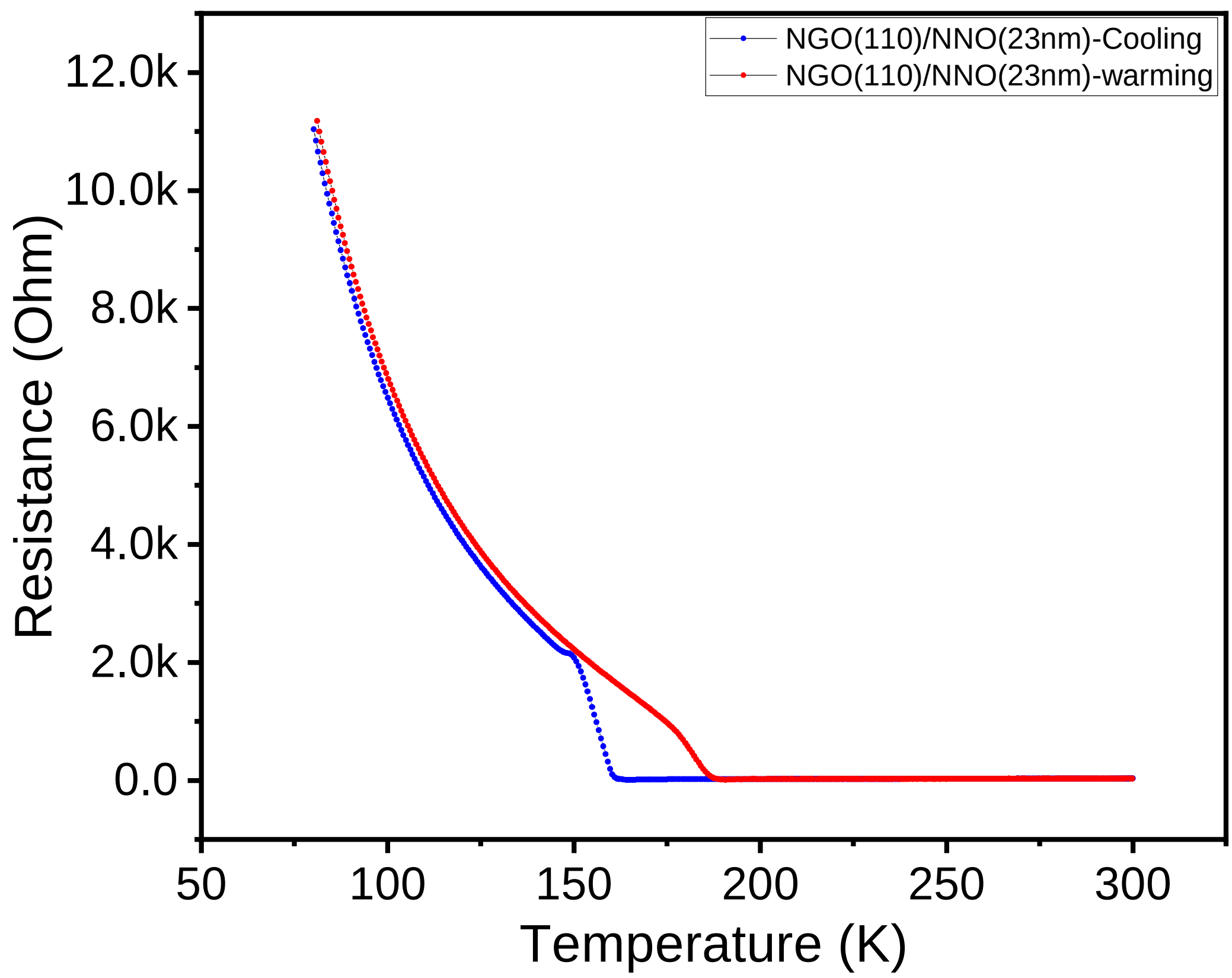

Figure R1 R(T) of single layer NNO grown in the optimized deposition parameter.

2. While the tweaked deposition parameter does affect the sharpness of the transition, the transition width, the hysteresis amplitude in our R(T) for the bilayers are consistent with established behavior of NdNiO3 films of comparable thickness on tensile substrates, [10,11] albeit with a slightly broader hysteresis and reduction of the transition temperature (Fig. R2). Independent of the R(T) behavior, the first-order character is confirmed magnetically via observation of the $(\frac{1}{4}\,\frac{1}{4}\,\frac{1}{4})_{pc}$ AFM Bragg peak measured by RSXS, which shows clear, well-resolved hysteresis on warming and cooling (Fig. 4(a)). This is a direct fingerprint of the transition complementing the bulk-averaged R(T)[12].

[FIGURE REDACTED]

Figure R2 (a) shows the hysteresis window as defined in Mattoni et al [10]. (b) is the hysteresis window for our case. For both cases NNO thickness is around ~16nm. Our hysteresis window (33K) is slightly larger than that presented in this reference according to the convention followed in this reference.

We have added the following discussion in the main text near **Fig. 1(e), Page 4 Lines 15-20:**

"We note that the MIT in our films is less sharp, with less pronounced hysteresis (Fig. S3(a)), compared to reports on optimally grown single-layer $NdNiO_3$.[10,11] This is because our deposition parameters were chosen to preserve simultaneously the MIT in NNO and the low-damping FM state of LSMO in the bilayer. Hence, the NNO layer is not optimized by single-layer standards. Nonetheless, the first-order character of the transition is independently and unambiguously confirmed by the resolvable hysteresis in the $(¼\ ¼\ ¼)_{pc}$ AFM Bragg peak (Fig. 4(a)) via resonant soft x-ray scattering, discussed below."

----------------************-----------------****************-----------------

We agree with the Referee in relation to aligning our claims and interpretations based on the experimental proof presented in the manuscript. A systematic LSMO thickness or doping series is beyond the scope of the present work, and the original wording overreaches what our single-LSMO-thickness dataset establishes. We have moderated the language in both the Introduction and the Conclusion:
Introduction (currently ~line 4-6, page 3):

Original: "Our findings provide new insights into the interplay of the NNO phase transition with the LSMO magnetization dynamics and reveal the transition's role in modulating spin currents."

Revised: " Our findings reveal how the NNO phase transition modulates spin transport across the LSMO/NNO bilayer and identify mesoscopic domain heterogeneity in NNO as an underlying driver of this enhancement.

We have made similar such changes in wording throughout the manuscript to be in line with data and their relevant interpretation.

References

1. D'Yakonov, M. I. & Perel, V. I. Spin Orientation of Electrons Associated with the Interband Absorption of Light in Semiconductors. *Journal of Experimental and Theoretical Physics* **33**, 1954 (1971).

2. Boross, P., Dóra, B., Kiss, A. & Simon, F. A unified theory of spin-relaxation due to spin-orbit coupling in metals and semiconductors. *Sci Rep* **3**, 3233 (2013).

3. Sahoo, B. *et al.* Temperature Dependent Spin Dynamics in $La_{0.67}Sr_{0.33}MnO_3$/Pt Bilayers. *Advanced Materials Interfaces* **12**, 2401038 (2025).

4. Frangou, L. *et al.* Enhanced Spin Pumping Efficiency in Antiferromagnetic IrMn Thin Films around the Magnetic Phase Transition. *Phys. Rev. Lett.* **116**, 077203 (2016).

5. Zhu, L. Fully Spin-Transparent Magnetic Interfaces Enabled by the Insertion of a Thin Paramagnetic NiO Layer. *Phys. Rev. Lett.* **126**, (2021).

6. Lee, K. *et al.* Superluminal-like magnon propagation in antiferromagnetic NiO at nanoscale distances. *Nat. Nanotechnol.* **16**, 1337–1341 (2021).

7. Moriyama, T. *et al.* Anti-damping spin transfer torque through epitaxial nickel oxide. *Appl. Phys. Lett.* **106**, 162406 (2015).

8. Sahoo, B. *et al.* High-Efficiency Continuous Spin-Conduction through NiO/Cu Bilayer Structure. *Nano Lett.* **25**, 3851–3857 (2025).

9. Hung, Y.-M. *et al.* Spin transport in antiferromagnetic NiO and magnetoresistance in Y3Fe5O12/NiO/Pt structures. *AIP Advances* **7**, 055903 (2016).

10. Mattoni, G. *et al.* Striped nanoscale phase separation at the metal–insulator transition of heteroepitaxial nickelates. *Nat Commun* **7**, 13141 (2016).

11. Ojha, S. K. *et al.* Anomalous electron transport in epitaxial $NdNiO_3$ films. *Phys. Rev. B* **99**, 235153 (2019).

12. Li, J. *et al.* Scale-invariant magnetic textures in the strongly correlated oxide $NdNiO_3$. *Nat Commun* **10**, 4568 (2019).

Author comments/replies are given in green.

Reviewer #5 (Remarks to the Author):

The authors have made substantial efforts to revise and improve the manuscript and have addressed the majority of my concerns in a satisfactory manner. While a few minor questions remain, I believe that the manuscript presents valuable results that will be of interest to the community.

Therefore, I support its publication in the present form.

We thank the reviewer for their critique, advice and suggestions that led to the perfecting of the manuscript form suitable for Nature Communications.

Enhanced spin-to-charge conversion in $La_{0.67}Sr_{0.33}MnO_3/NdNiO_3$ bilayers at the nickelate metal-insulator phase transition

Biswajit Sahoo, Sarmistha Das, Akilan K, Alexandre Pofelski, Sebastien Petit-Watelot, Juan Carlos Rojas-Sánchez, Yimei Zhu, Alex Frano, and Eric E Fullerton

---

NNO is of interest due to a phase transition, from paramagnetic metal to antiferromagnetic insulator at a given transition temperature. This can be controlled in a number of ways, including electron doping, strain engineering or thickness variation. In this work, the authors investigate the effect of such phase transition into the spin dynamics of the bilayer LSMO/NNO. To do so, the authors investigate the spin-to-charge conversion in a range of LSMO/$NdNiO_3$ bilayers with three controlled NdNiO (NNO) different thicknesses. The quality of the samples is proved via HTEM imaging and the metal-insulator properties are checked with resistivity curves. Spin-to-charge conversion is measured for all bilayers at different temperatures using ferromagnetic resonance.

The main experimental result is a signature of an enhanced spin pumping as the temperature approaches the transition temperature. At this transition, the authors argue that insulating antiferromagnetic domains or clusters embedded into the still metallic bulk (as it is below the the transition temperature) act as extrinsic scatterers. As the temperature further increases, the spin pumping goes back to its original value. This point, which is the main result of this work, it is unclear to us. As the NNO transits from metallic to insulating phases, the spin to charge conversion should also change, from one side, the metallic phase can hosts 'spins' whereas the insulating phases, one should have 'magnons'. In view of figure 3 (a), it seems that in both phases have the same spin-to-charge or magnon-to-charge efficiency. Thus, it is not evident to us that at the transition, the presence of insulating phase domains would enhance this efficiency.

The introduction of nickelates as a way of modulating the properties of a spin-charge conversion device via temperature changes is novel but the article lacks a neat result and is rather descriptive in nature. We believe that a deeper analysis of the experimental results is necessary in order to claim an important, novel result which is among the criteria for publication in Nature Communications.

The manuscript is a bit confusing and needs of an improvement of the presentation. Some minor comments are shown below:

Main text

- Figures 1a and 1b are not referenced in the main text.
- Line 55 and Figure 1(e). "LSMO(12 nm)/NNO(0, 4, 8 and 16 nm)" The nomenclature for the LSMO without NNO on top is rather confusing. The readability would improve separating the single LSMO layer and the LSMO/NNO (4, 8 and 16 nm).
- Figure 1. The existence of the hysteresis loop in the x=4 nm bilayer is not clear as it gets easily confused with the x=16 nm curve. The colors of this figure should be improved.

- Figure S3(c). The crossing point of the derivative with the y=0 axis is not clear at all, especially seeing the noise that is present around 150 K.
- Line 135. "(full scans for all temperatures are plotted in the supplementary section)" This should point to the specific supplementary figure it is referring to.
- Line 143 and line 250. In the manuscript the spin to charge conversion is measured using the cooling cycle but as the conclusions state, these devices would be useful for a spin valve device. Some measurements or discussion about the heating cycle would add value to the manuscript.
- Line 242-244. "These emergent behaviours, driven by structural and magnetic reconstructions at the interface,..." This paragraph lacks explanation or citations.

Methods

- Sample growth. Samples are annealed at 710ºC in O. In literature it is shown that even at 100ºC nickelates develop oxygen vacancies [1]. While there is an oxygen atmosphere during annealing, XPS stochiometry measurements would be useful to check if the O pressure is sufficient.

  [1] Wang, Le ; Dash, Sibashisa ; Chang, Lei et al. / Oxygen Vacancy Induced Room-Temperature Metal-Insulator Transition in Nickelate Films and Its Potential Application in Photovoltaics. In: ACS Applied Materials and Interfaces. 2016 ; Vol. 8, No. 15. pp. 9769-9776.
- Sample growth. A brief description of how the samples were transferred to the different fabrication and measurement chambers would be adequate, as long exposures to air can lead to carbon accumulation that add to the extrinsic spin scattering.
